# Discovery and evaluation of triple inhibitors of VEGFR-2, TIE-2 and EphB4 as anti-angiogenic and anti-cancer agents

**DOI:** 10.18632/oncotarget.20065

**Published:** 2017-08-08

**Authors:** Lin Zhang, Yuanyuan Shan, Xingyue Ji, Mengyuan Zhu, Chuansheng Li, Ying Sun, Ru Si, Xiaoyan Pan, Jinfeng Wang, Weina Ma, Bingling Dai, Binghe Wang, Jie Zhang

**Affiliations:** ^1^ School of Pharmacy, Health Science Center, Xi'an Jiaotong University, Xi'an, China; ^2^ Department of Pharmacy, The First Affiliated Hospital of Xi'an Jiaotong University, Xi'an, Shaanxi, China; ^3^ Department of Chemistry and Center for Diagnostics and Therapeutics, Georgia State University, Atlanta, Georgia, United States

**Keywords:** receptor tyrosine kinase, multiple inhibitors, anti-angiogenic agents, anti-cancer agents, quinazolin-4(3H)-one

## Abstract

Receptor tyrosine kinases (RTKs), especially VEGFR-2, TIE-2, and EphB4, play a crucial role in both angiogenesis and tumorigenesis. Moreover, complexity and heterogeneity of angiogenesis make it difficult to treat such pathological traits with single-target agents. Herein, we developed two classes of multi-target RTK inhibitors (RTKIs) based on the highly conserved ATP-binding pocket of VEGFR-2/TIE-2/EphB4, using previously reported BPS-7 as a lead compound. These multi-target RTKIs exhibited considerable potential as novel anti-angiogenic and anticancer agents. Among them, QDAU5 displayed the most promising potency and selectivity. It significantly suppressed viability of EA.hy926 and proliferation of several cancer cells. Further investigations indicated that QDAU5 showed high affinity to VEGFR-2 and reduced the phosphorylation of VEGFR-2. We identified QDAU5 as a potent multiple RTKs inhibitor exhibiting prominent anti-angiogenic and anticancer potency both *in vitro* and *in vivo*. Moreover, quinazolin-4(3*H*)-one has been identified as an excellent hinge binding moiety for multi-target inhibitors of angiogenic VEGFR-2, Tie-2, and EphB4.

## INTRODUCTION

Pathological angiogenesis is widely involved in tumor progression and has been recognized as a defining characteristic of cancers [1]. Solid tumor growth depends on the vascular vessels to supply oxygen and nutrients and its progression is accompanied with rich angiogenesis [2]. The understanding of angiogenesis has given rise to novel treatment options for cancers. Anti-angiogenic agents are effective in suppressing tumor growth and metastasis [3]. Cancer cells secrete various pro-angiogenic factors such as vascular endothelial growth factors (VEGFs) and its receptor (VEGF Receptor-2, VEGFR-2), angiopoietin (Ang) and its receptor (tyrosine kinase with Ig and epidermal growth factor homology domain-2, Tie-2), erythropoietin-producing hepatoma receptor B4 (EphB4) and its membrane-associated ligand, ephrinB2 [4–6]. However, it is a known fact that many anti-angiogenic agents have failed in the clinic trials. Recently, mounting evidence indicates that tumors become refractory or even bypass the inhibition of a single pro-angiogenic factor via compensatory activation of alternative pro-angiogenic factors [7]. Therefore, systemic characterization of compensatory activation profile constitutes critical steps towards a rational design of multi-target anti-angiogenesis agents. Thus, we undertook the effort to design inhibitors capable of inhibiting multiple targets critical for angiogenesis.

## DESIGN STRATEGY OF MULTI-TARGET RTK INHIBITORS

**Figure 1:**
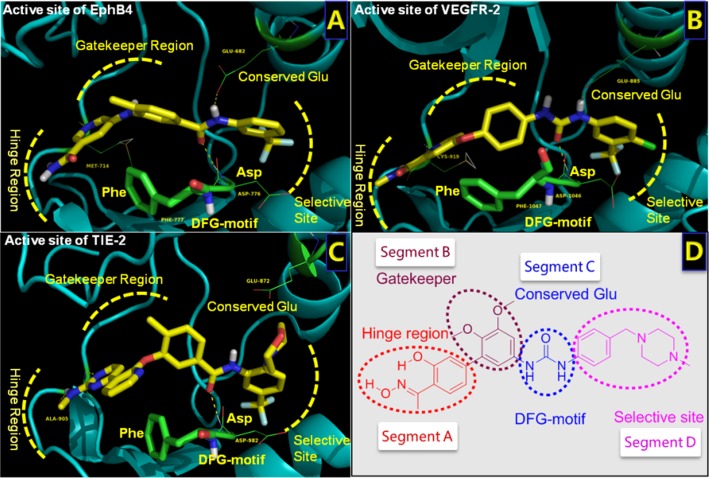
Structures of the active sites of three RTKs bound with co-crystalized inhibitors **A**. Structure of EphB4 complexed with 32W (PDB ID: 4BB4). **B**. Structure of VEGFR-2 bound with sorafenib (PDB ID: 4ASD). **C**. Structure of Tie-2 complexed with MR9 (PDB ID: 2P4I). **D**. Structure and pharmacophore of lead compound (BPS-7). Critical elements of inhibitor interaction are labeled and highlighted.

We are interested in the discovery of novel anti-angiogenesis agents. Along this line, structural optimization of natural alkaloid taspine afforded a salicylaldoxime including BPS-7 as potent VEGFR-2 inhibitors (Figure 1D) [8–10]. Very importantly, BPS-7 displayed potent inhibition against angiogenic EphB4 and Tie-2 [11]. These findings encouraged us to develop novel triple inhibitors of VEGFR-2/TIE-2/EphB4 as anti-angiogenic and anticancer agents. Moreover, structural similarity and the highly conserved conformations of VEGFR-2, Tie-2, and EphB4 offer the possibility of designing multi-target inhibitors [12]. The highly conserved aspartate-phenylalanine-glycine (DFG-motif) triad activation loop is essential for kinase activity [13]. Moreover, there is a substantial degree of compensatory activation among three RTKs [14–18]. Therefore, simultaneous inhibition of them might be a promising approach for the suppression of angiogenesis. This kind of multiple RTKIs have a major advantage of overcoming the compensatory feedback of the single-target angiogenesis inhibitors. The three angiogenic RTKs share a basic architecture for construction (Figure 1A-1C), namely hydrogen bonds with the hinge region, hydrophobic interactions with the gatekeeper region, hydrogen bonds with the conserved Glu and DFG motifs and hydrophobic interactions with the allosteric site [19].

**Figure 2:**
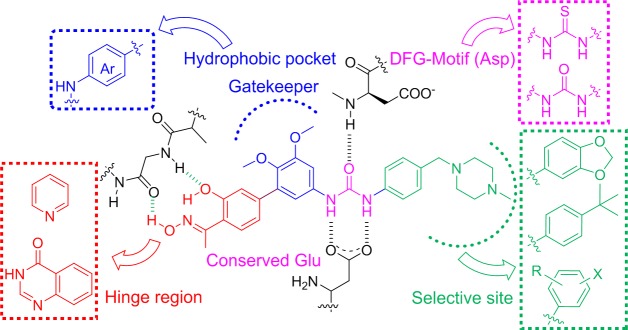
Design strategy and structures of novel multi-target anti-angiogenesis agents derived from BPS-7

Previously, diaryl urea compound BPS-7 was identified as privileged scaffold. Our continuous efforts focused on the development of novel anti-angiogenic agents based on the binding mode of BPS-7 to RTKs. In doing so, we dissected the lead into four regions for optimization work (Figure 2). First, we explored various heteroaromatics as hinge binding group (HBG) via core-refining approach. Pyridine and quinazolin-4(3*H*)-one were introduced as new HBG. We reasoned that it is possible that these groups could form hydrogen bonds with RTKs and therefore provide an opportunity to improve affinity. Second, the two methoxyl groups on biphenyl of BPS-7 were removed to reduce the steric hindrance in binding with the respective receptor. Third, inspired by the classic bioisosteric paradigm, urea moiety was replaced with thiourea which bears hydrogen bond donors and acceptors. Fourth, various anilines bearing halogen were incorporated as they are beneficial for antitumor potency and could enhance the persistence [20]. In addition, other anilines containing *tert*-butyl or benzo[*d*] [1,3]dioxole were also incorporated as fragments interacting with selective site of RTKs.

These two series of compounds were synthesized, and evaluated for their ability to inhibit RTKs, and then subsequently for their anti-angiogenic and anticancer properties. The representative QDAU5 bearing a quinazolin-4(3*H*)-one moiety displayed prominent anti-angiogenic and anticancer potency both *in vitro* and *in vivo*.

## RESULTS AND DISCUSSION

### Chemistry

**Scheme 1:**
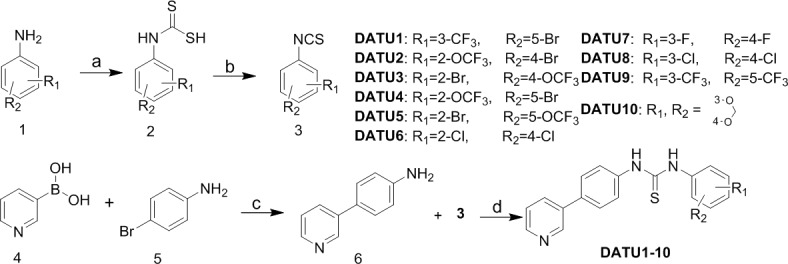
Synthesis of diaryl thiourea derivatives DATU1-10^a^ ^a^Reagents and conditions: (a) Dabco, CS_2_, toluene; (b) BTC, DCM; (c) Pd(PPh_3_)_4_, K_2_CO_3_, dioxane, 100°C; (d) DCM, room temperature.

Scheme 1 depicts the synthesis of diaryl thiourea derivatives DATU1-10. Various commercial available anilines 1 were treated with CS_2_ to generate 2, which was treated with bis(trichloromethyl) carbonate (BTC) to afford isothiocyanates 3. The key intermediate 6 was prepared from 3-pyridinylboronic acid 4 and *para*-bromoaniline 5 by Pd-catalyzed Suzuki coupling reaction without further purification. Finally, these isothiocyanates 3 were reacted with biphenyl intermediates 6 to afford the title compounds DATU1-10.

**Scheme 2:**
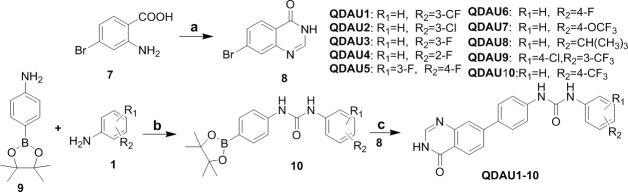
Synthesis of quinazolinone diaryl urea derivatives QDAU1-10^a^ ^a^Reagents and conditions: (a) HCONH_2_, MW, 150°C,1.5h; (b) BTC, Et_3_N, 0°C to room temperature; (c) Pd(PPh_3_)_4_, K_2_CO_3_, H_2_O/dioxane, 100°C.

The synthetic route of quinazolinone diaryl urea derivatives QDAU1-10 is shown in Scheme 2. 2-Amino-5-bromobenzoicacid 7 was converted to intermediate 8 by cyclization with formamide under atmospheric microwave heating at 150°C. The coupling substrates 10 were obtained by condensation of amino-boronate 9 with various isocyanates, which were prepared from reaction of anilines with BTC. Subsequently, the title commands QDAU1-10 were prepared from the key intermediates 8 and 10 by Suzuki coupling reaction (See Supplementary Materials).

### Biological evaluation

The three targeted RTKs are involved in both angiogenesis and tumorigenesis [21]. With the aim of developing anti-angiogenic and anticancer agents, we firstly evaluated the title compounds for their inhibitory effect and selectivity among various RTKs. Subsequently, in order to validate the dual function of the title compounds, their inhibition on viability of human endothelial cells (HECs, EA.hy926) was examined to examine the anti-angiogenic potency. In addition, their binding affinity to angiogenic RTKs and anticancer potency were also studied in different models. A marketed multi-target RTK inhibitor, sorafenib, was used as positive control.

### *In Vitro* receptor tyrosine kinases inhibition assay

In order to validate the direct effect of title compounds on VEGFR-2, Tie-2, and EphB4 kinases, we performed *in vitro* assay using the ADP-Glo Kinase Assay Kit (Promega, Wisconsin, USA). In analyzing the inhibition data, we first look at the implications of replacement with thiourea and the hinge binding group of BPS-7. Meanwhile, pyridine was introduced as hinge binding groups (Table 1). It was obvious that compounds with *di*-halogen substituents (DATU6-8) were more potent than the others. DATU6 displayed the most potent RTKs inhibitory activities with IC_50_ values of 23.5 nM (VEGFR-2), 66.5nM (Tie-2), and 3.86 nM (EphB4), respectively.

**Table 1:** Structures and RTKs inhibitory activities of title compounds (DATU1-10, IC_50_, nM)

Compound	R_1_	R_2_	VEGFR-2	TIE-2	EphB4
DATU1	3-CF_3_	5-Br	20.6	25.7	20.0
DATU2	2-OCF_3_	4- Br	>500	64.2	>500
DATU3	2- Br	4-OCF_3_	>500	20.0	21.8
DATU4	2-OCF_3_	5-Br	>500	>500	18.7
DATU5	2-Br	5-OCF_3_	>500	>500	54.2
DATU6	2-Cl	4-Cl	23.5	66.5	3.86
DATU7	3-F	4-F	45.2	48.4	10.0
DATU8	3-Cl	4-Cl	354	9.40	>500
DATU9	3-CF_3_	5-CF_3_	>500	>500	>500
DATU10	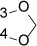		20.40	>500	>500
Sorafenib			0.55	4.65	3.00

Encouraged by the promising potency of diaryl ureas, we chose to continue our structural optimization by keeping urea unit constant. Quinazolin-4(3*H*)-one was incorporated onto diaryl urea core as a novel hinge binding group of multi-target inhibitors (Table 2). Three title compounds (QDAU4, QDAU5, QDAU9) displayed simultaneous inhibitory potency against VEGFR-2, Tie-2, and EphB4. In particular, QDAU5 bearing a 3,4-difluoroanilinegroup was the most potent multi-target RTKs inhibitors with IC_50_ values of 0.77 nM (VEGFR-2), 8.87 nM (Tie-2), and 3.21 nM (EphB4), respectively. The results highlight the importance of the quinazolin-4(3*H*)-one moiety for improving the inhibition of these multiple inhibitors. This study identified that quinazolin-4(3*H*)-one was an excellent hinge binding group for multi-target inhibitors of VEGFR-2, Tie-2, and EphB4.

**Table 2:** Structures and RTKs inhibitory activities of title compounds (QDAU1-10, IC_50_, nM)

Compound	R_1_	R_2_	VEGFR-2	Tie-2	EphB4
QDAU1	H	3-CF_3_	194	70.5	>500
QDAU2	H	3-Cl	57.9	83.9	>500
QDAU3	H	3-F	>500	90.7	5.64
QDAU4	H	2-F	1.91	4.91	13.9
QDAU5	3-F	4-F	0.77	8.87	3.21
QDAU6	H	4-F	6.28	101	>500
QDAU7	H	4-OCF_3_	>500	258	>500
QDAU8	H	-CH(CH_3_)_2_	41.5	>500	>500
QDAU9	4-Cl	3-CF_3_	15.8	19.6	24.4
QDAU10	H	4-CF_3_	50.7	52.7	>500
Sorafenib			0.55	4.65	3.00

### Kinase selectivity assays

We investigated the kinase selectivity of the two most potent compounds (QDAU4, QDAU5) against other seven kinases including VEGFR-1, VEGFR-3, FGFR-1, FGFR-4, B-Raf, EGFR and c-Kit for its selective profile. The results were summarized in Table 3. The results revealed that they showed less potency against these RTKs compared with VEGFR-2, TIE-2, and EphB4 with IC_50_ values higher than 50 nM. It was indicated that they exhibited moderate selectivity for VEGFR-2/TIE-2/EphB4 relative to other RTKs.

**Table 3:** RTKs selectivity profile of the most active compounds (IC_50_, nM)

Compound	VEGFR-1	PDGFR-β	FGFR-1	FGFR-4	B-Raf	EGFR	c-Kit
QDAU4	68.3	>500	165.9	83.4	136	113	>500
QDAU5	192	98.5	176.2	>500	>500	98.8	142

### Effects on the viability of human endothelial cells (HECs)

In order to determine the anti-angiogenic effect of these multi-target inhibitors, we evaluated their effect on HECs (EA.hy926) viability using cell counting kit-8 (CCK-8) method [22]. Highly consistent with their potent RTKs inhibitory activity, the majority of the selected compounds displayed dose-dependent inhibition of EA.hy926 viability with IC_50_ values ranging from 4.00 μM to 255 μM (Table 4). Notably, three compounds (DATU1, QDAU4, and QDAU5) exhibited more potent inhibition on the viability of HECs than the others with IC_50_ values less than 10 μM. These results demonstrated that these compounds could display anti-angiogenic potency through decreasing viability of HECs (EA.hy926). In addition, we profiled the most potent compound, QDAU5, in the anti-proliferative assays against human normal cells. We identified the cytotoxicity of QDAU5 on normal human liver cells (QSG7701) and human embryonic kidney 293 (HEK293). In summary, the results indicated that QDAU5 displayed selective growth inhibition of EA.hy 926 over two human normal cells (IC_50_ values > 100 μM). From this we might conclude that the representative QDAU5 exhibit selective growth inhibitory activity against HECs compared to normal human cell lines.

**Table 4:** Inhibition of compounds on human endothelial cell viability (IC_50_, μM)

Compound	EA.hy926	Compound	EA.hy926	Compound	EA.hy926
DATU1	3.96	DATU8	102	QDAU5	2.69
DATU2	89.9	DATU9	166	QDAU6	255
DATU3	122	DATU10	90.6	QDAU7	68.5
DATU4	44.8	QDAU1	57.9	QDAU8	76.2
DATU5	246	QDAU2	66.3	QDAU9	13.0
DATU6	24.4	QDAU3	42.6	QDAU10	125
DATU7	96.4	QDAU4	8.64	Sorafenib	16.5

### Apoptosis detection assay

**Figure 3:**
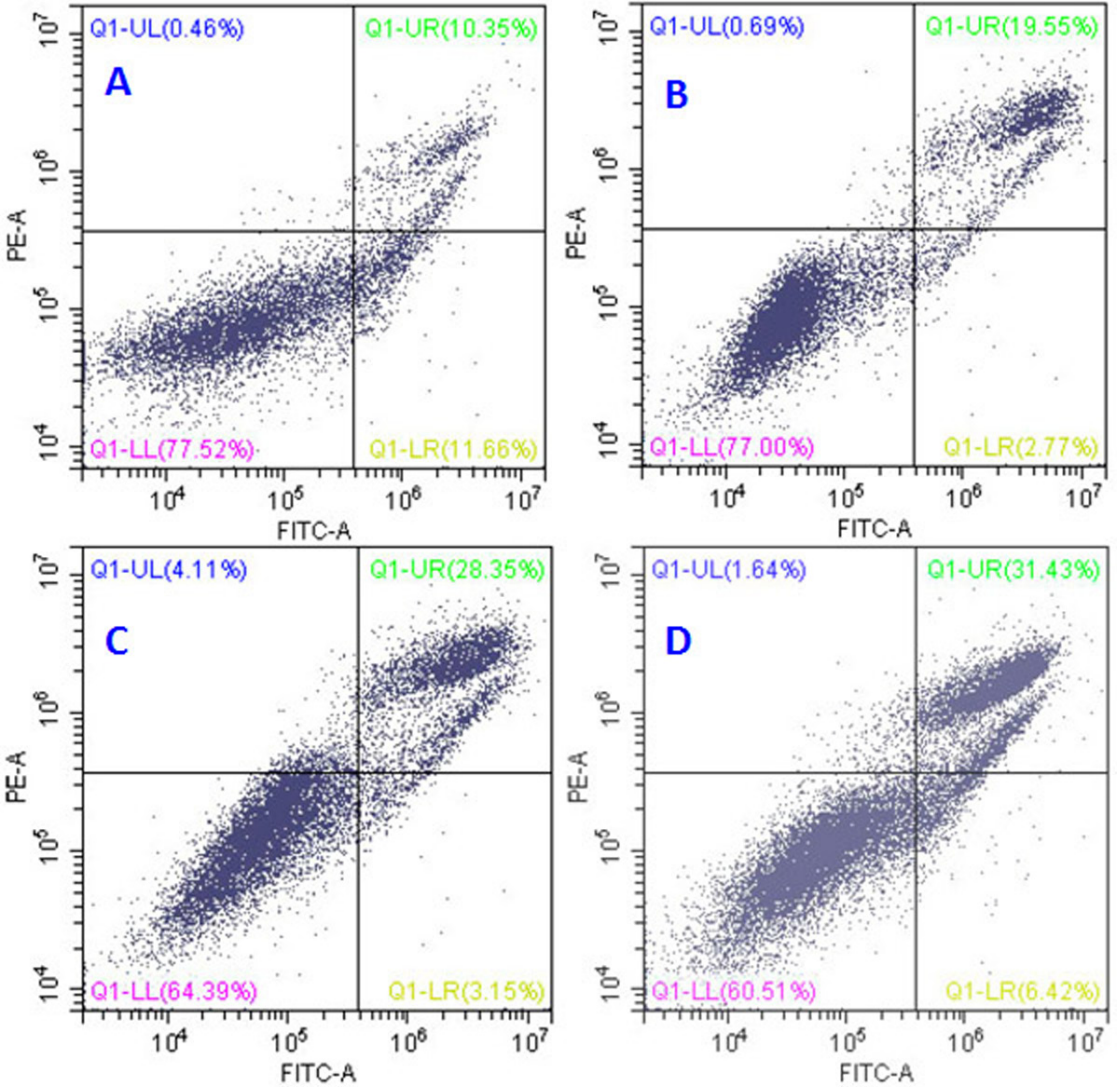
Apoptosis detection assays of EA.hy 926 cells after treatment with QDAU5 (A, 0 nM; B, 5 nM; C, 20 nM; D 40 nM)

To further investigate the effect of QDAU5 on the apoptosis of HECs (EA.hy926), we performed flow cytometry analysis and apoptosis detection assays. A substantial and dose-dependent proapoptotic activity in HECs at various concentrations for 48 h was observed. The apoptotic EA.hy926 cells (10.35%) of negative control group were depicted in Figure 3A. After treatment with QDAU5 (5, 20, and 40 nM) for 48 h, the percentage of early apoptotic cells was 19.55%, 28.35%, 31.43%, respectively (Figure 3B-3D). The results indicate that QDAU5 could induce early apoptosis of HECs (EA.hy926) in a dose dependent manner.

### Anti-proliferative activity against cancer cells

These RTKs play critical roles in the proliferation of various cancers. Therefore, RTKs inhibition generally translate well into anti-proliferation activity. In this regard, in order to investigate the potential anticancer potency of these multi-target RTKs inhibitors, the most potent inhibitor, QDAU5, was selected to examine its anti-proliferative activity against a panel of cancer cell lines including human hepatic cancer cell lines (7721), human breast cancer cell lines (MCF-7), human pancreatic cancerous cell lines (PANC-1), human epidermoid carcinoma cell line (A431), human lung cancer cell (A549), human colon carcinoma cell line (LOVO), and human cervical cancer cell line (HeLa). QDAU5 was found to significantly inhibit the growth of all seven cancer cell lines with IC_50_ values ranging from 2.12 μM to 24.9 μM (Table 5). In summary, QDAU5 displayed significant anti-proliferative activity against a broad spectrum of cancer cell lines. In addition, we identified the cytotoxicity of QDAU5 on two normal cell lines (QSG7701 and HEK293). Overall, the results indicated that QDAU5 exhibited selective growth inhibition of human cancer cell lines over two human normal cells (IC_50_ values > 100 μM). From this we might conclude that the representative QDAU5 displayed selective growth inhibitory potency against all eight human cancer cell lines compared to normal human cell lines.

**Table 5:** Anti-proliferative activity of QDAU5 against human cancer and normal cells (IC_50_, μM)

Cell line	7721	MCF-7	PANC-1	A431	A549	LOVO	HeLa	QSG7701	HEK293
QDAU5	58.7	2.12	24.9	16.9	8.37	4.99	5.56	>100	>100

Because of it potent inhibition of RTKs and anti-proliferative activity, QDAU5 was further evaluated for its *in vivo* anticancer properties in a MCF-7 xenograft mice model [23]. QDAU5 treatment was initiated when tumors were palpable and continued for 21 days. In the MCF-7 xenograft model, QDAU5 could cause a significant reduction of tumor weight in a dose dependent manner (Table 6). Compared with the control group, QDAU5 inhibited tumor growth by 24% and 47% at 40 mg/kg and 80 mg/kg, respectively. Moreover, less body weight loss and no other abnormities were observed in the QDAU5-treated mice compared with controls. Such results indicate that QDAU5 is non-toxic at the doses used. It can be concluded that QDAU5 exhibited active anticancer activity with little signs of toxicity.

**Table 6:** Anticancer potency of QDAU5 in mouse xenograft models

Treatment	Route	Dose (mg/kg)	Initial body weight (g)	Final body weight (g)	Final tumor weight (g)	Tumor growth inhibition (%)
Control	ig	0	17.7±0.7	16.6±0.8	0.848±0.5	/
QDAU5	ig	20	18.7±0.8	17.4±1.0	0.787±0.7	7.2%
	ig	40	18.4±0.6	18.9±1.2	0.642±0.8	24%
	ig	80	18.3±0.7	16.1±1.0	0.449±0.9	47%
Sorafenib	ig	40	19.5±0.9	16.8±0.9	0.398±0.8	53%

### Identification of QDAU5 binding to VEGFR-2 by cell membrane chromatography method

**Figure 4:**
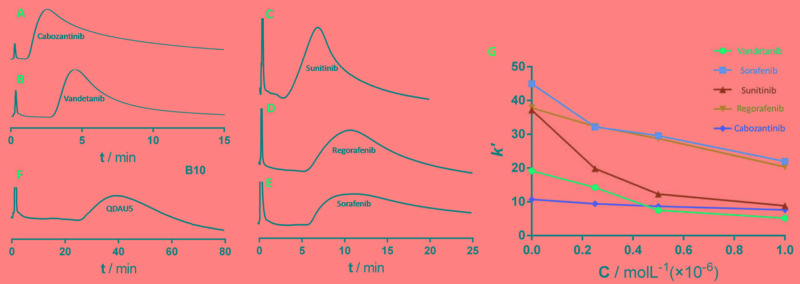
Elution profiles of cabozantinib (RT=2.6 min) **A**., vandetanib (RT=4.5 min) **B**., sunitinib (RT=6.8 min) **C**., regorafenib (RT=10.6 min) **D**., sorafenib (RT=11.4 min) **E**. and QDAU5 (RT=38.0 min) **F**. on the VEGFR-2/cell membrane stationary phase column. Chromatographic conditions: 10 mm×2.0 mm; flow rate0.6 mL/min; column temperature 37°C; mobile phase 5 mM PBS, pH 7.4; detection wavelength 270 nm. **G**. Change in retention factors of multi-target RTK inhibitors (*K’*) in the presence of QDAU5 with increased concentrations as direct competitor in the mobile phase.

The cell membrane chromatography (CMC) method has been developed as a new bioaffinity chromatographic system and been widely used to identify potent RTK inhibitors [24]. In previous studies, artificial cultures highly expressing VEGFR-2 on HEK293 cell lines were constructed. A novel analytical method coupling high-expression VEGFR-2/CMC with high performance liquid chromatography model was applied to identify ligands acting on VEGFR-2 specifically, and investigate the affinity of QDAU5 to VEGFR-2 [25]. The elution profiles of five multi-target RTKs inhibitors (cabozantinib, vandetanib, sunitinib, regorafenib and sorafenib) and QDAU5 on the high-expression VEGFR-2/CMC model are shown in Figure 4. It was found that the retention times of the inhibitors and QDAU5 were very different from each other. QDAU5 showed a longer retention time of 38 min while the retention time of the potent VEGFR-2 inhibitors, regorafenib and sorafenib, were both 11 min. One can reasonable assume that the retention on the VEGFR-2/CMC model is largely dependent on binding with VEGFR-2. In addition, the retention behavior of QDAU5 on the VEGFR-2/CMC column indicate that QDAU5 exhibited higher affinity than these multi-target inhibitors when binding toVEGFR-2.

In the direct competitive assay, five multi-target RTKIs and QDAU5 were used as competitors to examine the competition between known inhibitors and QDAU5 when binding with VEGFR-2 [26]. Figure 4G shows the change in retention factors of multi-target RTKs inhibitors (*K‘*) in the presence of QDAU5 at different concentrations as a direct competitor in the mobile phase. Decreased *K’* values of multi-target RTKs inhibitors was observed with increasing concentrations of QDAU5 in the mobile phase. The displacement studies indicated that QDAU5 interacted with the same site of VEGFR-2 with that of the five multi-target RTKIs.

**Figure 5:**
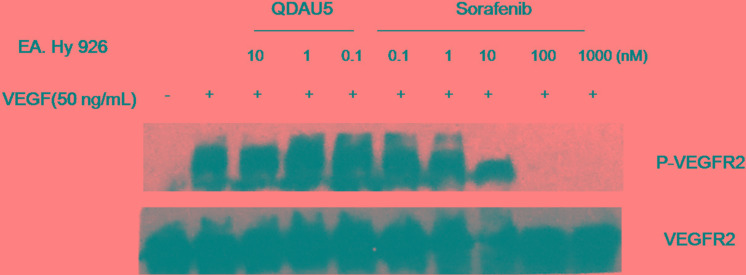
Effect of QDAU5 on the level and phosphorylation of VEGFR-2 in EA.hy926 cells

We further evaluated the effect of representative QDAU5 on the expression level and phosphorylation of VEGFR-2 in HECs using western blot assay. EA.hy926 cells were treated with QDAU5 for 48 h followed by 50 ng/mL VEGF stimulation for 10 min. It was found that QDAU5 dose-dependently decreased VEGF-induced tyrosine phosphorylation of VEGFR-2 in EA.hy926 cells compared with the negative control group (Figure 5). In addition, it moderately reduced the level of VEGFR-2 in VEGF-stimulated EA.hy926 cells. Our findings suggested that the influence of QDAU5 on cell viability of EA.hy926 might be attributed to the inhibition of phosphorylation of VEGFR-2. These results suggested that QDAU5 might exhibit anti-angiogenic and anti-cancer potency by inhibiting VEGFR-2 activation.

**Figure 6:**
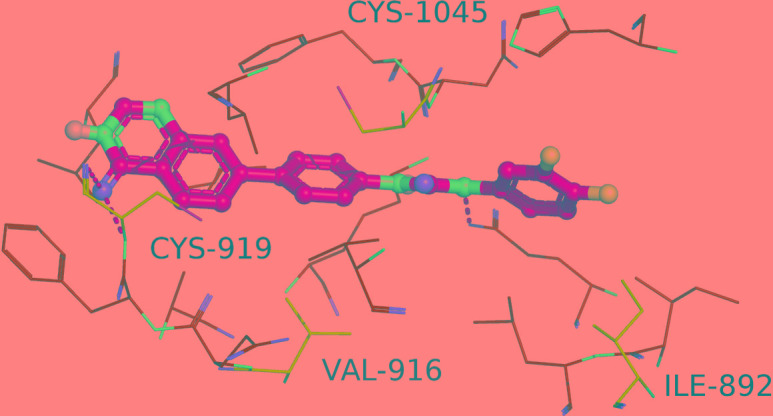
Docked molecule QDAU5 (yellow sticks) and residues within 4Å in the crystal structure of VEGFR-2 Residues that are same or in the same classes are colored in green while different residues are colored in cyan.

To gain a better understanding of its interaction with RTKs, molecular docking was conducted with the crystal structure of VEGFR-2. Besides, the different residues of three proteins were picked out for further analysis (Table 7). As we can see in Figure 6, QDAU-5 forms hydrogen bonding interactions with the side chain of the conserved Glu885 and the backbone of Cys979. For the four different residues, because it is the backbone of Cys979 to interact with the inhibitor, its side chain does not affect much. Second, the closet distances between inhibitor and Ile 892, Val 916 and Cys1045 are 3.5Å, 3.8Å and 2.8Å, respectively, so they don't greatly change the pocket. Our docking result may explain why QDAU5 shows activities on all three proteins.

**Table 7:** Different residues of three RTKs (VEGFR-2, Tie-2, EphB4)

Different residues of three proteins			
Index in VEGFR-2	VEGFR-2	Tie-2	EphB4
892	I	L	F
916	V	I	T
919	C	A	M
1045	C	A	S

## MATERIALS AND METHODS

### Biological evaluation

#### *In vitro* angiogenic RTKs inhibition assays

The *in vitro* RTK inhibition assays against VEGFR-2, TIE-2, and EphB4 of all the title compounds were detected using the ADP-Glo™ kinase assay kit (Promega, Madison) with sorafenib as a positive control. The kinase assay was performed in a reaction mixture of final volume of 10 μL. General procedures are as the following: for VEGFR-2 assays, the tyrosine kinase (0.6 ng/mL) were incubated with substrates (0.2 mg/mL), test title compounds (1.2×10^−4^∼12μM) and ATP (50 μM) in a final buffer of Tris 40 mM, MgCl_2_ 10 mM, BSA 0.1 mg/mL, DTT 1 mM in 384-well plate with the total volume of 5 μL. The plate was incubated at 30°C for 1 h. After the plate was cooled at room temperature for 5 min, 5 μL of ADP-Glo reagent was added into each well to stop the reaction and consume the remaining ADP within 40 min. At the end, 10 μL of kinase detection reagent was added into the well and incubated for 30 min to produce a luminescence signal. As for TIE-2 and EphB4 assays, the kinase (2.4 ng/mL) were incubated with substrates (0.2 mg/mL), tested compounds (1.2 ×10^−4^∼12 μM) and ATP (50 μM) in a final buffer of Tris 40 mM, MgCl_2_ 10 mM, BSA 0.1 mg/mL, DTT 1 mM in 384-well plate with the total volume of 5 μL. The assay plate was incubated at 30°C for 4 h. After the plate was cooled at room temperature for 5 min, 5 μL of ADP-Glo reagent was added into each well to stop the reaction and consume the remaining ADP within 1 h. At the end, 10 μL of kinase detection reagent was added into each well and incubated for 30 min to produce a luminescence signal. The luminescence was read by VICTOR-X multi-label plate reader.

#### *In vitro* RTK selectivity assays [27]

A 50 μL reaction mixture contains 40 mM Tris, pH 7.4, 10 mM MgCl_2_, 0.1 mg/ml BSA, 1 mM DTT, 10 μM ATP, kinase and substrate (0.2 mg/mL Poly(Glu, Tyr)/10 μM ATP). The test compounds were diluted in 10% DMSO and 5 μL of the dilution was added to a 50 μL reaction so that the final concentration of DMSO is 1% in all of reactions. The assay was performed using Kinase-Glo Plus luminescence kinase assay kit. It measures kinase activity by quantitating the amount of ATP remaining in solution following a kinase reaction. The luminescent signal from the assay is correlated with the amount of ATP present and is inversely correlated with the amount of kinase activity. The IC_50_ values were calculated using nonlinear regression with normalized dose−response fit using Prism GraphPad software.

#### Human endothelial cells (EA.hy926) viability assay [28]

The viability of HECs (EA.hy926) was assessed using the cell counting kit-8 (CCK-8, Sigma, USA) assay according to the manufacturer's instruction. In brief, EA-hy926 cells were harvested and plated in a 96-wellplate at the density of 1×10^5^ cells for each well and cultured in DMEM containing 10% FBS in humidified 5% CO_2_ atmosphere. After incubation at 37°C for 48 h, the cells were treated with tested compounds at various concentrations for 24 h. Subsequently, premixed CCK-8 and medium (10 μL) were added into the 96-well plates to monitor cell viability and were incubated at 37°C for 2 h. The number of viable cells was assessed by measurement of absorbance at 450 nm by a microplate reader. The viability rate was calculated as experimental OD value/control OD value.

#### Apoptosis detection assay [29]

EA.hy926 cells (5×10^5^cells/mL) were seeded in six-well plates and treated with QDAU5 at different concentrations (0, 5, 20, and 40 nM) for 48 h. The cells were then harvested by trypsinization and washed twice with cold PBS. After centrifugation and removal of the supernatants, EA.hy926 cells were re-suspended in 400 μL of 1×binding buffer which was then added to 5 μL of annexin V-FITC and incubated at room temperature for 15 min. After adding 10 μL of PI the cells were incubated at room temperature for another 15 min in the dark. The stained cells were analyzed by a flow cytometer (BD Accuri C6).

### Anti-proliferative activity against cancer cell lines

Anti-proliferative activities of QDAU5 were evaluated against fifteen cancer cell lines using 3-(4,5-dimethylthiazol-2-yl)-2,5-diphenyltetrazolium bromide (MTT) method. Exponentially growing cells were harvested and plated in 96-well plates at a concentration of 1×10^4^ cells/well per well, and then were incubated at 37°C for 24 h. The cells were treated with QDAU5 at various concentrations for 48 h. Subsequently, 22 μL fresh MTT (5 mg/mL) was added to each well and incubated at 37°C for 4 h. 150 mL DMSO was added to each well after supernatant was discarded. Absorbance values were determined by a microplate reader (Bio-Rad Instruments) at 490 nm. The IC_50_ values were calculated according to inhibition ratios.

#### *In vivo* xenograft model [30]

Six-week old female BALB/c mice were purchased from Shanghai Laboratory Animal center of the Chinese Academy of Sciences and housed under aseptic and ventilated condition. The mice were inoculated by subcutaneous injection with MCF-7 cancer cells suspended in 5% saline. Tumor volume and weight were measured on alternate days. Body weights were monitored as an indicator of toxicity. When tumor volume exceeds100 mm^3^, mice were randomly divided into treatment and control groups (n=8 per group): QDAU5 and vehicle control (0.5% CMC-Na). Treatment groups were administered orally with QDAU5 daily at doses of 20, 40, and 80 mg/kg, respectively. Treatment started from the next day and continued for 21 days. All mice were killed at the end of the experiment, and subcutaneous tumors were removed and weighted.

### Western blot analysis

The expression and phosphorylation of VEGFR-2 in EA.hy926 cells were assessed using western blot analysis with various antibodies. After treatment with QDAU5 at various concentrations for 48 h, EA.hy926 cells were washed twice with ice-cold PBS (137 mM NaCl, 2.7 mM KCl, 10 mM Na_2_HPO_4_, and 1.8 mM KH_2_PO_4_, pH 7.4) and lysed in SDS sample buffer. Cell lysates containing equal amounts of protein were separated by SDS polyacrylamide gel electrophoresis and transferred to polyvinylidene difluoride membranes. After being blocked in 5% nonfat milk in Tris-buffered saline with 0.1% Tween 20, pH 7.6, the membranes were incubated with the appropriate primary antibodies at 4°C overnight and then exposed to secondary antibodies for 2 h at room temperature. Immunoreactive proteins were visualized using the enhanced chemiluminescence system from Pierce Chemical.

### VEGFR-2/CMC evaluation

The VEGFR-2 cell membrane stationary phase was prepared as previous reported. Aqueous ammonium acetate buffer solution (pH 7.4, 5 mM) was pumped at a flow rate of 0.2 mL/min as the mobile phase after VEGFR-2/CMC column was loaded onto the chromatography system at the temperature of 37°C. The standard solutions of five multi-target RTK inhibitors and QDAU5 were injected to evaluate their affinity with VEGFR-2. The mobile phase was 5.0 mM phosphate burred saline with a 0.6 mL/min flow rate at a column temperature of 37°C. The detected wavelength was 270 nm. The test compound was injected into the VEGFR-2/CMC column and their retention characteristics were identified to evaluate their affinity with VEGFR-2. A direct competitive assay was studied by using QDAU5 as a competitor at different concentrations.

### Molecular docking

The crystal structure (PDBID: 4ASD) of VEGFR-2 was used for docking. The small molecule's 3D structure was optimized with Omega2 [31, 32]. Docking study was conducted using OEDocking module FRED [33, 34] with default parameters on GSU cluster Orion [35] and XSEDE [36].

### General methods for chemistry

All solvents and reagents were purchased from commercial suppliers and purified according to the standard procedure. The reactions except those in aqueous media were carried out by standard techniques for the exclusion of moisture. All the reactions were monitored by thin layer chromatography (TLC) on 0.25-mm silica gel plates (60GF-254) and visualized with UV light. Flash column chromatography was performed using silica gel (300-400 mesh). Melting points were determined using an electrothermal melting point apparatus and were uncorrected. ^1^H-NMR and ^13^C-NMR spectra were recorded on a Bruker Advance AC 400 instrument at 400 MHz and 101 MHz, respectively. Chemical shift (δ) are reported in parts per million (ppm) with TMS as an internal standard and coupling constants (*J*) are expressed in hertz (Hz). High resolution mass spectra (HRMS) analysis was performed on a Shimadzu LCMS-IF-TOF instrument. All the title compounds were determined to be ≥95% pure by HPLC analysis which was carried out on a Shimadzu HPLC instrument.

### General Procedure for the Synthesis of diarylthiourea derivativesDATU 1-10

#### (3-bromo-5-(trifluoromethyl)phenyl) carbamodithioic acid (2)

3-Bromo-5-(trifluoromethyl) aniline (2.5 g, 10.4 mmol), DABC (1.4 g, 12.5 mmol) was dissolved in toluene (40 mL). Then 1.9 mL carbon disulphide was added dropwise to the above mixture. Subsequently, the mixture was reacted at room temperature for 8 h. The product was collected by filtration and dried under vacuum to afford 2 as a slight yellow solid (1.2g, 40%).

#### 1-bromo-3-isothiocyanato-5-(trifluoromethyl)benzene (3)

Compound 2 (0.8 g, 2.5 mmol) was dissolved in anhydrous CH_2_Cl_2_ (20 mL). Then triphosgene (0.88 g, 3 mmol) in anhydrous CH_2_Cl_2_ was added dropwise to the above mixture on the ice-bath, and stirring continued for 2 h at room temperature. After completion of the reaction, the organic layer was washed with water and brine (100 mL×3), and dried over Na_2_SO_4_. After filtration and concentration *in vacuo*, the residue was purified by silica gel flash chromatography to afford 3 (0.50 g, 30.0%).

#### 4-(pyridin-3-yl)aniline (6)

A flask charged with Pd(PPh_3_)_4_ (3.8 g, 3.25 mmol), K_2_CO_3_ (13.5 g, 97.5 mmol), and 4-bromoaniline (9) (5.00 g, 29.30 mmol) and pyridin-3-ylboronic acid (8) (4.0 g, 32.5 mmol) were flushed with nitrogen and suspended in 1,4-dioxane (90 mL) and water (30 mL). The mixture was then refluxed overnight under nitrogen. The hot suspension was filtered and the filtrate distilled by rotary evaporation to remove 1,4-dioxane. Water (50 mL) was added and the product was extracted with AcOEt (30 mL×3), washed with water, and dried over Na_2_SO_4_. After filtration and concentration *in vacuo*, the residue was purified by silica gel flash chromatography (PE/AcOEt = 5:1) affording 3 as white solid (2.8 g, 40%).

#### N-[3-bromo-5-(trifluoromethyl)phenyl]-N’-(4-pyridin-3-ylphenyl)thiourea (DATU1)

The key intermediate 4-(pyridin-3-yl)aniline 6 (0.80 g, 2.84 mmol) was dissolved in anhydrous CH_2_Cl_2_ (10 mL).1-Bromo-3-isothiocyanato-5-(trifluoromethyl)benzene 3 in anhydrous CH_2_Cl_2_ (10 mL) was added dropwise to the above mixture, and stirring on the ice-bath continued for 2 h. After stirring at room temperature for 18 h. The organic layer was washed with water and brine (100 mL×3), and dried over Na_2_SO_4_. After filtration and concentration *in vacuo*, the residue was purified by silica gel flash chromatography to yield DATU1 in 50% yield. HRMS *m/z* calcd for C_19_H_13_BrF_3_N_3_S ([M+H]^+^) 453.2932, found 453.6968. m.p.=197-199°C, ^1^H NMR (400 MHz, DMSO-d6) δ 9.28 (s, 1H), 9.13 (s, 1H), 8.89 (d, *J* = 2.0 Hz, 1H), 8.54 (m, 1H), 8.06 (m, 1H), 7.98 (s, 1H), 7.90 (s, 1H), 7.70 (d, *J* = 8.7 Hz, 2H), 7.62 (d, *J* = 8.7 Hz, 2H), 7.54 (s, 1H), 7.47 (m, 1H).^13^C NMR (101 MHz, DMSO-d6) δ 152.73, 148.42, 147.70, 142.59, 139.77, 135.59, 133.98, 131.53, 131.34, 127.75, 125.02, 124.56, 124.31, 122.83, 122.30, 121.06, 119.60, 113.97.

The title compounds DATU2-10 were prepared from the key intermediate 3 and 6 with a similar procedure as described for compound DATU1.

*N-[4-bromo-2-(trifluoromethoxy)phenyl]-N’-(4-pyridin-3-ylphenyl)thiourea (DATU2)*.

HRMS *m/z* calcd for C_19_H_13_BrF_3_N_3_OS ([M]^+^) 466.9915, found 466.9940. m.p.=144-145°C, ^1^H NMR (400 MHz, DMSO-d6) δ 10.25 (s, 1H), 9.65 (s, 1H), 8.92 (d, *J* = 2.2 Hz, 1H), 8.56 (m, 1.4 Hz, 1H), 8.11-8.07 (m, 1H), 7.76 (s, 1H), 7.74 (m, 2H), 7.70 (s, 1H), 7.66 (d, *J* = 8.4 Hz, 2H), 7.64-7.61 (m, 1H), 7.49 (m, 1H).^13^C NMR (101 MHz, DMSO-d6) δ 180.72, 148.76, 147.90, 143.48, 139.67, 135.43, 134.27, 133.71, 132.13, 131.24, 130.90, 127.46, 124.76, 124.48, 124.36, 121.70, 119.14, 118.57.

*N-[2-bromo-4-(trifluoromethoxy)phenyl]-N’-(4-pyridin-3-ylphenyl)thiourea (DATU3).* HRMS *m/z* calcd for C_19_H_13_BrF_3_N_3_OS ([M]^+^) 466.9915, found 469.9937. m.p.=162-163°C, ^1^H NMR (400 MHz, DMSO-d6) δ 10.26 (s, 1H), 9.61 (s, 1H), 8.92 (d, *J* = 1.8 Hz, 1H), 8.56 (m, 1H), 8.11-8.06 (m, 1H), 7.80 (d, *J* = 2.2 Hz, 1H), 7.76 (d, *J* = 8.7 Hz, 2H), 7.70 (d, *J* = 5.1 Hz, 2H), 7.68 (d, *J* = 5.3 Hz, 1H), 7.51-7.47 (m, 1H), 7.47-7.43 (m, 1H).^13^C NMR (101 MHz, DMSO-d6) δ 180.73, 148.75, 147.89, 146.61, 139.64, 138.05, 135.44, 134.25, 133.65, 131.87, 127.49, 125.78, 124.53, 124.36, 122.57, 121.71, 121.08, 119.16.

*N-[5-bromo-2-(trifluoromethoxy)phenyl]-N’-(4-pyridin-3-ylphenyl)thiourea (DATU4).* HRMS *m/z* calcd for C_19_H_13_BrF_3_N_3_OS ([M]^+^) 466.9915, found 469.9939. m.p.=154-155°C, ^1^H NMR (400 MHz, DMSO-d6) δ 10.34 (s, 1H), 9.70 (s, 1H), 8.92 (d, *J* = 2.2 Hz, 1H), 8.57 (m, 1.4 Hz, 1H), 8.11-8.06 (m, 2H), 7.76 (d, *J* = 8.6 Hz, 2H), 7.67 (d, *J* = 8.6 Hz, 2H), 7.54 (m, 1H), 7.49 (m, 1H), 7.41 (m, 1H).^13^C NMR (101 MHz, DMSO-d6) δ 180.62, 148.77, 147.91, 142.22, 139.57, 135.42, 134.28, 133.97, 133.82, 131.70, 130.03, 127.49, 124.53, 124.36, 123.44, 121.70, 119.36, 119.13.

*N-[2-bromo-5-(trifluoromethoxy)phenyl]-N’-(4-pyridin-3-ylphenyl)thiourea (DATU5).* HRMS *m/z* calcd for C_19_H_13_BrF_3_N_3_OS ([M]^+^) 466.9915, found 469.9935. m.p.=125-126°C, ^1^H NMR (400 MHz, DMSO-d6) δ 10.38 (s, 1H), 9.63 (s, 1H), 8.92 (s, 1H), 8.57 (d, *J* = 3.3 Hz, 1H), 8.10 (d, *J* = 7.9 Hz, 1H), 7.83 (d, *J* = 8.8 Hz, 1H), 7.76 (d, *J* = 8.5 Hz, 2H), 7.70 (s, 2H), 7.68 (s, 1H), 7.49 (m, 1H), 7.25 (d, *J* = 8.2 Hz, 1H).^13^C NMR (101 MHz, DMSO-d6) δ 180.38, 148.78, 147.91, 147.48, 139.93, 139.47, 135.42, 134.32, 134.27, 133.77, 127.53, 124.58, 124.37, 122.80, 121.69, 120.71, 119.87,119.13.

*N-(2,4-dichlorophenyl)-N’-(4-pyridin-3-ylphenyl)thiourea (DATU6).* HRMS *m/z* calcd for C_18_H_13_Cl_2_N_3_S ([M+H]^+^) 374.0207, found 374.0257. m.p.=156-157°C, ^1^H NMR (400 MHz, DMSO-d6) δ 10.23 (s, 1H), 9.63 (s, 1H), 8.91 (d, *J* = 1.9 Hz, 1H), 8.56 (m, 1H), 8.11-8.07 (m, 1H), 7.75 (d, *J* = 8.6 Hz, 2H), 7.71 (d, *J* = 2.3 Hz, 1H), 7.67 (d, *J* = 8.6 Hz, 2H), 7.62 (d, *J* = 8.6 Hz, 1H), 7.49 (m, 1H), 7.45 (m, 1H).^13^C NMR (101 MHz, DMSO-d6) δ 180.68, 148.74, 147.89, 139.69, 136.20, 135.45, 134.25, 133.61, 131.78, 131.46, 131.42, 129.41, 127.86, 127.47, 124.50, 124.36.

*N-(3,4-difluorophenyl)-N’-(4-pyridin-3-ylphenyl)thiourea (DATU7).* HRMS *m/z* calcd for C_18_H_13_F_2_N_3_S ([M+H]^+^) 342.0876, found 342.0851. m.p.=139-140°C, ^1^H NMR (400 MHz, DMSO-d6) δ 10.12 (s, 1H), 10.03 (s, 1H), 8.92 (d, *J* = 1.8 Hz, 1H), 8.56 (m, 1H), 8.11-8.07 (m, 1H), 7.74 (d, *J* = 8.5 Hz, 2H), 7.70 (d, *J* = 2.5 Hz, 1H), 7.62 (d, *J* = 8.6 Hz, 2H), 7.49 (m, 1H), 7.46-7.38 (m, 1H), 7.29-7.24 (m, 1H).^13^C NMR (101 MHz, DMSO-d6) δ 180.18, 148.67, 147.83, 139.79, 136.86, 135.51, 134.31, 133.49, 127.46, 124.48, 124.38, 120.88, 117.55, 117.37, 113.72, 113.52.

*N-(3,4-dichlorophenyl)-N’-(4-pyridin-3-ylphenyl)thiourea (DATU8).* HRMS *m/z* calcd for C_18_H_13_Cl_2_N_3_S ([M+H]^+^) 374.0285, found 374.0258. m.p.=173-175°C, ^1^H NMR (400 MHz, DMSO-d6) δ 10.21 (s, 1H), 10.10 (s, 1H), 8.91 (d, *J* = 1.9 Hz, 1H), 8.57 (m, 1H), 8.12-8.07 (m, 1H), 7.91 (d, *J* = 2.4 Hz, 1H), 7.74 (d, *J* = 8.6 Hz, 2H), 7.62 (d, *J* = 4.1 Hz, 2H), 7.60 (d, *J* = 4.2 Hz, 1H), 7.52-7.49 (m, 1H), 7.47 (m, 1H).^13^C NMR (101 MHz, DMSO-d6) δ 179.97, 148.72, 147.87, 140.21, 139.68, 135.48, 134.30, 133.61, 130.91, 130.68, 127.50, 126.45, 125.27, 124.49, 124.38, 124.05.

*N-[3,5-bis(trifluoromethyl)phenyl]-N’-(4-pyridin-3-ylphenyl)thiourea (DATU9).* HRMS *m/z* calcd for C_20_H_13_F_6_N_3_S ([M+H]^+^) 442.0813, found 442.0791. m.p.=157-158°C, ^1^H NMR (400 MHz, DMSO-d6) δ 10.48 (s, 1H), 10.35 (s, 1H), 8.92 (d, *J* = 1.9 Hz, 1H), 8.61-8.55 (m, 1H), 8.28 (s, 2H), 8.10 (d, *J* = 8.0 Hz, 1H), 7.83 (s, 1H), 7.77 (d, *J* = 8.5 Hz, 2H), 7.61 (d, *J* = 8.5 Hz, 2H), 7.50 (m, 1H).^13^C NMR (101 MHz, DMSO-d6) δ 180.19, 148.82, 147.93, 142.30, 139.23, 135.41, 134.31, 134.05, 130.64, 130.31, 127.71, 127.20, 125.07, 124.75, 124.38, 124.04, 122.36, 117.43.

*N-1,3-benzodioxol-5-yl-N’-(4-pyridin-3-ylphenyl)thiourea (DATU10).* HRMS *m/z* calcd for C_19_H_15_N_3_O_2_S ([M+H]^+^) 350.0963, found 350.0931. m.p.=147-148°C, ^1^H NMR (400 MHz, DMSO-d6) δ 9.84 (s, 1H), 9.79 (s, 1H), 8.91 (d, *J* = 1.5 Hz, 1H), 8.60-8.53 (m, 1H), 8.07 (d, *J* = 8.0 Hz, 1H), 7.71 (d, *J* = 8.5 Hz, 2H), 7.63 (d, *J* = 8.5 Hz, 2H), 7.48 (m, 1H), 7.12 (d, *J* = 1.4 Hz, 1H), 6.90 (d, *J* = 8.3 Hz, 1H), 6.82 (m, 1H), 6.04 (s, 2H).^13^C NMR (101 MHz, DMSO-d6) δ 180.21, 148.66, 147.86, 147.38, 144.98, 140.17, 135.54, 134.19, 133.69, 133.15, 127.29, 124.42, 124.35, 118.11, 108.29, 106.95, 101.72.

General Procedure for the Synthesis of quinazolinone diaryl urea derivatives QDAU1-10.

*7-Bromoquinazolin-4(3H)-one (8).* Around bottom two flask charged with 2-amino-5-bromobenzoicacid 7 (5.00 g, 23.14 mmol) was flushed with nitrogen and suspended in HCONH_2_ (80 mL, 2.01 mmol), the mixture was stirred at 150°C for 1.5 h by atmospheric microwave heating. The product was extracted with AcOEt (50 mL ×3), washed with water, and dried over Na_2_SO_4_. After filtration and concentration *in vacuo*, the residue was purified by silica gel flash chromatography (PE/AcOEt=5:1) affording 8 (1.46 g, 41.24%) as white solid. mp: 259∼261°C.

*1-(4-(4,4,5,5-tetramethyl-1,3,2-dioxaborolan-2-yl)phenyl)-3-(3-(trifluoromethyl)phenyl)urea(10).* Triphosgene (0.80 g, 2.74 mmol) was dissolved in anhydrous CH_2_Cl_2_ (20 mL) and the mixture was stirred on the ice-bath for 5 min. A solution of 3-(trifluoromethyl)aniline (1.10 g, 6.85 mmol) in anhydrous CH_2_Cl_2_ was added dropwise to the above mixture and stirring was continued for 15 min. Then triethanolamine (1.15 mL,8.22 mmol) diluted with CH_2_Cl_2_ (10 mL) was then added onto the mixture. Stirring was continued for 15 min, a solution of triethanolamine (1.15 mL, 8.22 mmol) and 4-(4,4,5,5-tetramethyl-1,3,2-dioxaborolan-2-yl)aniline (1.50 g, 6.85 mmol) in anhydrous CH_2_Cl_2_ (10 mL) was added and continued stirring for 20 min. Subsequently, the ice bath was removed, and the mixture was reacted at room temperature overnight. After completion of the action, the reaction was quenched with dilute NaHCO_3_. The organic layer was washed and dried overNa_2_SO_4_. After filtration and concentration *in vacuo*, the residue was purified by column chromatography (PE/AcOEt=7:1) affording 10 as white solid (1.77 g, 63.67%).

*1-(4-(4-oxo-3,4-dihydroquinazolin-7-yl)phenyl)-3-(3-(trifluoromethyl)phenyl)urea (QDAU1).* A flask charged with Pd(PPh_3_)_4_ (0.39 g, 0.34 mmol), potassium carbonate (1.43 g, 10.35 mmol), and the key intermediate 10 (1.40 g, 3.45 mmol) and 8 (0.78 g, 3.45 mmol) were flushed with nitrogen and suspended in 1,4-dioxane (90 mL) and water (30 mL). The mixture was then refluxed overnight under nitrogen. The hot suspension was filtered and the filtrate distilled by rotary evaporation to remove 1,4-dioxane. Water (50 mL) was added and the product was extracted with AcOEt (30 mL×3), washed with water, and dried over Na_2_SO_4_. After filtration and concentration *in vacuo*, the residue was purified by silica gel flash chromatography (PE/AcOEt=2:1) affording QDAU1 (0.21g) as white solid (yield 14.4%), HRMS *m/z* calcd for C_22_H_15_F_3_N_4_O_2_ ([M]^+^) 424.1147, found 424.1158. m.p. >300°C, ^1^H NMR (400 MHz, DMSO-d6) δ 12.25 (s, 1H), 9.15 (s, 1H), 9.05 (s, 1H), 8.17 (d, J = 8.3 Hz, 1H), 8.13 (s, 1H), 8.05 (s, 1H), 7.91 (d, J = 1.5 Hz, 1H), 7.84 (m, J = 8.4, 1.7 Hz, 1H), 7.80 (d, J = 8.7 Hz, 2H), 7.64 (d, J = 8.8 Hz, 2H), 7.60 (s, 1H), 7.54 (t, J = 7.9 Hz, 1H), 7.34 (d, J = 7.6 Hz, 1H).^13^C NMR (101 MHz, DMSO-d6) δ 161.03, 152.89, 149.83, 146.26, 145.91, 140.95, 140.50, 132.55, 130.43, 128.16, 127.00, 125.34, 124.31, 122.40, 121.46, 119.26, 66.81.

The title compounds QDAU2 −10 were prepared from the key intermediate 8 and 10 with a similar procedure as described for compound QDAU1.

*1-(3-chlorophenyl)-3-(4-(4-oxo-3,4-dihydroquinazolin-7-yl)phenyl)urea (QDAU2).* White solid (yield 6.7%), HRMS *m/z* calcd for C_21_H_15_ClN_4_O_2_ ([M+H]^+^) 391.0884, found 391.0923. m.p. >300°C, ^1^H NMR (400 MHz, DMSO-d6) δ 12.25 (s, 1H), 8.99 (s, 2H), 8.16 (d, J = 7.8 Hz, 1H), 8.13 (s, 1H), 7.87 (d, J = 22.9 Hz, 2H), 7.76 (d, J = 16.8 Hz, 2H), 7.63 (s, 2H), 7.31 (s, 2H), 7.04 (s, 1H). ^13^C NMR (101 MHz, DMSO-d6) δ 161.03, 152.76, 149.83, 146.26, 145.92, 141.63, 140.55, 133.67, 132.48, 130.91, 128.17, 126.99, 125.33, 124.30, 122.05, 121.45, 119.16, 118.09, 117.20.

*1-(3-fluorophenyl)-3-(4-(4-oxo-3,4-dihydroquinazolin-7-yl)phenyl)ure (QDAU3).* White solid (yield 24.6%), HRMS *m/z* calcd for C_21_H_15_FN_4_O_2_ ([M+H]^+^) 375.1179, found 375.1217. m.p. >300°C, ^1^H NMR (400 MHz, DMSO-d6) δ 12.25 (s, 1H), 9.00 (s, 1H), 8.98 (s, 1H), 8.16 (d, J = 8.3 Hz, 1H), 8.13 (s, 1H), 7.90 (s, 1H), 7.83 (d, J = 8.4 Hz, 1H), 7.79 (d, J = 8.5 Hz, 2H), 7.62 (d, J = 8.6 Hz, 2H), 7.52 (d, J = 11.9 Hz, 1H), 7.32 (m, J = 15.3, 7.9 Hz, 1H), 7.15 (d, J = 8.0 Hz, 1H), 6.81 (m, J = 11.6, 5.1 Hz, 1H). ^13^C NMR (101 MHz, DMSO-d6) δ 161.68, 161.03, 152.75, 149.83, 146.25, 145.92, 140.56, 132.47, 130.87, 130.77, 128.17, 126.99, 125.32, 124.30, 121.45, 119.14, 114.46, 108.84, 105.54, 105.27.

*1-(2-fluorophenyl)-3-(4-(4-oxo-3,4-dihydroquinazolin-7-yl)phenyl)urea (QDAU4).* White solid (yield 22.5%), HRMS *m/z* calcd for C_21_H_15_FN_4_O_2_ ([M+H]^+^) 375.1179, found 375.1222. m.p. >300°C, ^1^H NMR (400 MHz, DMSO-d6) δ 9.81 (s, 1H), 9.17 (s, 1H), 8.16 (t, J = 4.1 Hz, 2H), 8.15 – 8.10 (m, 1H), 7.86 (d, J = 1.2 Hz, 1H), 7.79 (s, 1H), 7.77 (s, 2H), 7.67 (d, J = 8.6 Hz, 2H), 7.25 (m, J = 10.9, 8.8 Hz, 1H), 7.16 (t, J = 7.7 Hz, 1H), 7.04 (m, J = 9.2, 6.5, 1.5 Hz, 1H). ^13^C NMR (101 MHz, DMSO-d6) δ 163.56, 154.06, 152.85, 151.66, 150.38, 148.93, 145.24, 140.69, 132.64, 128.10, 126.98, 124.95, 124.92, 124.56, 123.95, 123.23, 123.15, 121.71, 121.50, 118.94, 115.63, 115.44.

*1-(3,4-difluorophenyl)-3-(4-(4-oxo-3,4-dihydroquinazolin-7-yl)phenyl)urea (QDAU5).* White solid (yield 27.2%), HRMS *m/z* calcd for C_21_H_14_F_2_N_4_O_2_ ([M+H]^+^) 393.1085, found 393.1123. m.p. >300°C, ^1^H NMR (400 MHz, DMSO-d6) δ 12.25 (s, 1H), 8.98 (s, 2H), 8.16 (d, J = 8.3 Hz, 1H), 8.12 (s, 1H), 7.90 (d, J = 1.1 Hz, 1H), 7.86 – 7.81 (m, 1H), 7.78 (d, J = 8.6 Hz, 2H), 7.70 (m, J = 13.3, 7.4, 2.4 Hz, 1H), 7.61 (d, J = 8.7 Hz, 2H), 7.36 (m, J = 19.5, 9.3 Hz, 1H), 7.19 – 7.12 (m, 1H). ^13^C NMR (101 MHz, DMSO-d6) δ 161.03, 152.83, 149.83, 146.24, 145.91, 143.85, 143.72, 140.54, 137.24, 137.14, 132.49, 128.15, 126.98, 125.31, 124.29, 121.45, 119.18, 117.96, 117.78, 114.91, 107.82, 107.61.

*1-(4-fluorophenyl)-3-(4-(4-oxo-3,4-dihydroquinazolin-7-yl)phenyl)urea (QDAU6).* White solid (yield 19.1%), HRMS *m/z* calcd for C_21_H_15_FN_4_O_2_ ([M+H]^+^) 375.1179, found 375.1225. m.p. >300°C, ^1^H NMR (400 MHz, DMSO-d6) δ 9.85 (s, 1H), 9.77 (s, 1H), 8.19 (s, 1H), 8.17 (d, J = 8.6 Hz, 1H), 7.86 (d, J = 1.4 Hz, 1H), 7.81 – 7.78 (m, 1H), 7.76 (d, J = 3.1 Hz, 2H), 7.68 (d, J = 8.7 Hz, 2H), 7.60 – 7.54 (m, 2H), 7.14 (t, J = 8.9 Hz, 2H). ^13^C NMR (101 MHz, DMSO-d6) δ 158.90, 156.54, 153.34, 150.45, 149.15, 145.29, 141.13, 136.87, 132.32, 127.99, 126.95, 124.53, 123.87, 121.40, 120.47, 120.40, 119.01, 115.80, 115.58.

*1-(4-(4-oxo-3,4-dihydroquinazolin-7-yl)phenyl)-3-(4-(trifluoromethoxy)phenyl)urea (QDAU7).* White solid (yield 14.3%), HRMS *m/z* calcd for C_22_H_15_F_3_N_4_O_3_ ([M+H]^+^) 441.1096, found 441.1138. m.p. >300°C, ^1^H NMR (400 MHz, DMSO-d6) δ 12.27 (s, 1H), 9.47 (s, 1H), 9.44 (s, 1H), 8.16 (d, J = 8.3 Hz, 1H), 8.13 (s, 1H), 7.90 (s, 1H), 7.83 (d, J = 8.3 Hz, 1H), 7.79 (d, J = 8.5 Hz, 2H), 7.63 (d, J = 8.6 Hz, 2H), 7.59 (d, J = 8.9 Hz, 2H), 7.30 (d, J = 8.4 Hz, 2H). ^13^C NMR (101 MHz, DMSO-d6) δ 161.04, 152.98, 149.84, 146.25, 145.96, 143.02, 140.80, 139.51, 132.24, 128.16, 126.98, 125.30, 124.24, 122.25, 121.40, 119.69, 119.40, 118.89.

*1-(3-isopropylphenyl)-3-(4-(4-oxo-3,4-dihydroquinazolin-7-yl)phenyl)urea (QDAU8).* White solid (yield 26.7%), HRMS *m/z* calcd for C_24_H_22_N_4_O_2_ ([M+H]^+^) 399.1743, found 399.1780. m.p. >300°C, ^1^H NMR (400 MHz, DMSO-d6) δ 12.25 (s, 1H), 8.87 (s, 1H), 8.71 (s, 1H), 8.17 (d, J = 8.3 Hz, 1H), 8.13 (s, 1H), 7.90 (s, 1H), 7.86 – 7.81 (m, 1H), 7.78 (d, J = 8.6 Hz, 2H), 7.62 (d, J = 8.7 Hz, 2H), 7.37 (s, 1H), 7.28 (d, J = 8.6 Hz, 1H), 7.20 (t, J = 7.8 Hz, 1H), 6.87 (d, J = 7.5 Hz, 1H), 2.85 (m, J = 13.7, 6.8 Hz, 1H), 1.20 (d, J = 6.9 Hz, 6H). ^13^C NMR (101 MHz, DMSO-d6) δ 161.03, 152.90, 149.84, 149.52, 146.24, 145.97, 140.90, 139.99, 132.15, 129.17, 128.13, 126.99, 125.29, 124.23, 121.40, 120.52, 118.95, 116.72, 116.38, 33.97, 24.35.

*1-(4-chloro-3-(trifluoromethyl)phenyl)-3-(4-(4-oxo-3,4-dihydroquinazolin-7-yl)phenyl)urea (QDAU9).* White solid (yield 12.7%), HRMS *m/z* calcd for C_22_H_14_ClF_3_N_4_O_2_ ([M+H]^+^) 459.0757, found 459.0786. m.p. >300°C,^1^H NMR (400 MHz, DMSO-d6) δ 12.26 (s, 1H), 9.64 (s, 1H), 9.39 (s, 1H), 8.16 (d, J = 8.4 Hz, 1H), 8.13 (s, 2H), 7.90 (s, 1H), 7.83 (d, J = 8.4 Hz, 1H), 7.79 (d, J = 8.5 Hz, 2H), 7.65 (d, J = 4.1 Hz, 2H), 7.63 (d, J = 6.5 Hz, 2H). ^13^C NMR (101 MHz, DMSO-d6) δ 161.03, 152.87, 149.83, 146.26, 145.89, 140.41, 139.81, 132.51, 128.17, 126.98, 125.32, 124.31, 123.38, 122.73, 121.46, 119.20, 66.81.

*1-(4-(4-oxo-3,4-dihydroquinazolin-7-yl)phenyl)-3-(4-(trifluoromethyl)phenyl)urea (QDAU10).* White solid (yield 15.1%), HRMS *m/z* calcd for C_22_H_15_F_3_N_4_O_2_ ([M+H]^+^) 425.1147, found 425.1181. m.p. >300°C, ^1^H NMR (400 MHz, DMSO-d6) δ 12.25 (s, 1H), 9.20 (s, 1H), 9.04 (s, 1H), 8.17 (d, J = 8.3 Hz, 1H), 8.13 (s, 1H), 7.90 (s, 1H), 7.82 (d, J = 9.1 Hz, 1H), 7.79 (d, J = 8.5 Hz, 2H), 7.70 (d, J = 8.6 Hz, 2H), 7.64 (d, J = 8.1 Hz, 3H), 7.47 (d, J = 8.1 Hz, 1H). ^13^C NMR (101 MHz, DMSO-d6) δ 161.04, 152.66, 149.82, 146.24, 145.89, 143.82, 140.43, 132.63, 128.17, 126.99, 126.54, 125.31, 124.32, 123.67, 122.49, 122.17, 121.47, 119.22, 118.39.

## CONCLUSIONS

In summary, two classes of twenty multi-target RTKIs have been developed with promising activity on angiogenic and oncogenic VEGFR-2, Tie-2, and EphB4. Since all three RTKs play essential roles in both angiogenesis and tumorigenesis. These multiple RTKIs with a ‘triplet’ inhibition profile might have a major advantage of overcoming the compensatory feedback that characterizes single-target drugs. *In vitro* RTKs inhibition assay and molecular docking revealed that these compounds could suppress VEGFR-2, Tie-2, and EphB4 kinase activity through preferential binding at the ATP-binding site. Biological evaluation using different models led to the discovery of a biphenyl-aryl urea bearing a quinazolin-4(3*H*)-one moiety (QDAU5), which displayed both anti-angiogenic and anticancer activities. Moreover, quinazolin-4(3*H*)-one has been identified as an excellent hinge binding group for multi-target inhibitors of VEGFR-2, Tie-2, and EphB4. Further detailed mechanism of action study and extensive pharmacokinetics evaluation of QDAU5 are in progress and will be reported in due course.

## SUPPLEMENTARY MATERIALS



## Abbreviations

Ang: angiopoietin
DFG: aspartate-phenylalanine-glycine
EC: epithelial cell
EGFR: epidermal growth factor receptor
Eph: erythropoietin-producing hepatoma
FGFR: fibroblast growth factor receptor
HATU: [bis(dimethylamino)methylene]-1*H*-1,2,3-triazolo [4,5-b]pyridinium 3-oxide hexafluorophosphate
HBG: hinge binding group
HECs: Human endothelial cell
KDR: kinase insert domain receptor
RTK: receptor tyrosine kinase
RTKI: receptor tyrosine kinase inhibitor
Tie-2: tyrosine kinase with Ig and epidermal growth factor homology domain-2
VEGFR-2: vascular endothelial growth factor receptor-2.
