# Supplementary material for: Discovery and evaluation of triple inhibitors of VEGFR-2, TIE-2 and EphB4 as anti-angiogenic and anti-cancer agents
Source: Oncotarget. 2017 Aug 8;8(62):104745–60. doi: 10.18632/oncotarget.20065 (PMC5739597; doi:10.18632/oncotarget.20065)
Supplement: Supplementary file 1 [file oncotarget-08-104745-s001.pdf]

Discovery and evaluation of triple inhibitors of VEGFR-2, TIE-2 and EphB4 as anti-angiogenic and anti-cancer agents

SUPPLEMENTARY MATERIALS

Contents of Supplemental Data

(1) ^{1}H-NMR Spectra, and ^{13}C-NMR Spectra of title compounds

(2) High resolution mass spectrometry (HRMS) of title compounds

(3) HPLC analysis chromatogram of all the title compounds

***N*-[3-bromo-5-(trifluoromethyl)phenyl]-*N'*-(4-pyridin-3-ylphenyl)thiourea (DATU1)**

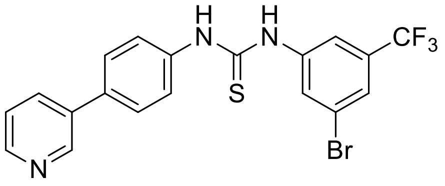

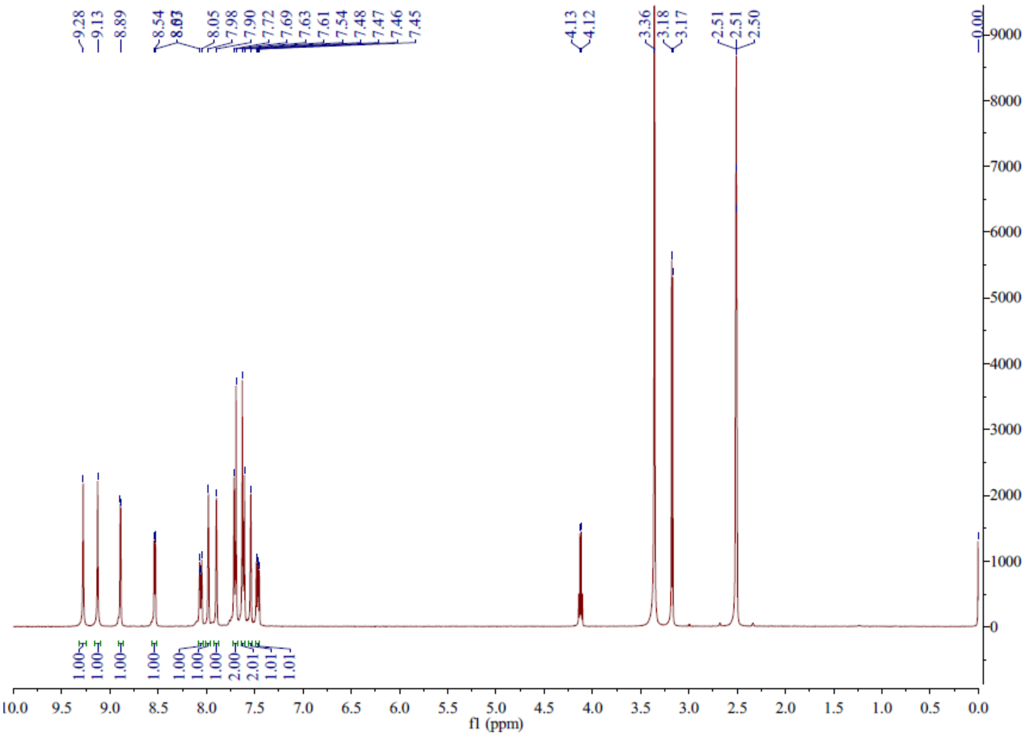

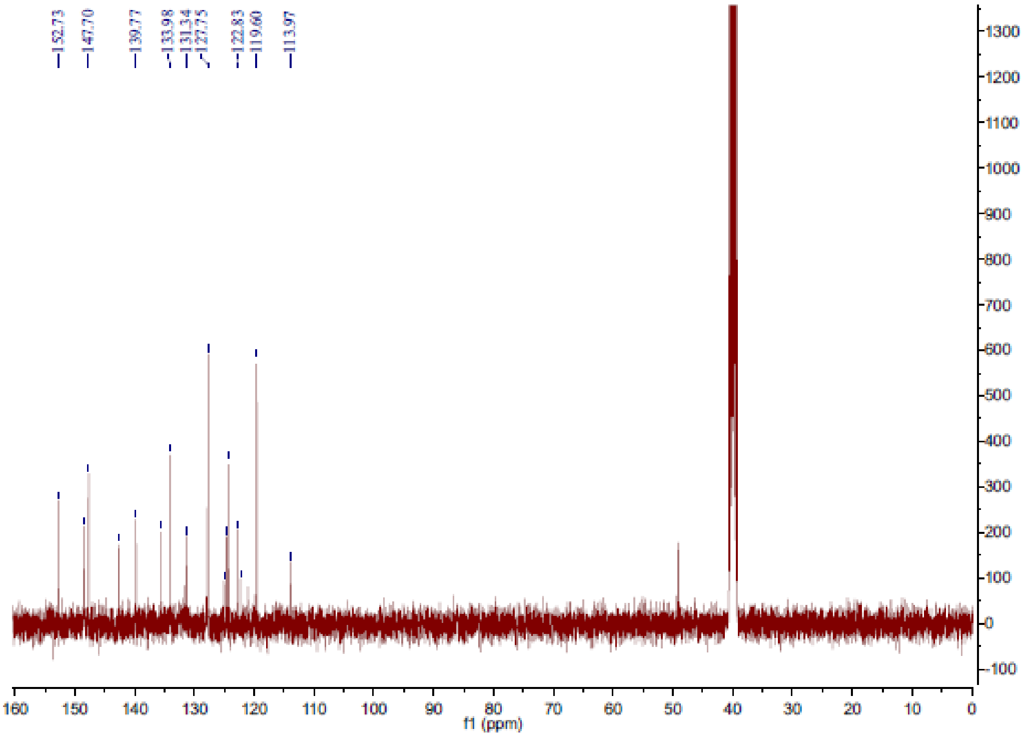

_N_-[4-bromo-2-(trifluoromethoxy)phenyl]-_N′_-(4-pyridin-3-ylphenyl)thiourea (DATU2)

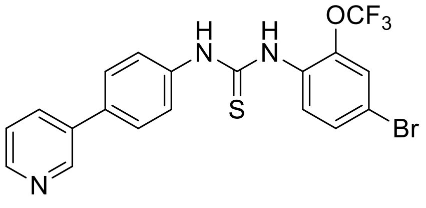

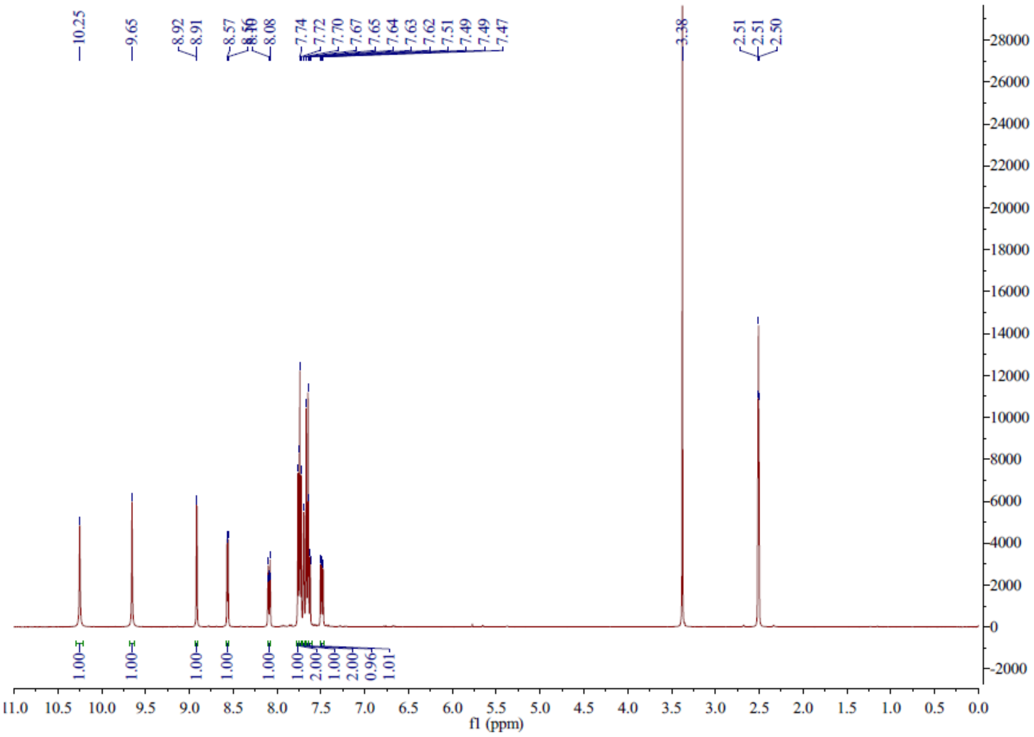

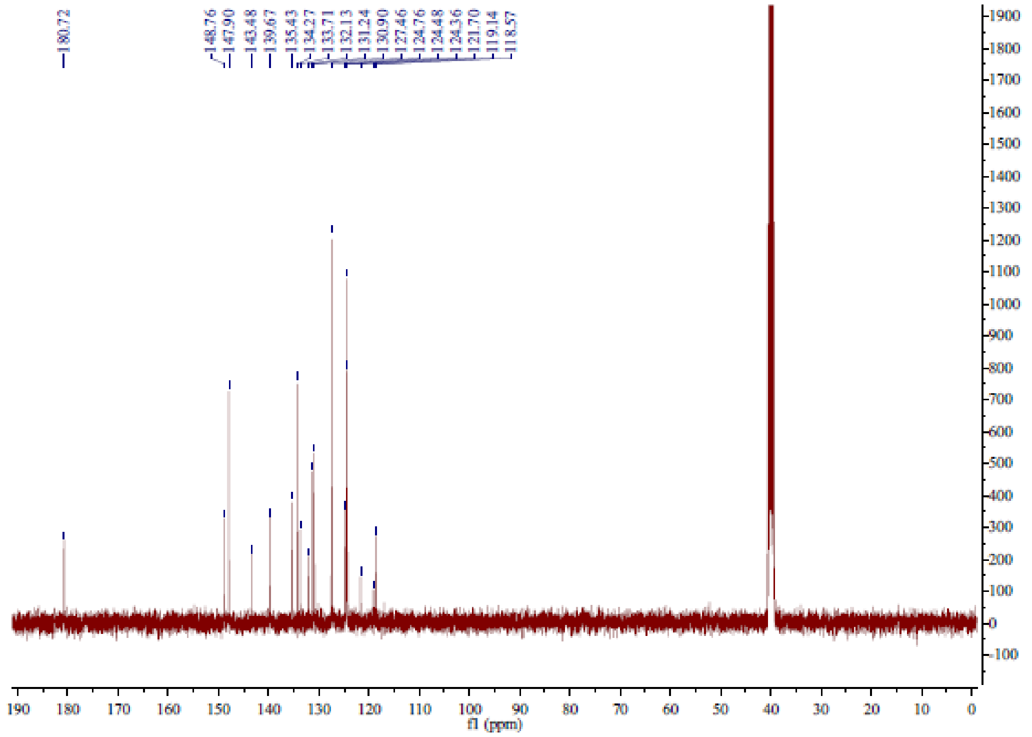

***N*-[2-bromo-4-(trifluoromethoxy)phenyl]-*N'*-(4-pyridin-3-ylphenyl)thiourea (DATU3)**

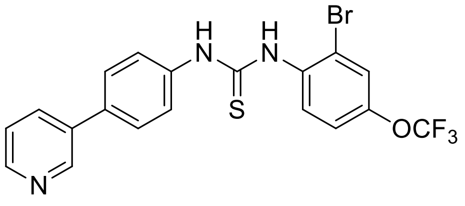

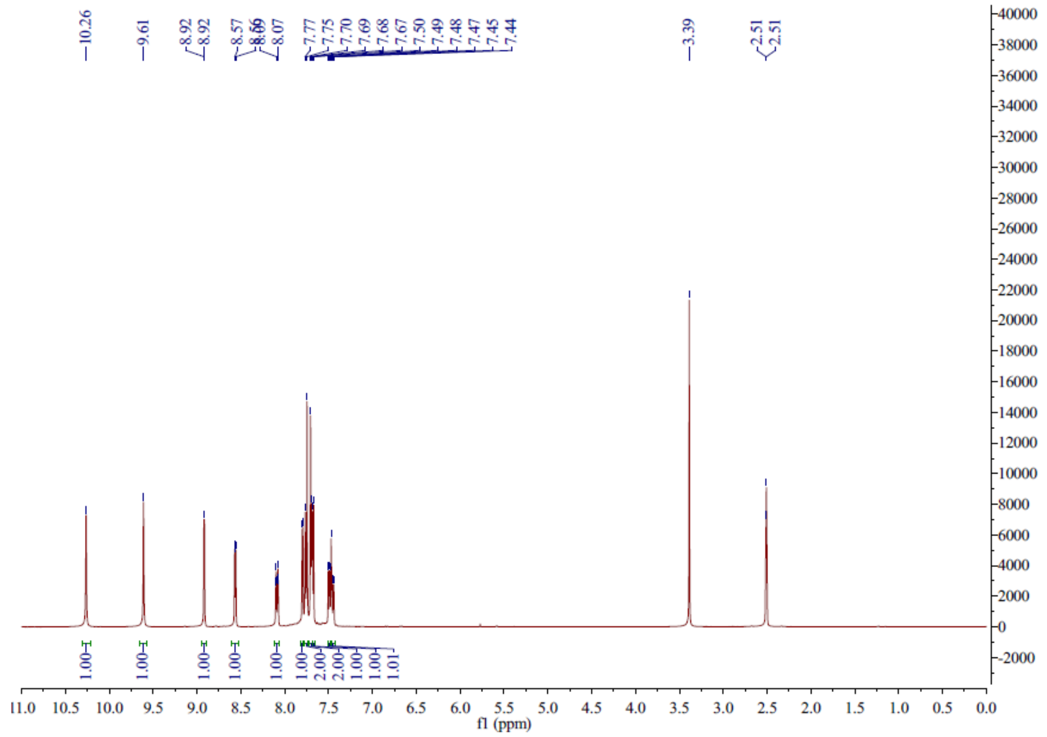

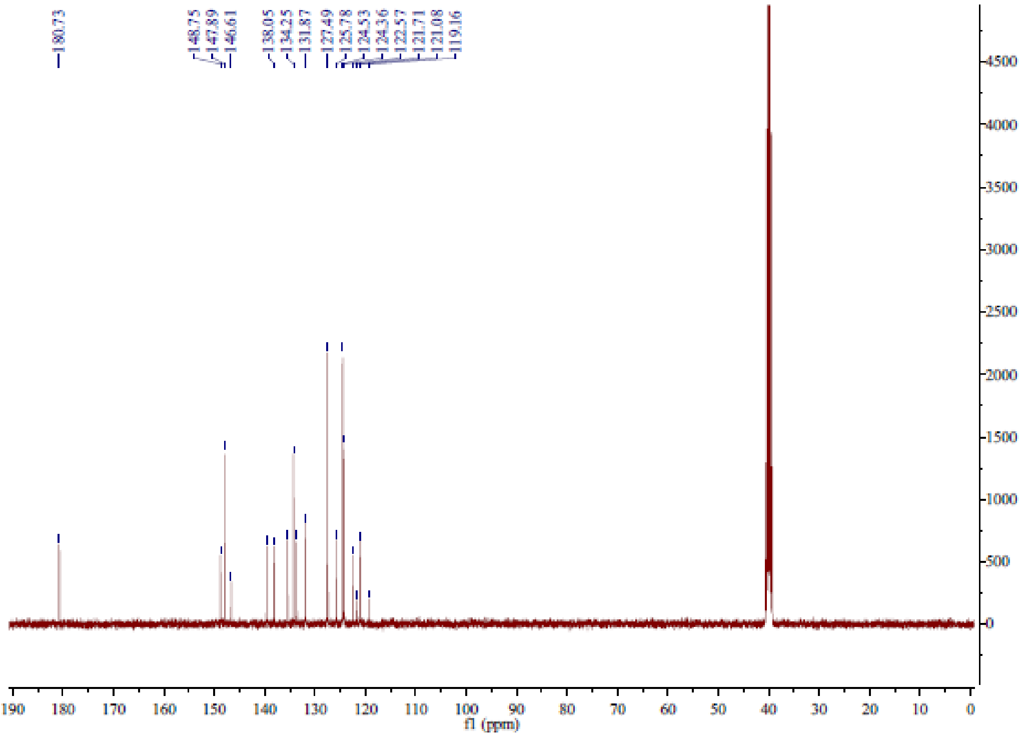

***N*-[5-bromo-2-(trifluoromethoxy)phenyl]-*N′*-(4-pyridin-3-ylphenyl)thiourea (DATU4)**

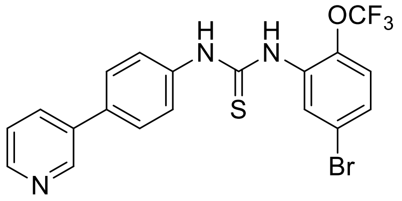

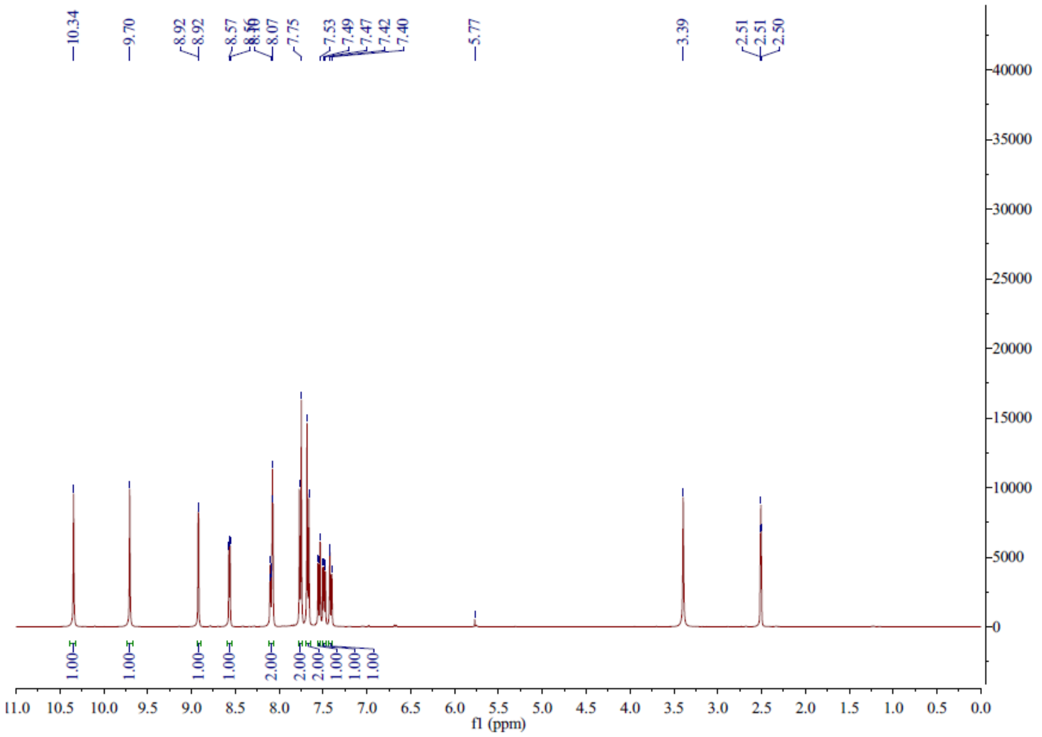

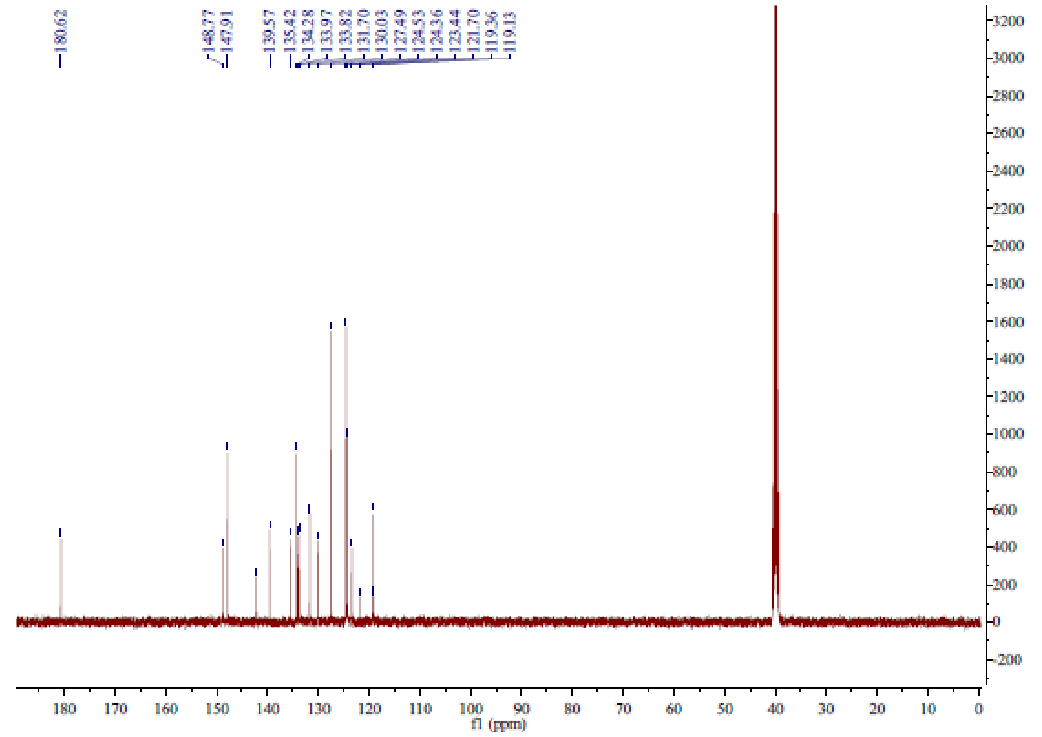

N-[2-bromo-5-(trifluoromethoxy)phenyl]-N′-(4-pyridin-3-ylphenyl)thiourea (DATU5)

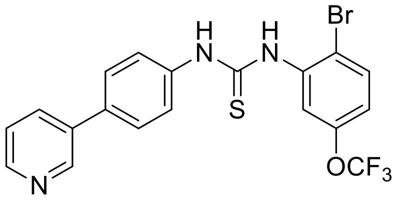

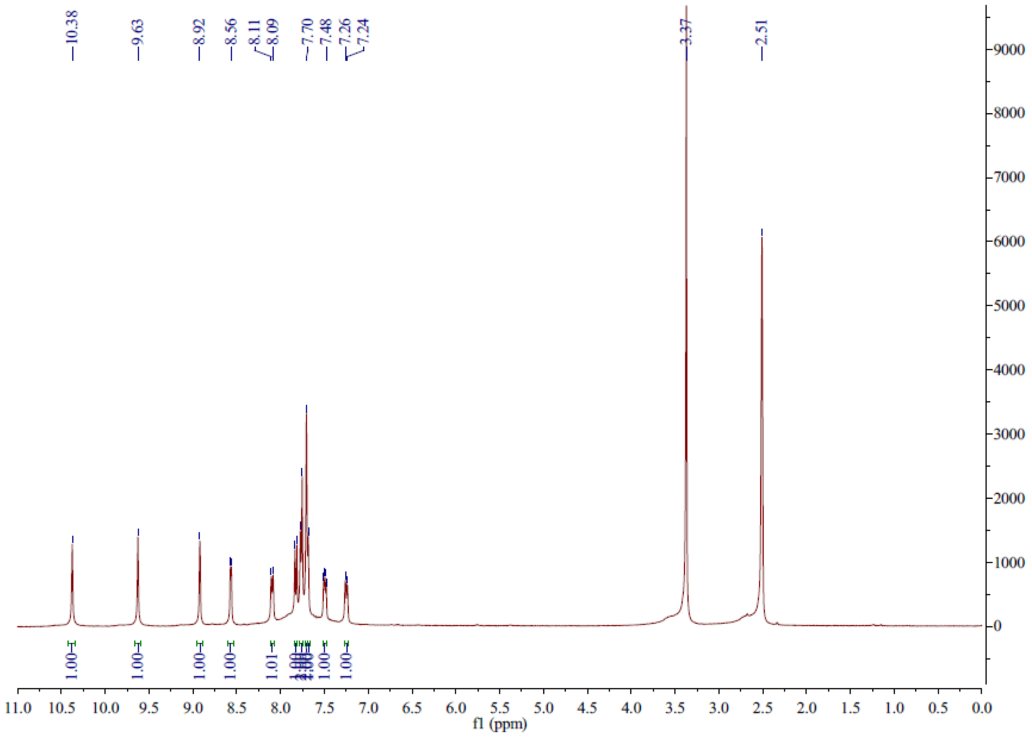

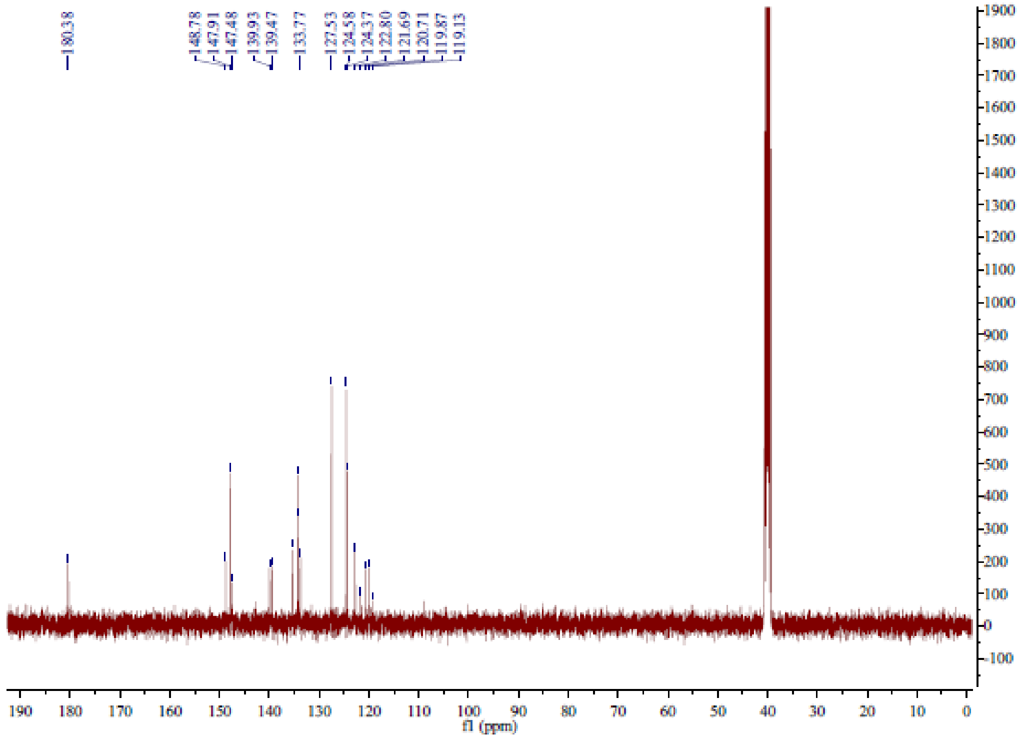

N-(2,4-dichlorophenyl)-N′-(4-pyridin-3-ylphenyl)thiourea (DATU6)

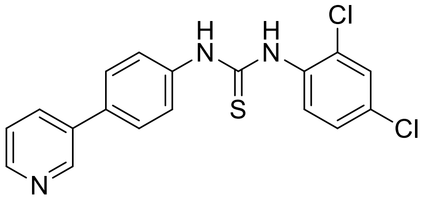

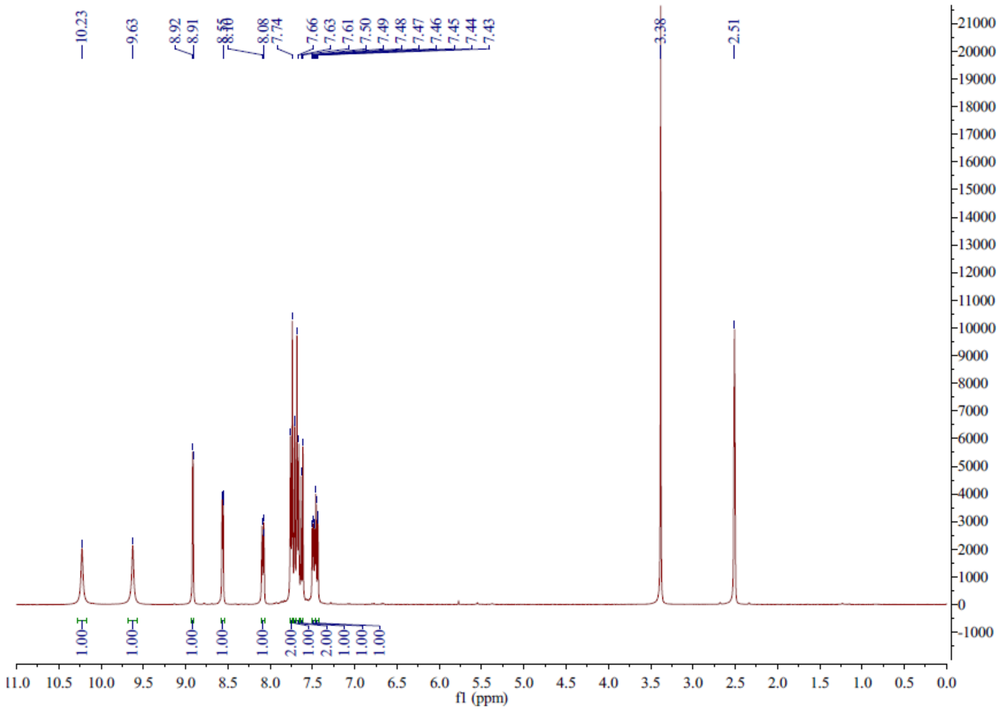

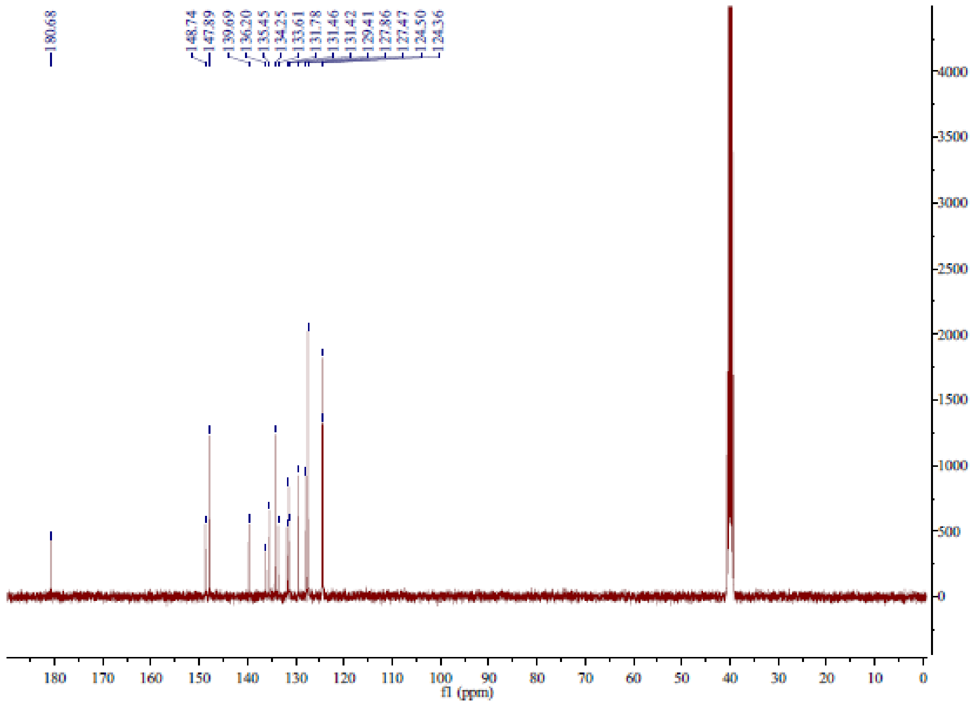

_N_-(3,4-difluorophenyl)-_N'_-(4-pyridin-3-ylphenyl)thiourea (DATU7)

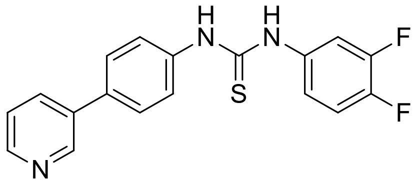

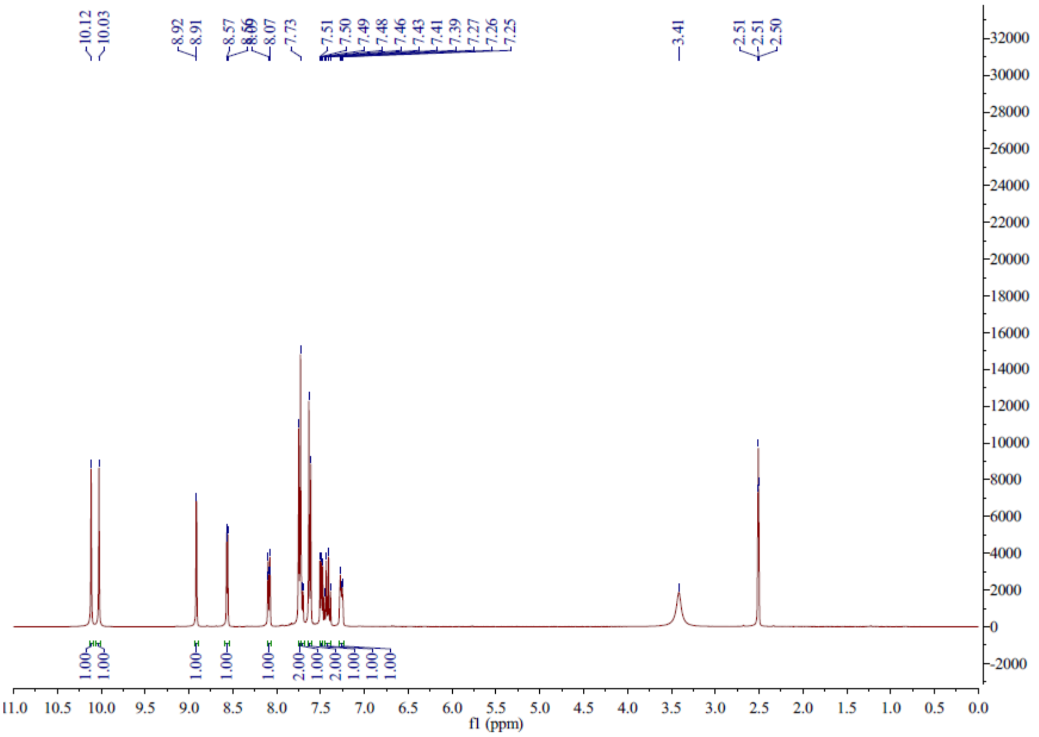

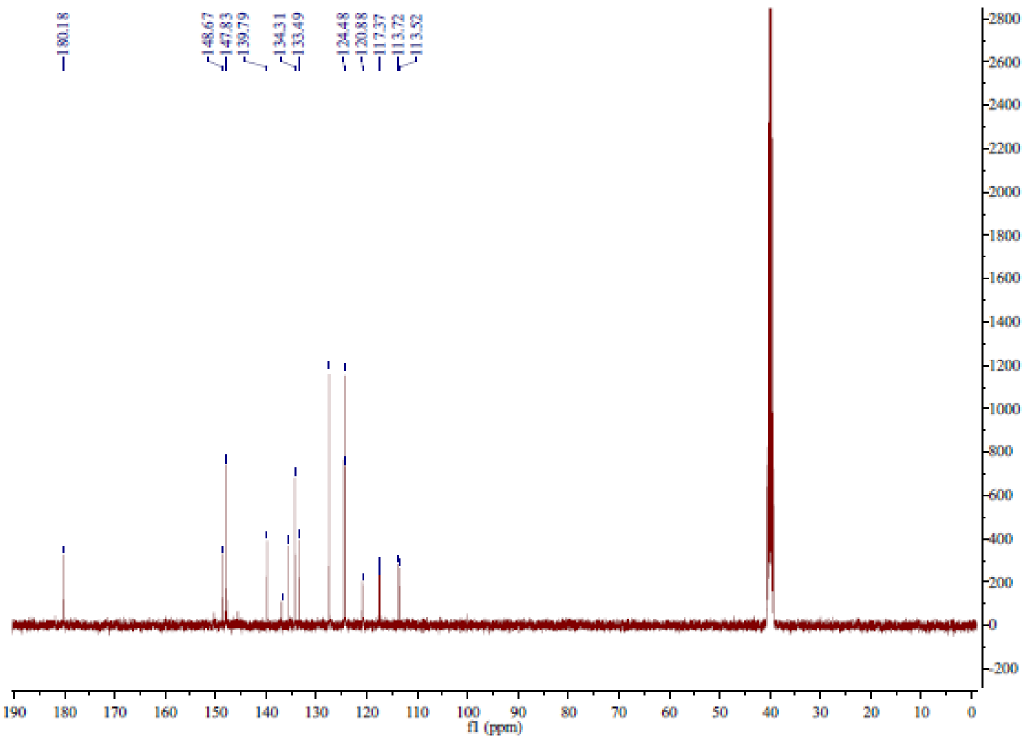

N-(3,4-dichlorophenyl)-N′-(4-pyridin-3-ylphenyl)thiourea (DATU8)

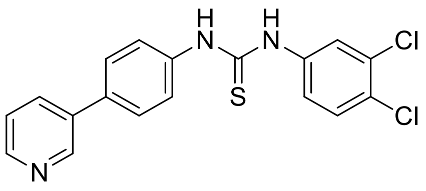

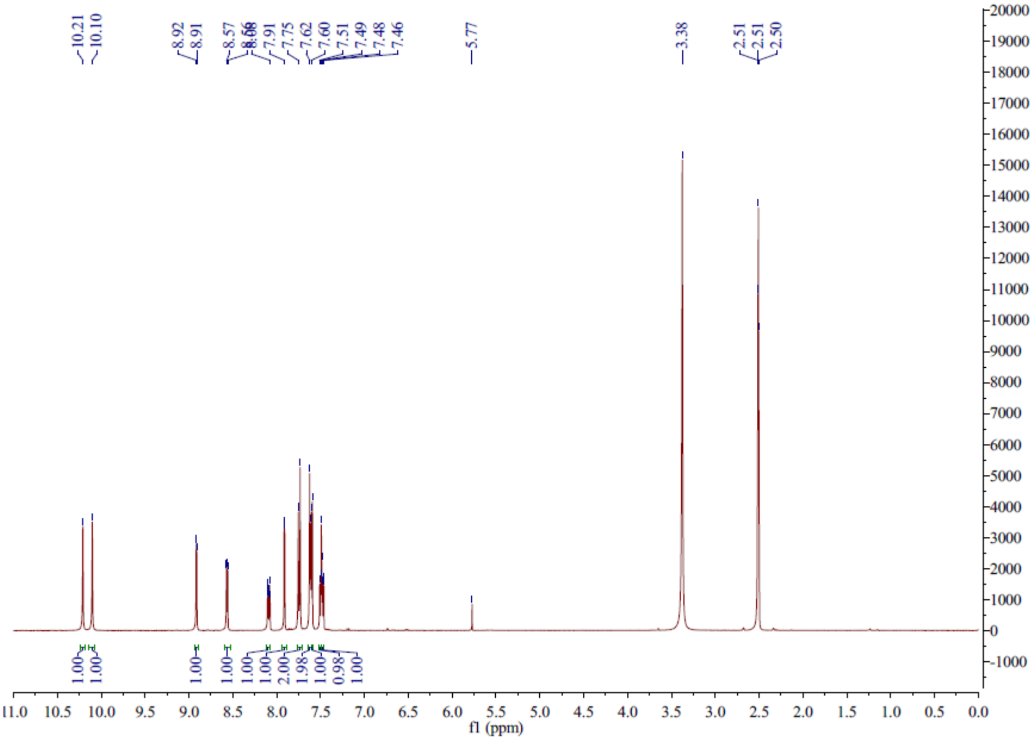

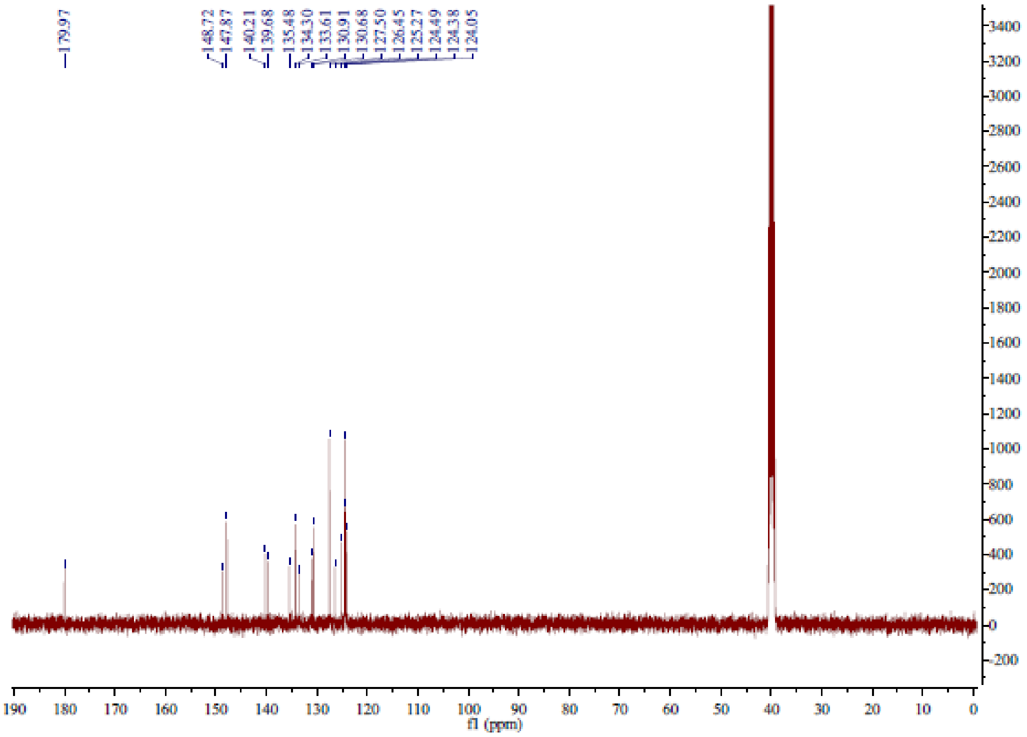

***N*-[3,5-bis(trifluoromethyl)phenyl]-*N'*-(4-pyridin-3-ylphenyl)thiourea (DATU9)**

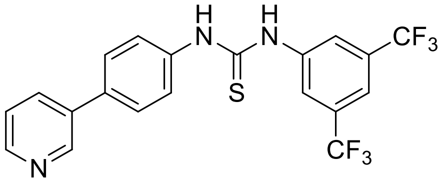

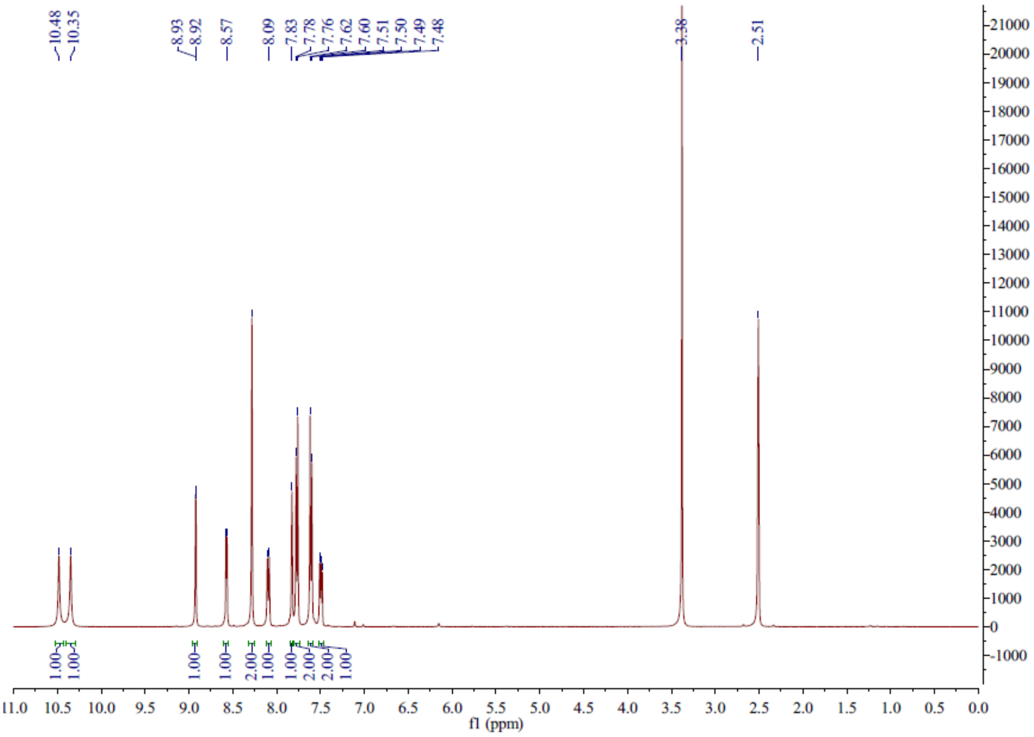

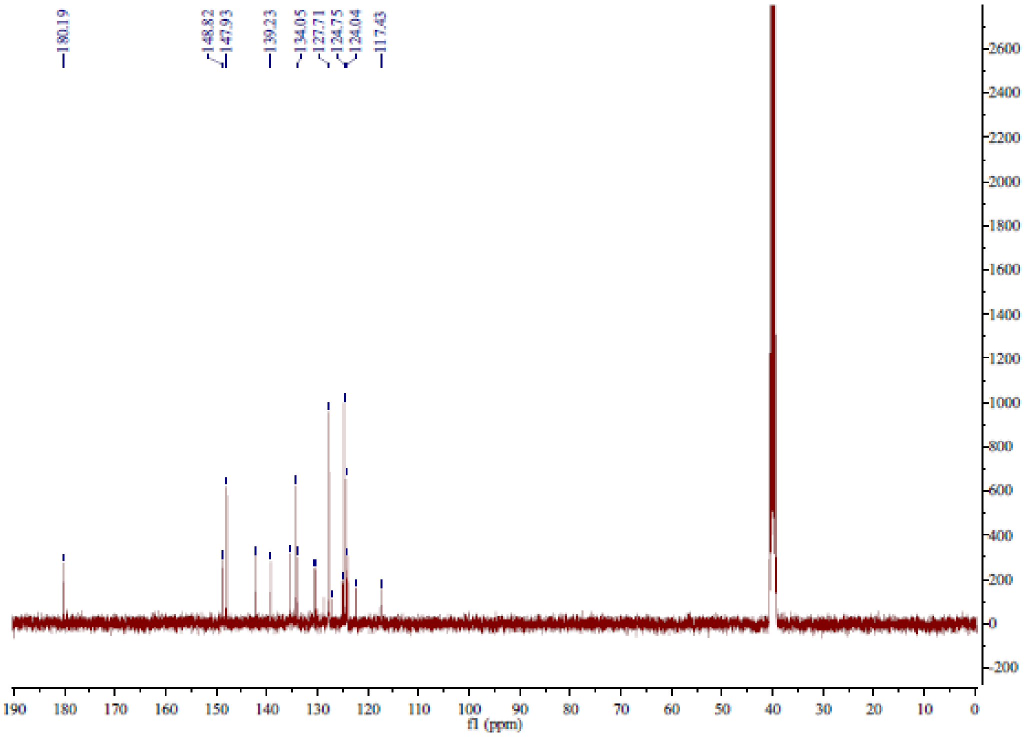

***N*-1,3-benzodioxol-5-yl-*N'*-(4-pyridin-3-ylphenyl)thiourea (DATU10)**

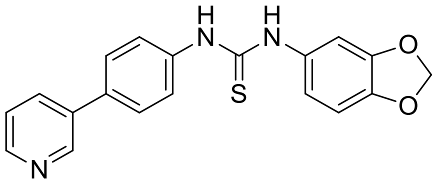

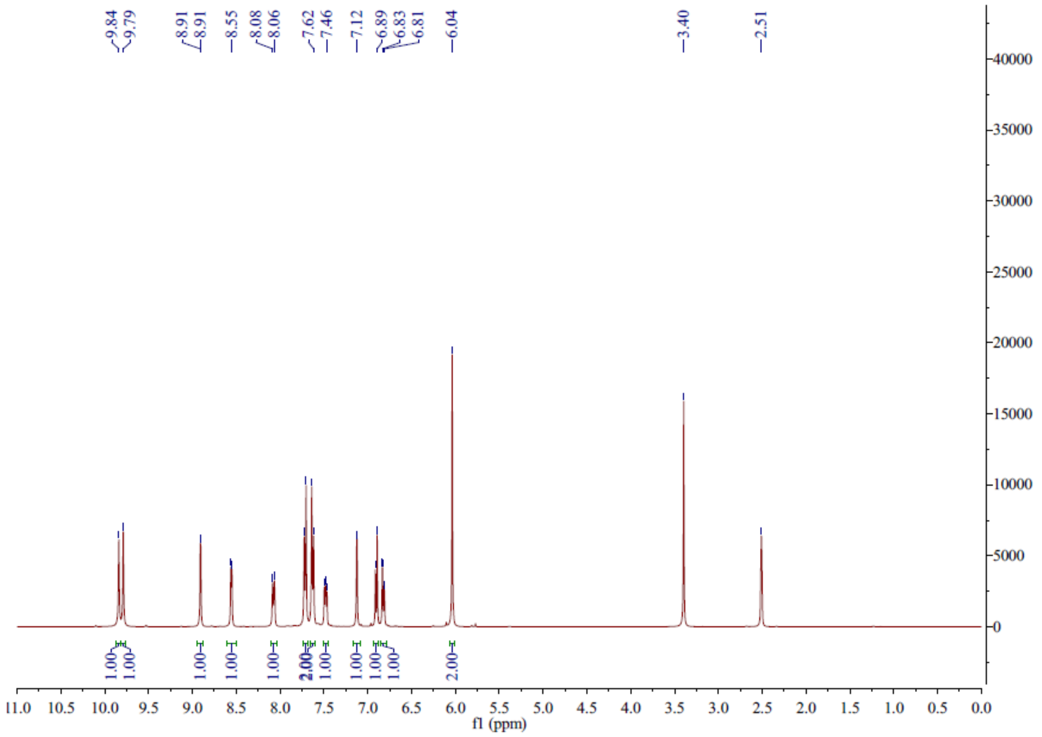

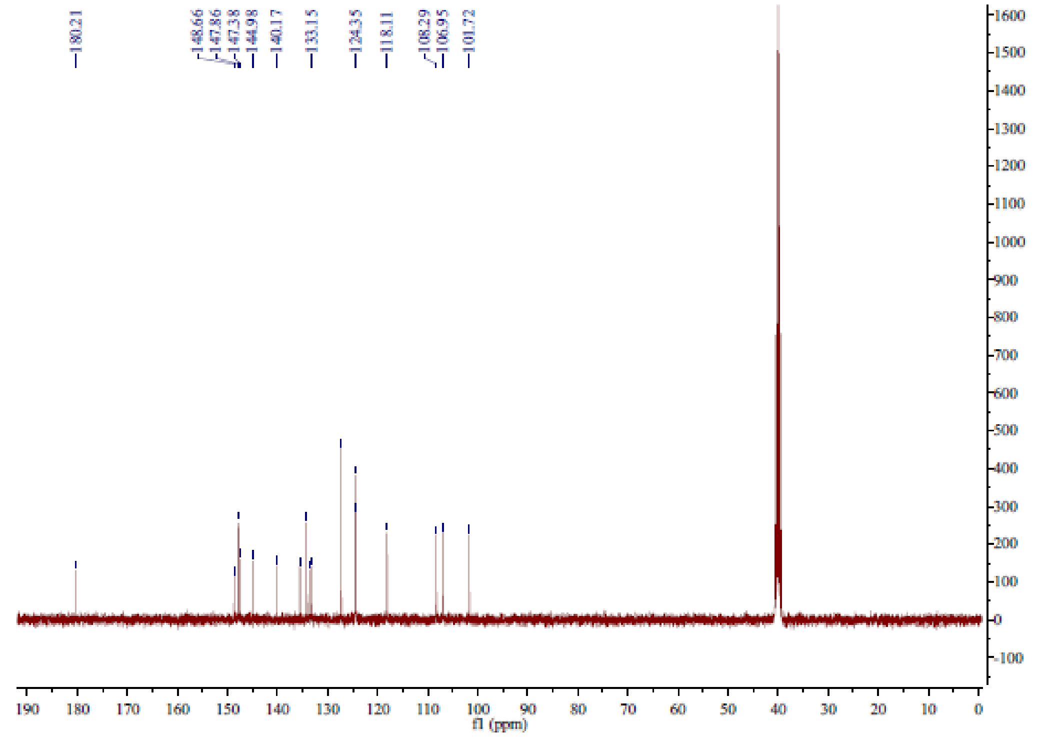

1-(4-(4-oxo-3,4-dihydroquinazolin-7-yl)phenyl)-3-(3-(trifluoromethyl)phenyl)urea (QDAU-1)

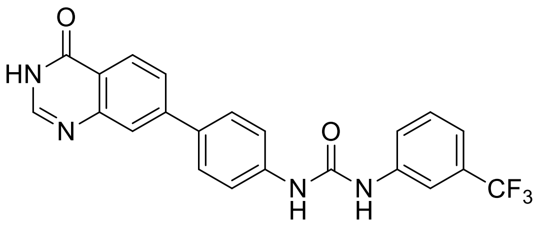

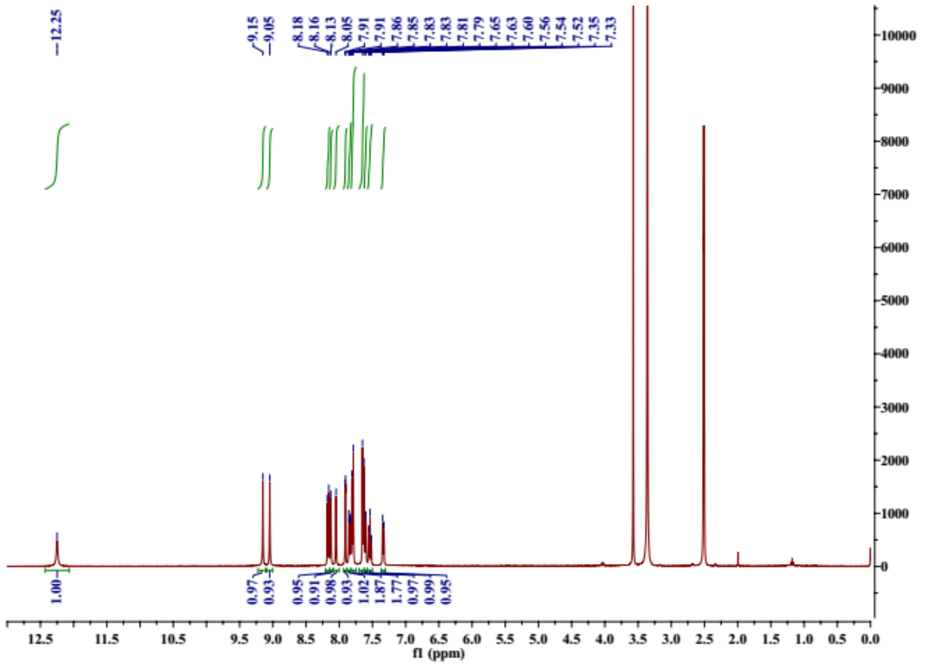

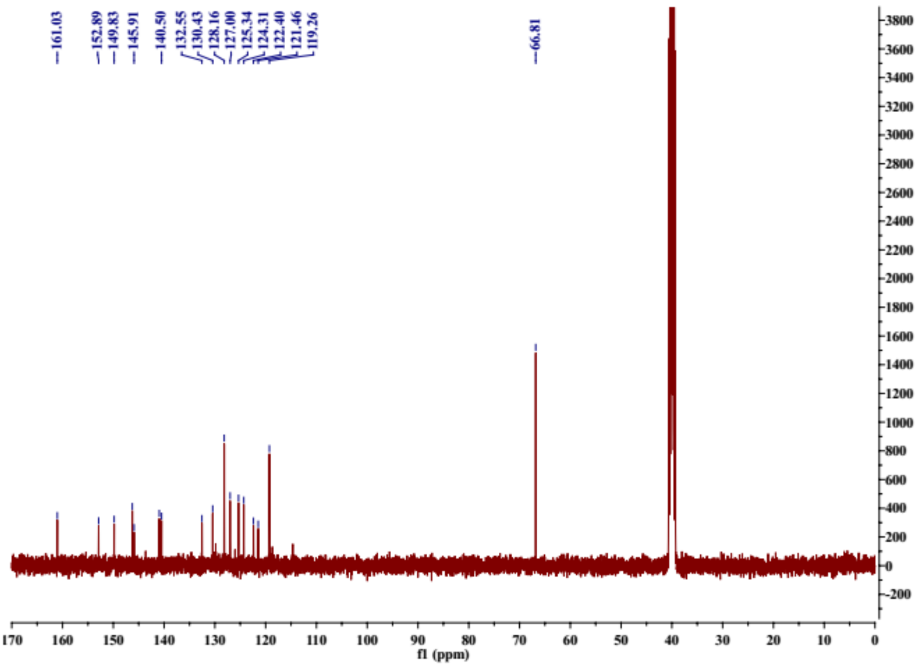

1-(3-chlorophenyl)-3-(4-(4-oxo-3,4-dihydroquinazolin-7-yl)phenyl)urea(QDAU-2)

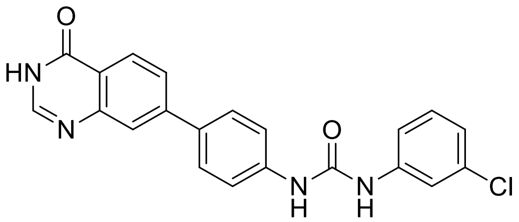

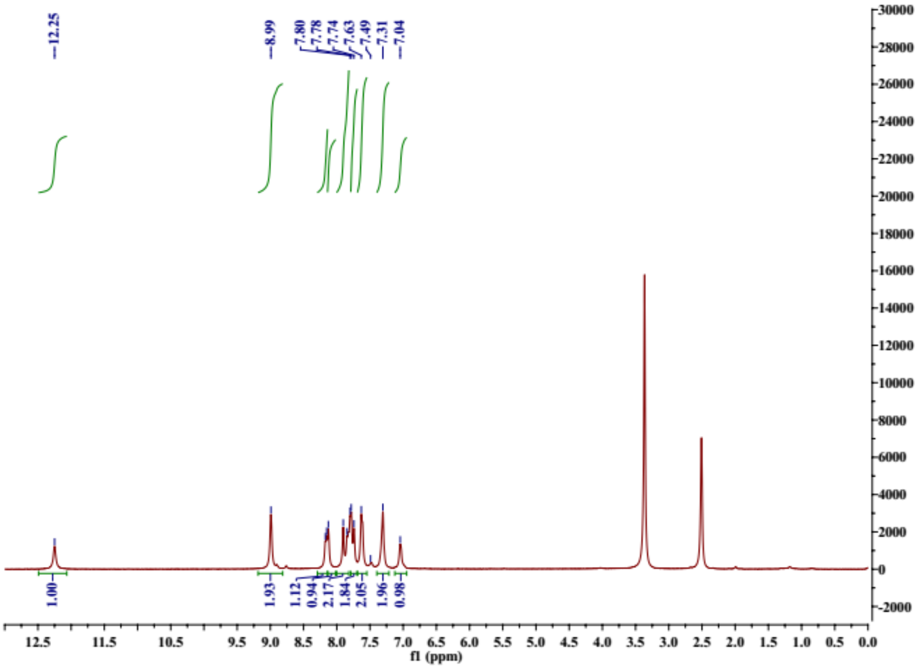

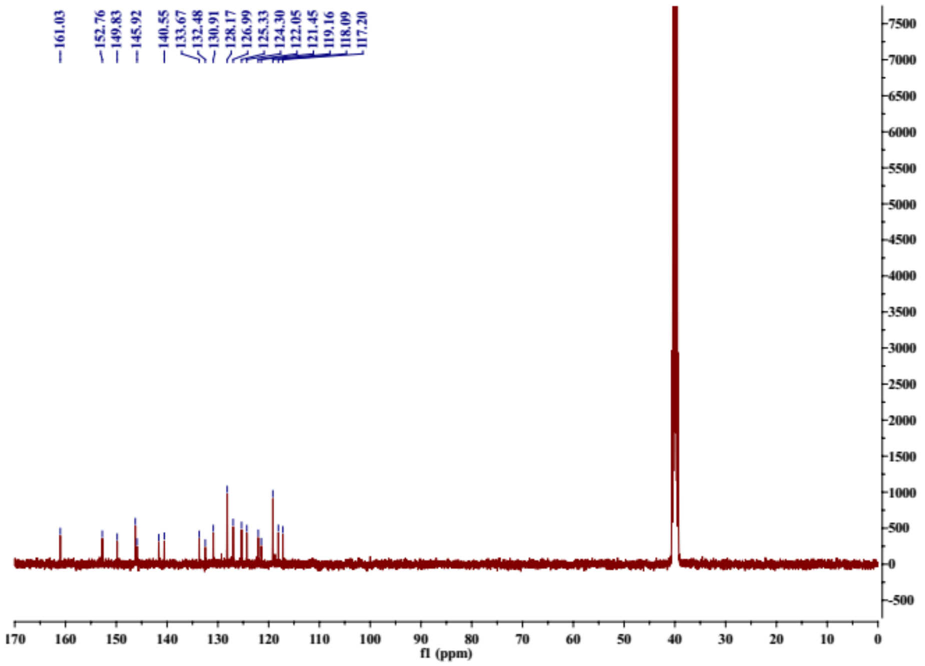

1-(3-fluorophenyl)-3-(4-(4-oxo-3,4-dihydroquinazolin-7-yl)phenyl)ure(QDAU-3)

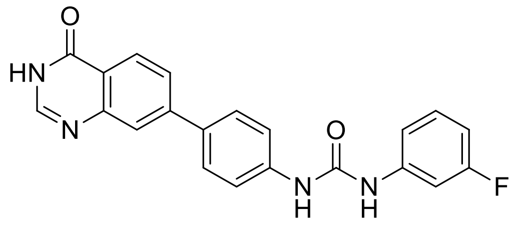

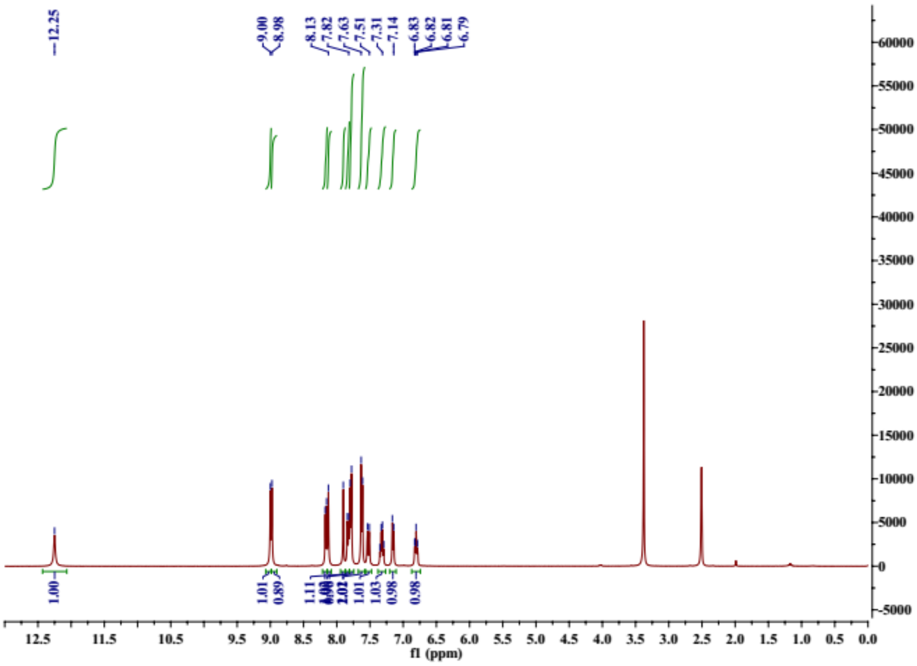

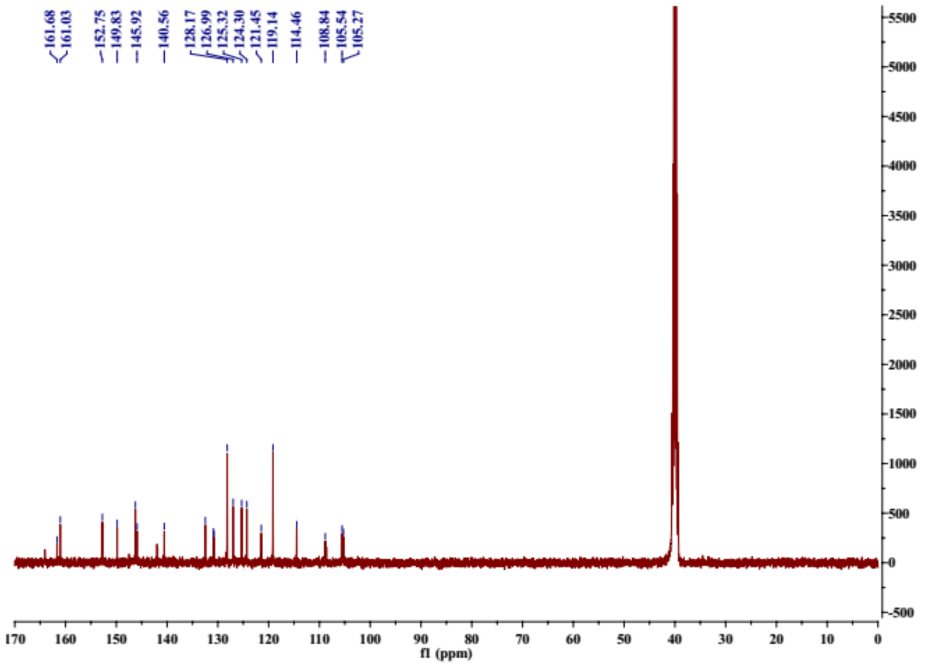

1-(2-fluorophenyl)-3-(4-(4-oxo-3,4-dihydroquinazolin-7-yl)phenyl)urea(QDAU-4)

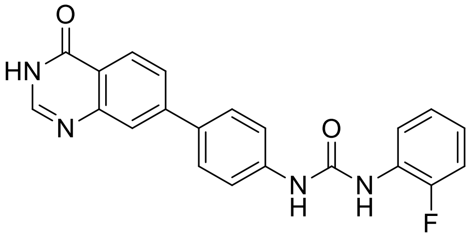

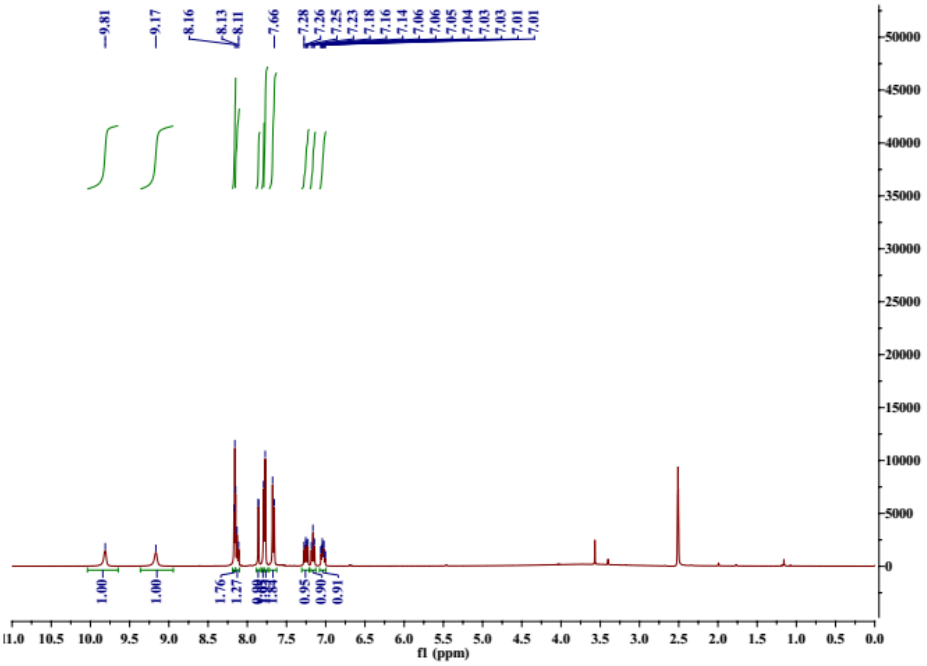

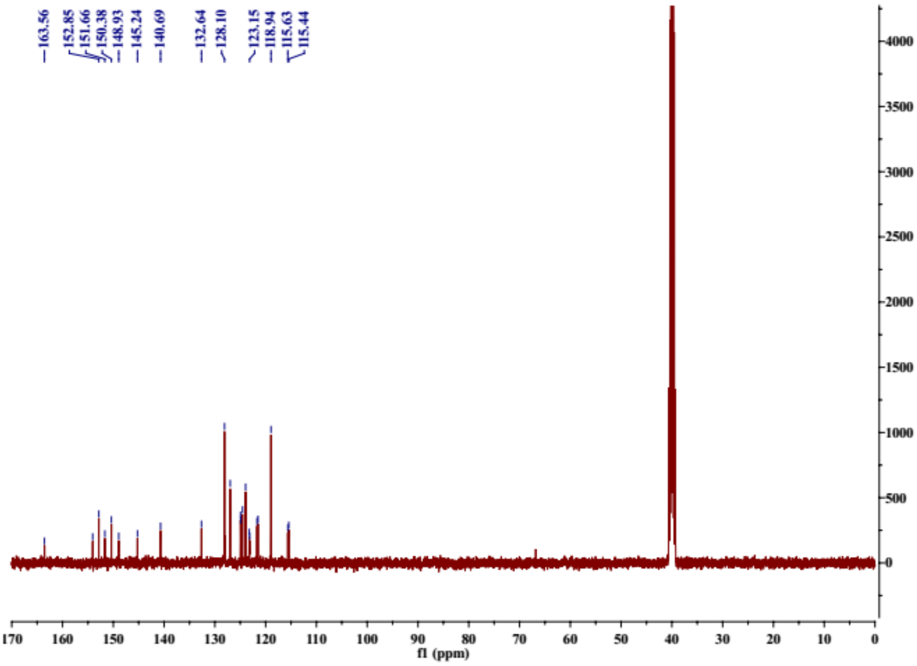

1-(3,4-difluorophenyl)-3-(4-(4-oxo-3,4-dihydroquinazolin-7-yl)phenyl)urea(QDAU-5)

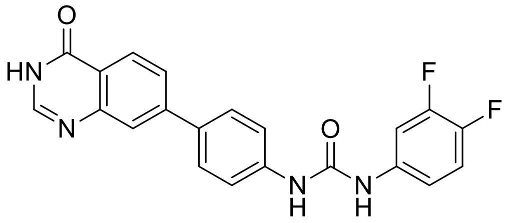

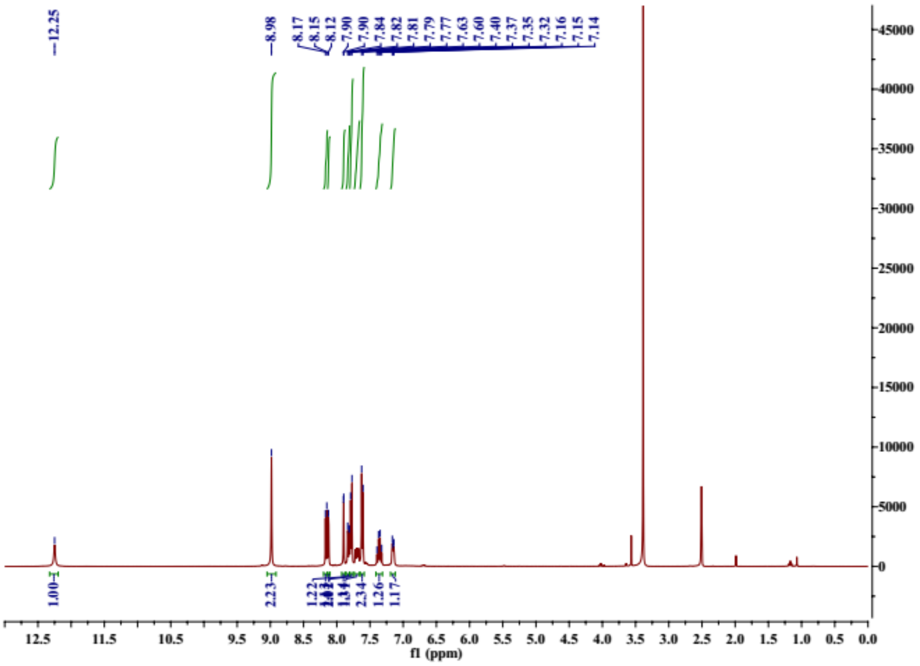

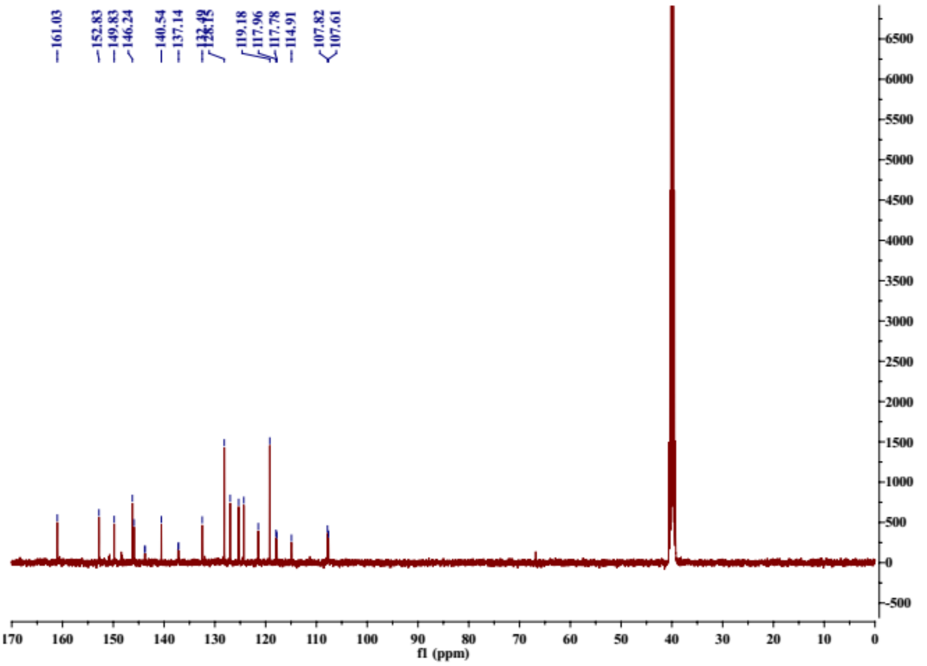

1-(4-fluorophenyl)-3-(4-(4-oxo-3,4-dihydroquinazolin-7-yl)phenyl)urea(QDAU-6)

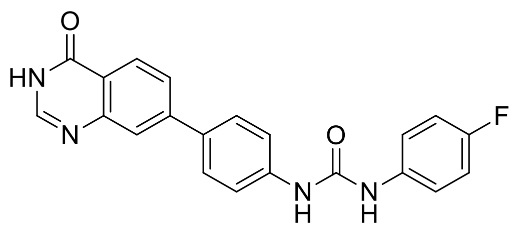
O
HN
N
O
N
H
N
H
F

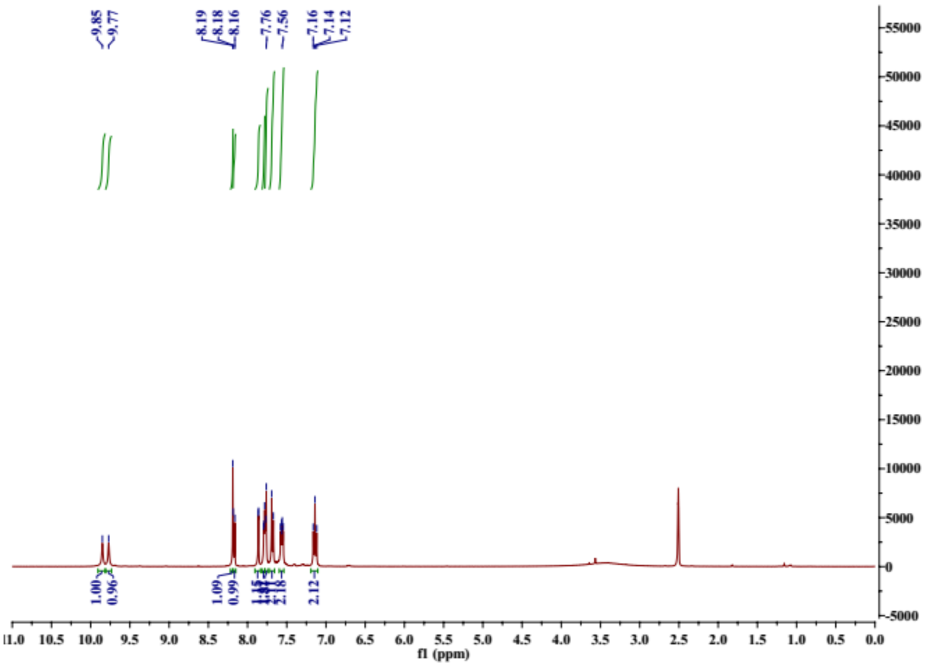
9.85
9.77
8.19
8.18
8.16
7.76
7.56
7.16
7.14
7.12
1.00
0.96
1.09
0.99
2.18
2.12
55000
50000
45000
40000
35000
30000
25000
20000
15000
10000
5000
0
-5000
11.0 10.5 10.0 9.5 9.0 8.5 8.0 7.5 7.0 6.5 6.0 5.5 5.0 4.5 4.0 3.5 3.0 2.5 2.0 1.5 1.0 0.5 0.0
f1 (ppm)

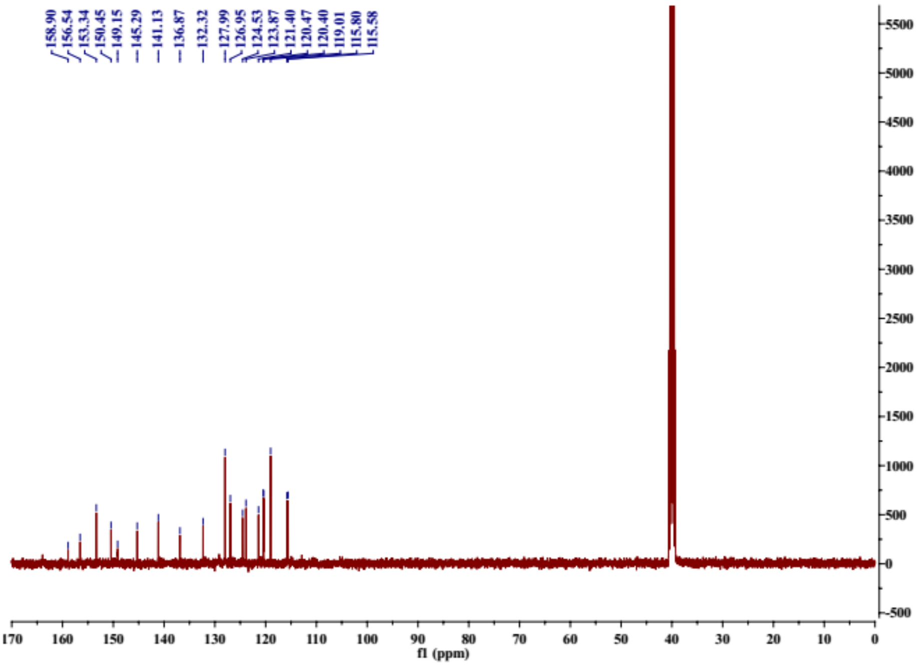
158.90
156.54
153.34
150.45
149.15
145.29
141.13
136.87
132.32
127.99
126.95
124.53
123.87
121.40
120.47
120.40
119.01
115.80
115.58
5500
5000
4500
4000
3500
3000
2500
2000
1500
1000
500
0
-500
170 160 150 140 130 120 110 100 90 80 70 60 50 40 30 20 10 0
f1 (ppm)

1-(4-(4-oxo-3,4-dihydroquinazolin-7-yl)phenyl)-3-(4-(trifluoromethoxy)phenyl)urea (QDAU-7)

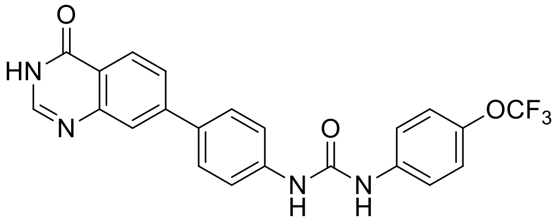

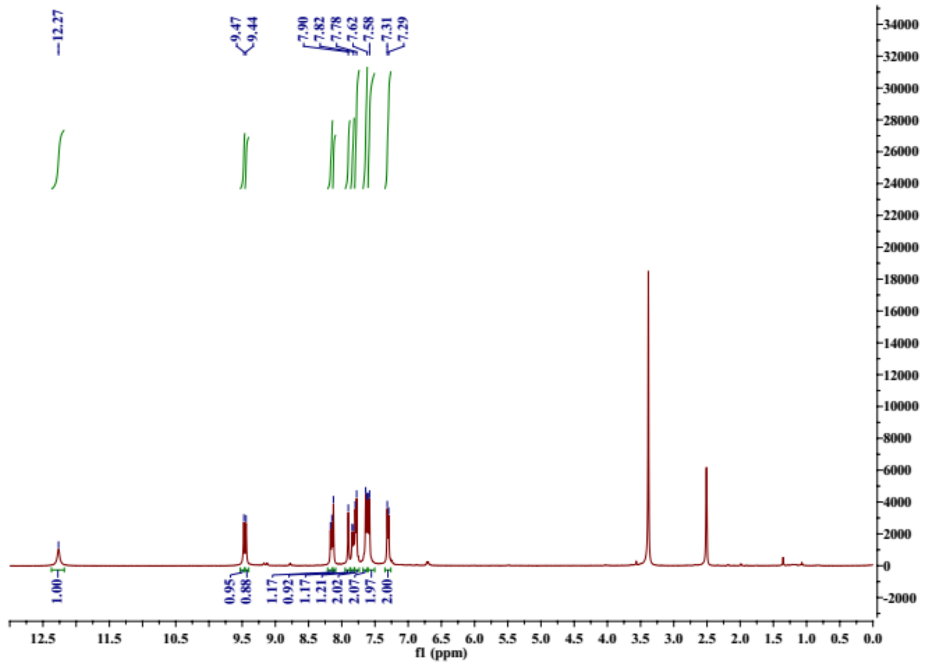

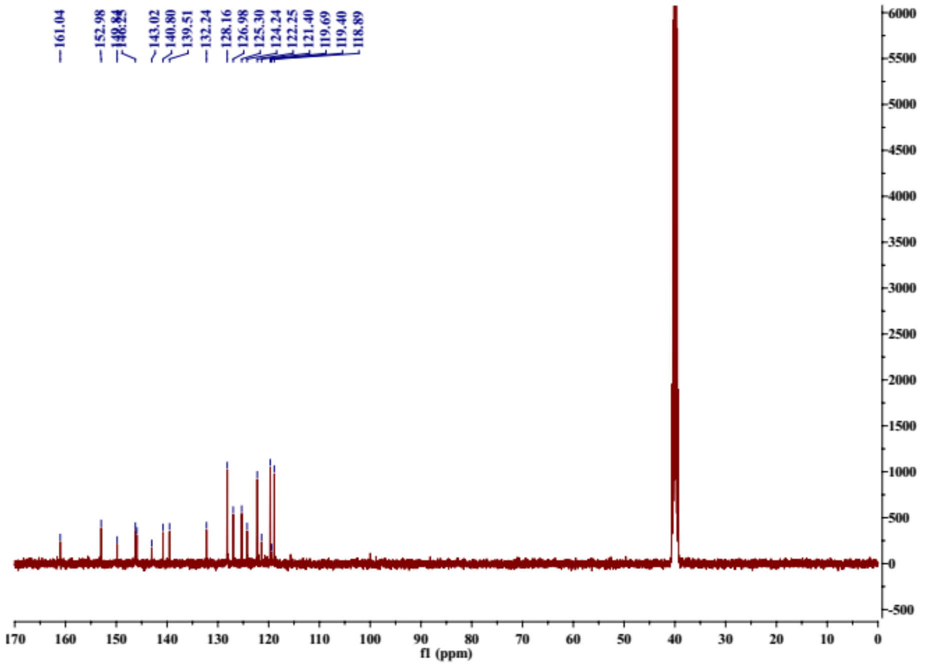

1-(3-isopropylphenyl)-3-(4-(4-oxo-3,4-dihydroquinazolin-7-yl)phenyl)urea(QDAU-8)

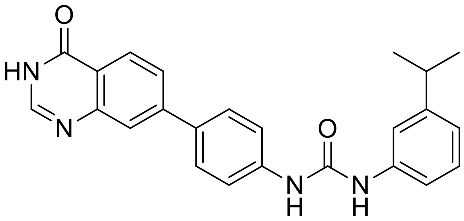

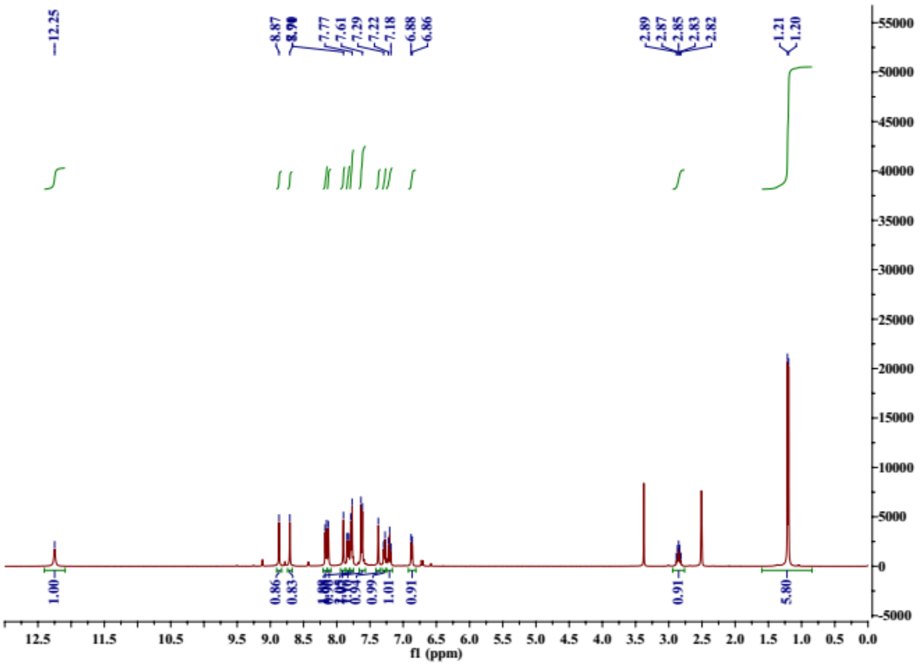

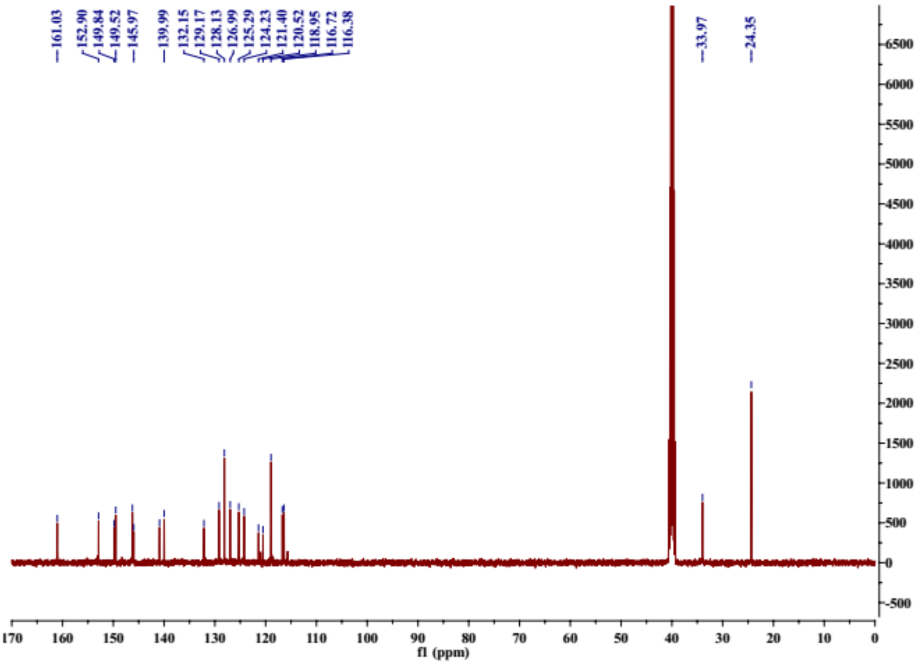

1-(4-chloro-3-(trifluoromethyl)phenyl)-3-(4-(4-oxo-3,4-dihydroquinazolin-7-yl)phenyl)urea (QDAU-9)

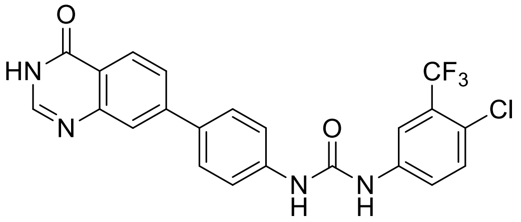

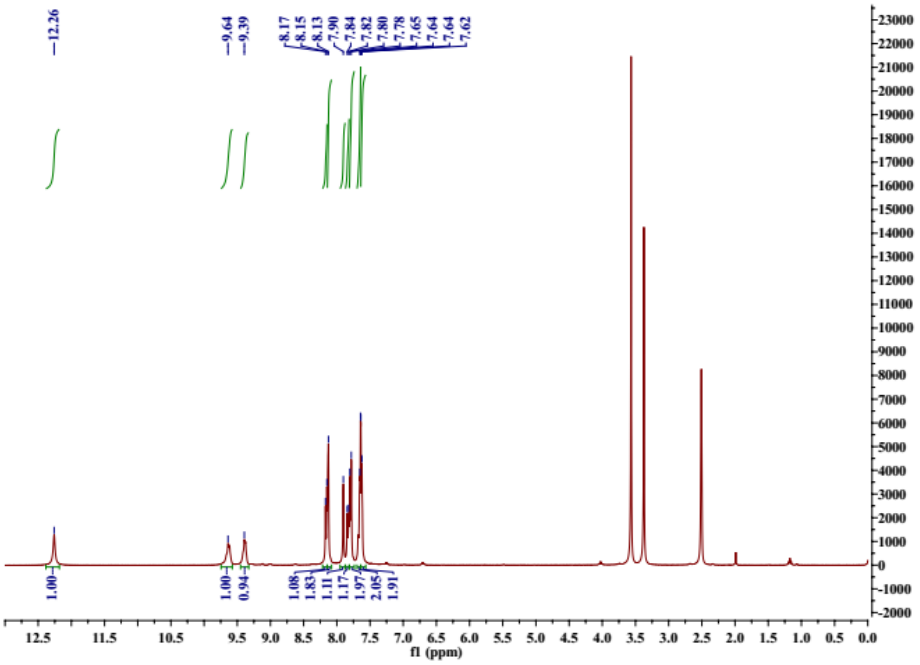

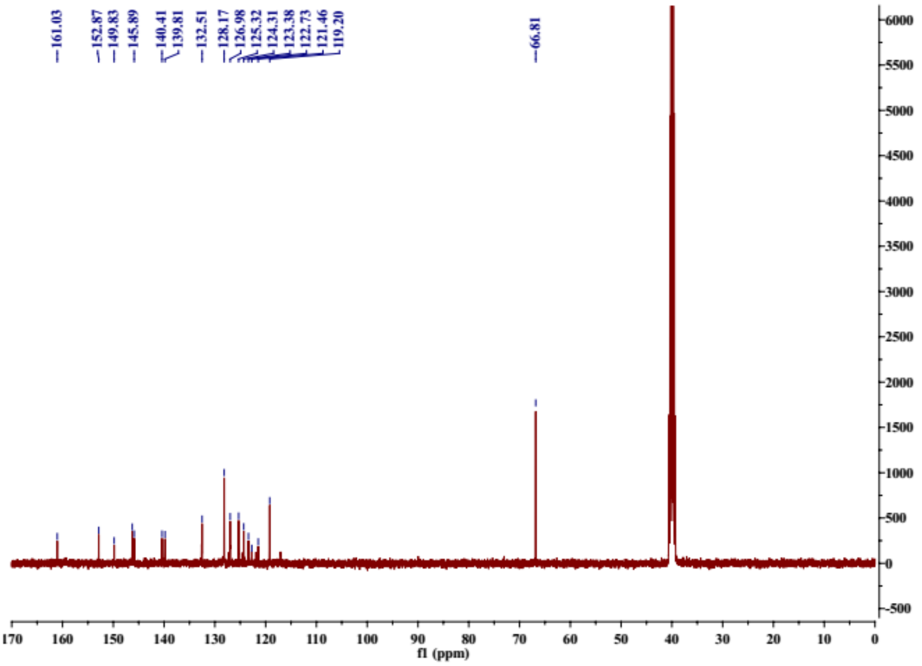

1-(4-(4-oxo-3,4-dihydroquinazolin-7-yl)phenyl)-3-(4-(trifluoromethyl)phenyl)urea (QDAU-10)

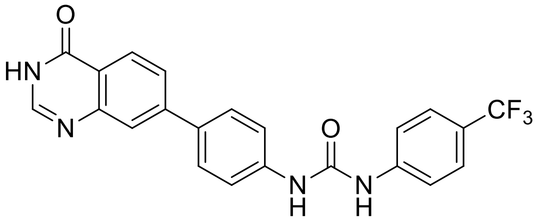

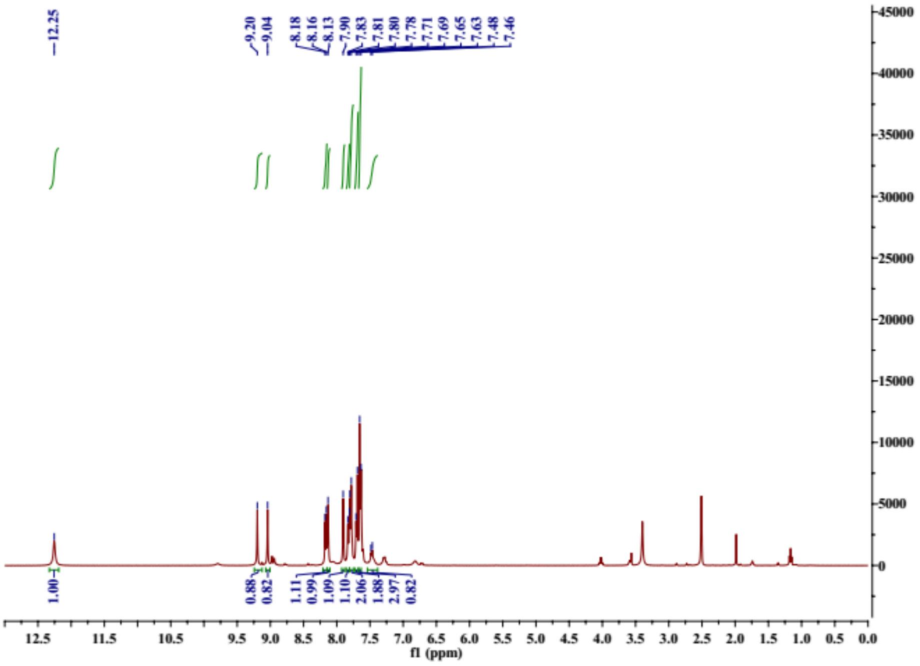

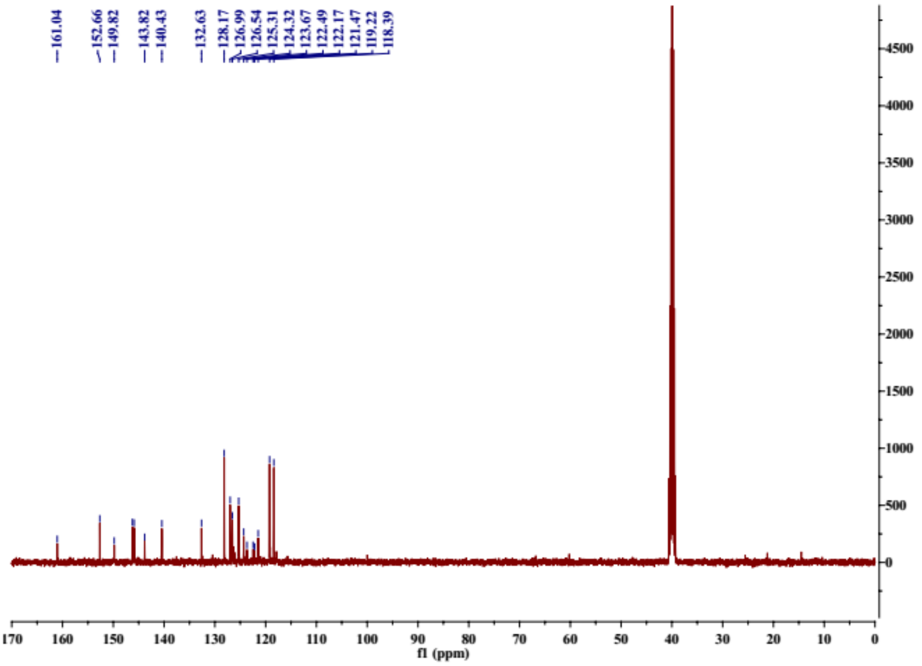

DATU1

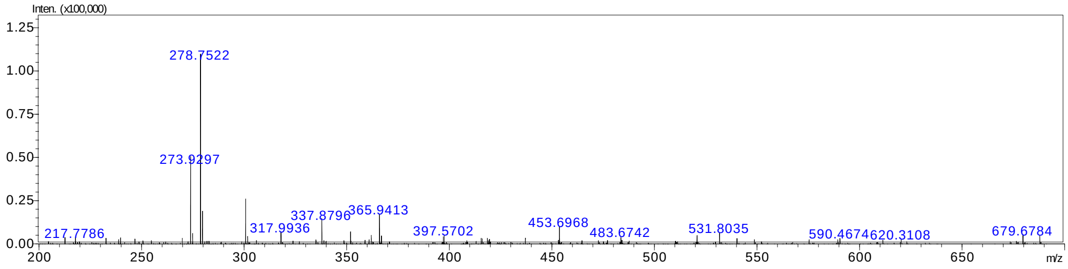

DATU 2

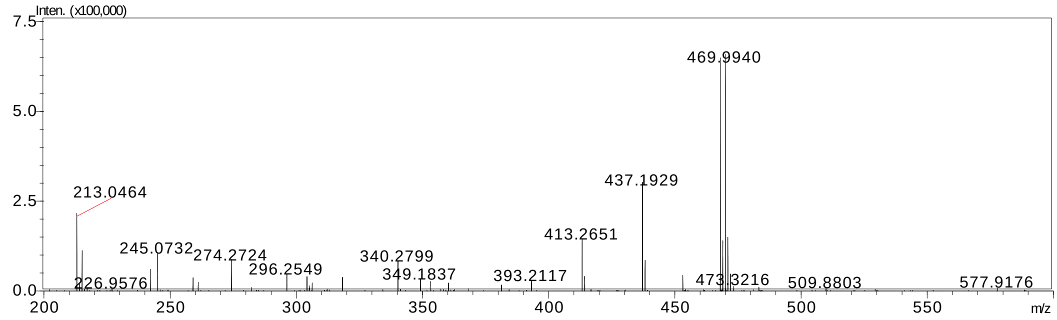

DATU 3

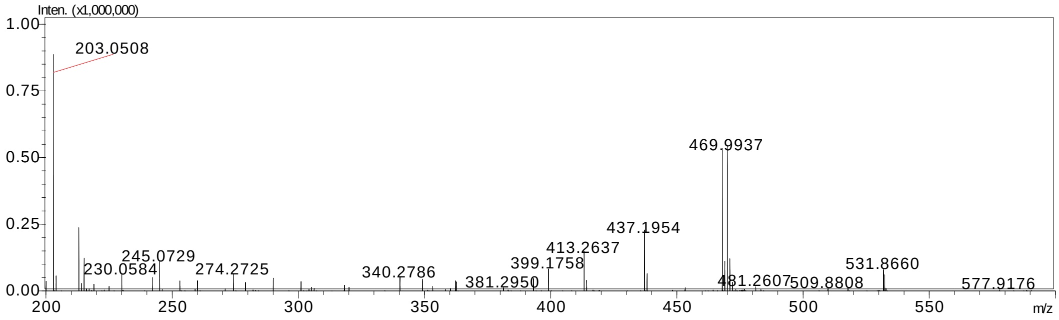

DATU 4

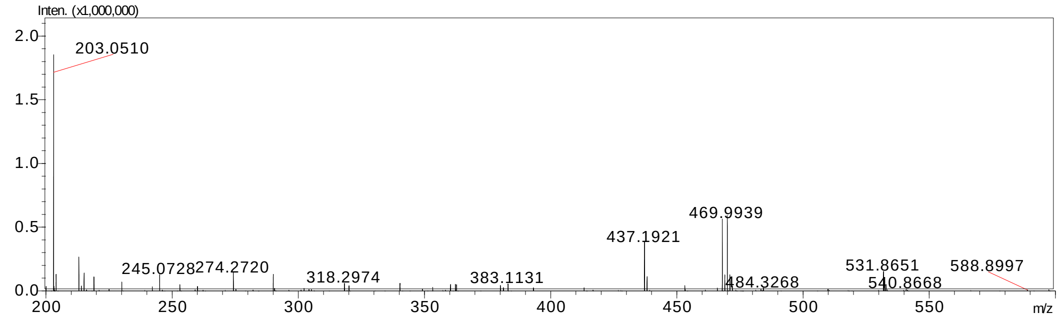

DATU 5

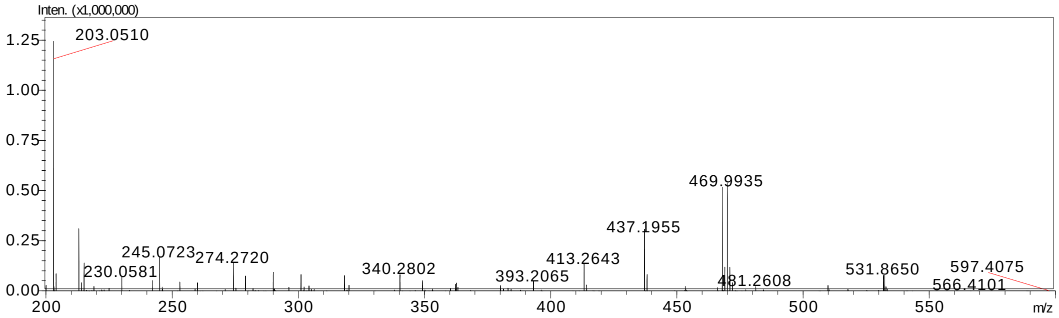

DATU 6

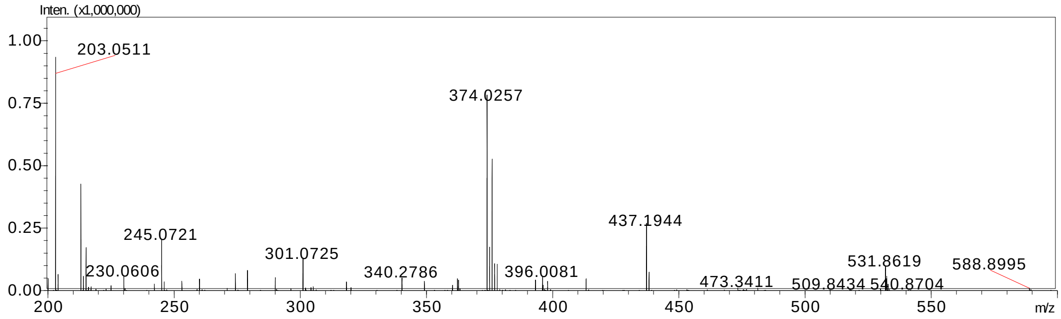

DATU 7

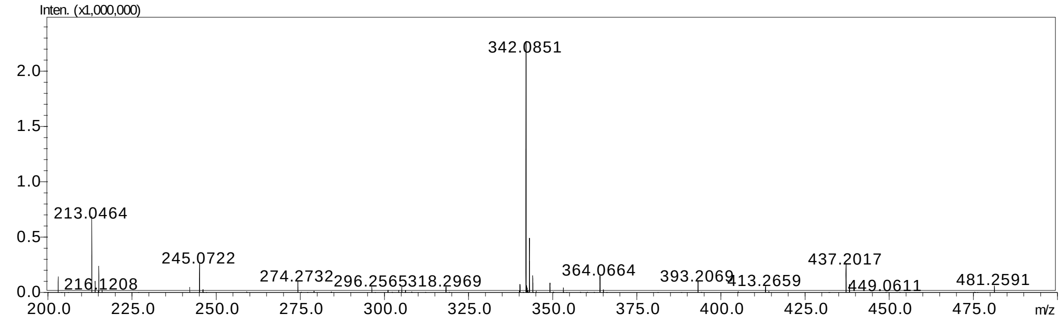

DATU 8

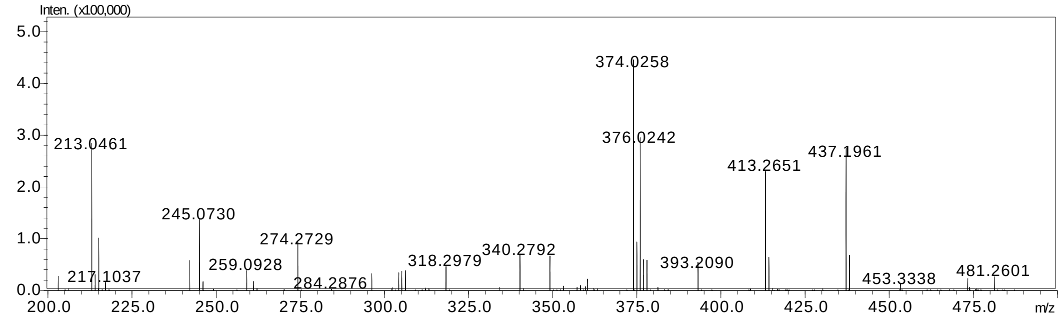

DATU 9

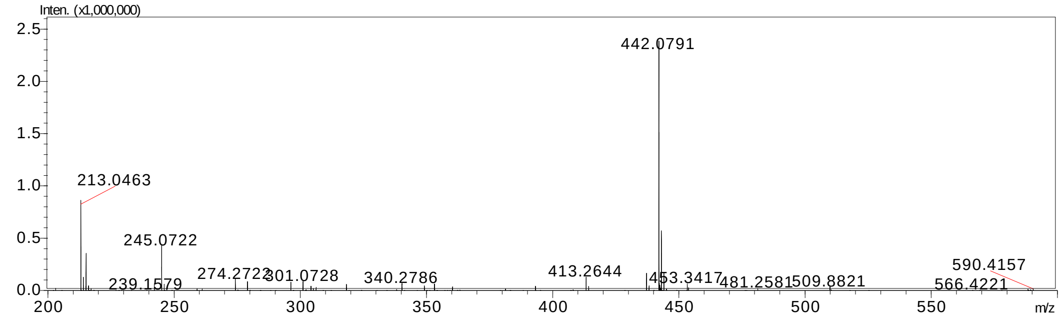

DATU 10

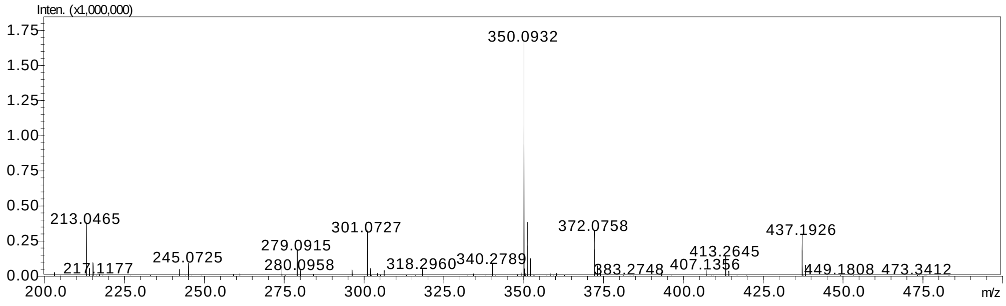

QDAU-1

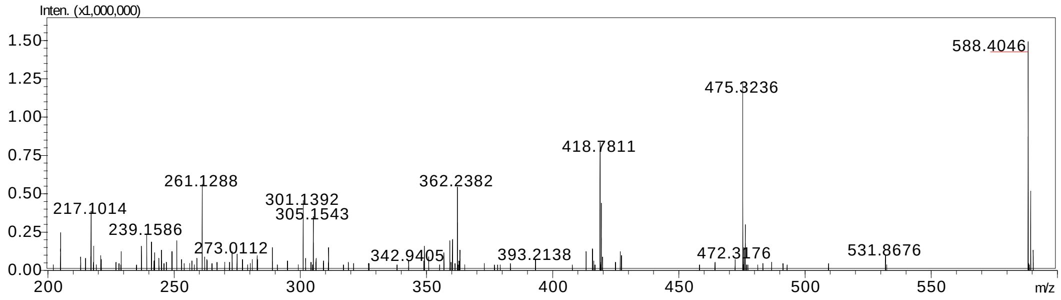

QDAU-2

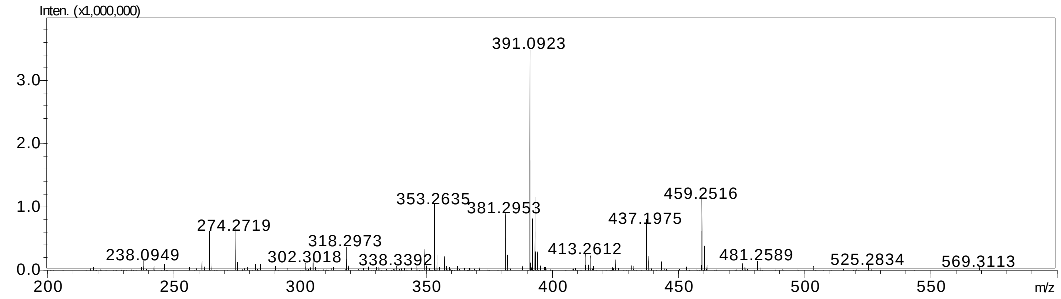

QDAU-3

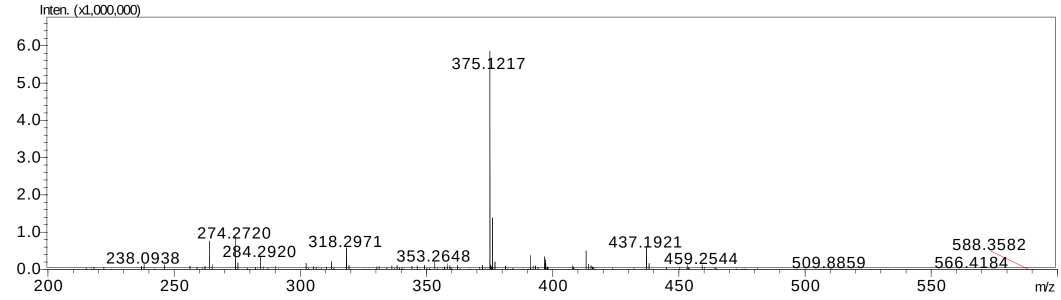

QDAU-4

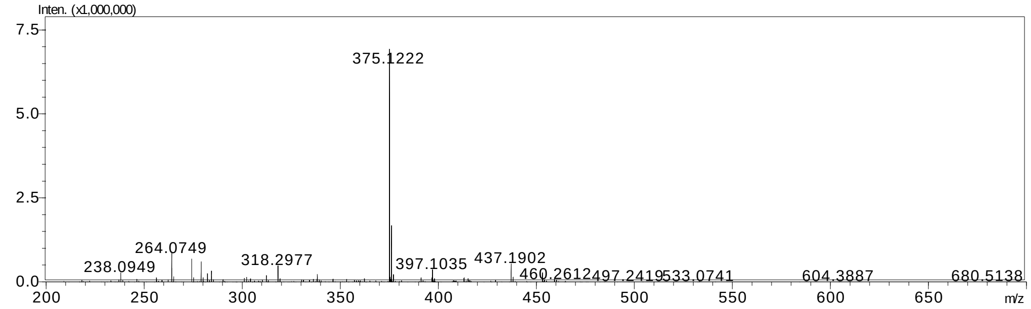

QDAU-5

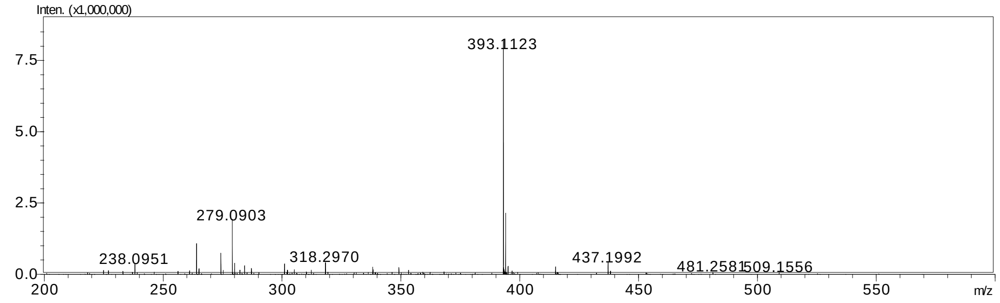

QDAU-6

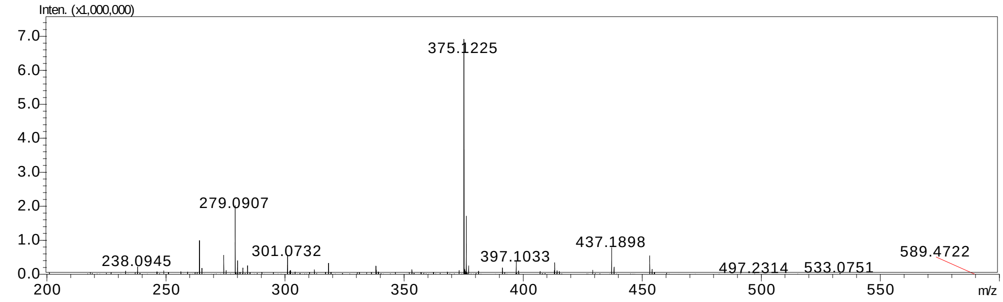

QDAU-7

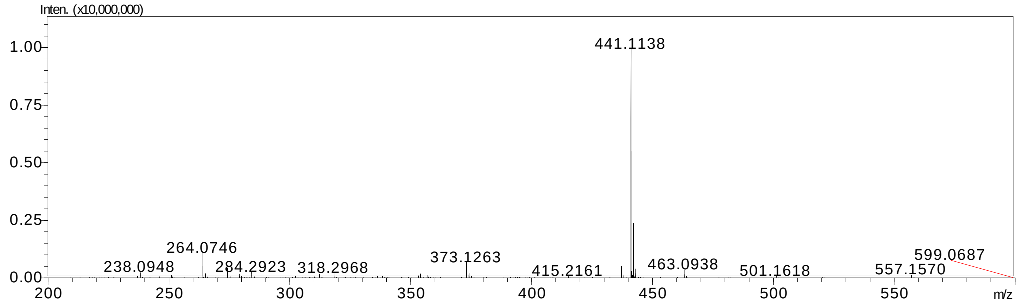

QDAU-8

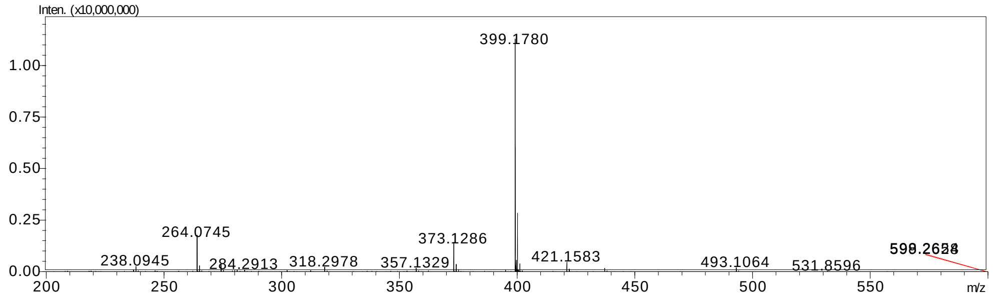

QDAU-9

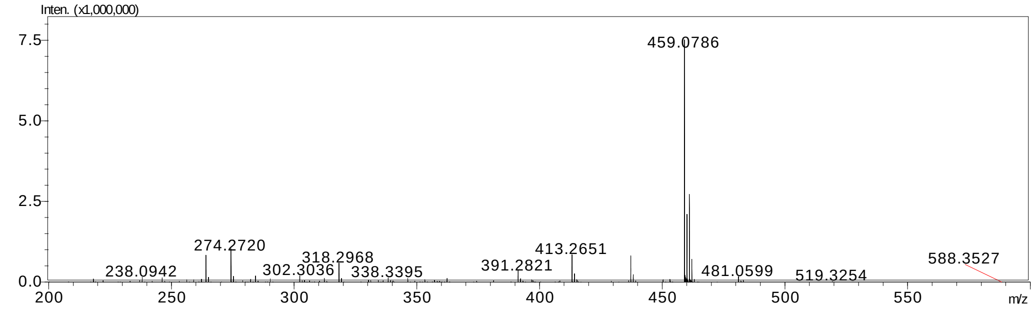

QDAU-10

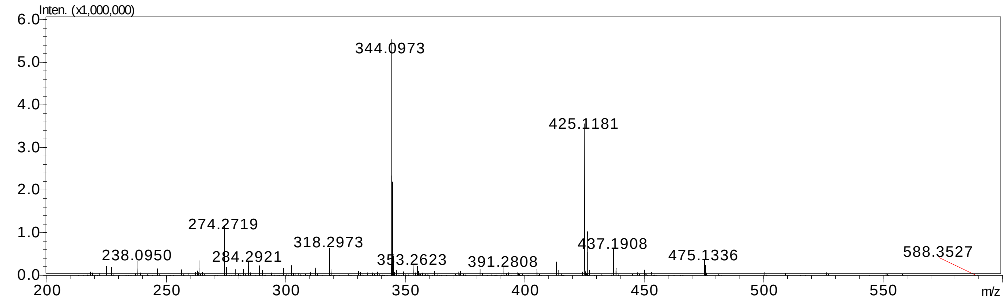

DATU1

Reverse Phase (Method：0-8min 60:40 CH_3CN : H_2O；8-9min 90:10 CH_3CN : H_2O；9-10min 60:40 CH_3CN : H_2O；Flow rate 1.0 mL / min)

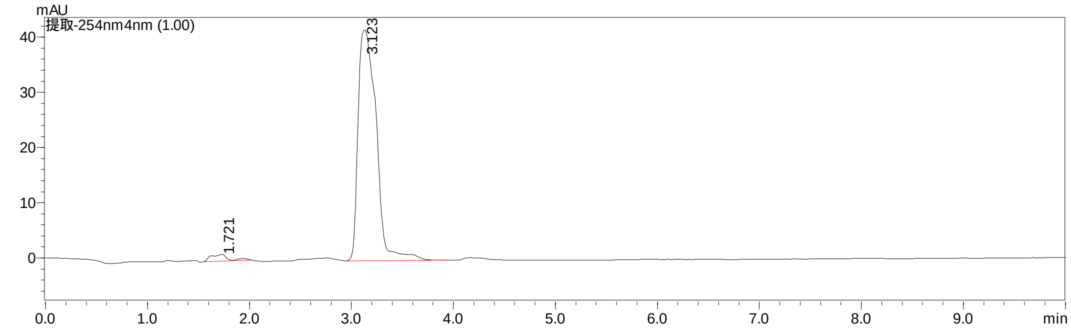

No.	Retention Time	Peak height	Area	Percent
1	1.721	1253	15052	2.8318
2	3.123	41752	516482	97.1682
Total				100

DATU2

Reverse Phase (Method：0-8min 60:40 CH_3CN : H_2O；8-9min 90:10 CH_3CN : H_2O；9-10min 60:40 CH_3CN : H_2O；Flow rate 1.0 mL / min)

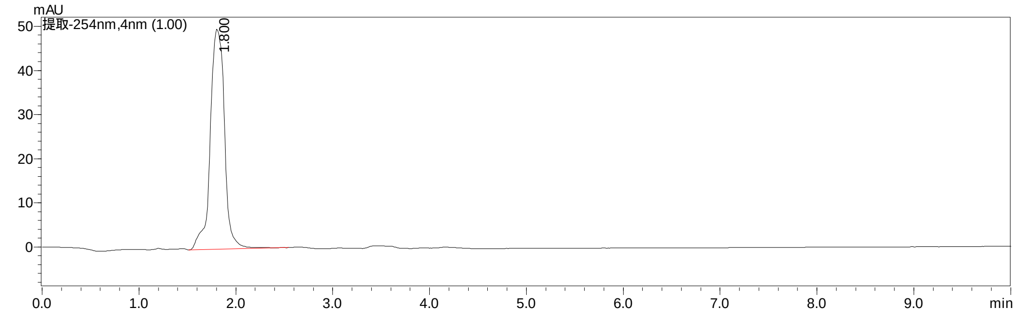

No.	Retention Time	Peak height	Area	Percent
1	1.800	49849	511168	100.0000
Total				100

DATU3

Reverse Phase (Method：0-8min 60:40 CH_3CN : H_2O；8-9min 90:10 CH_3CN : H_2O；9-10min 60:40 CH_3CN : H_2O；Flow rate 1.0 mL / min)

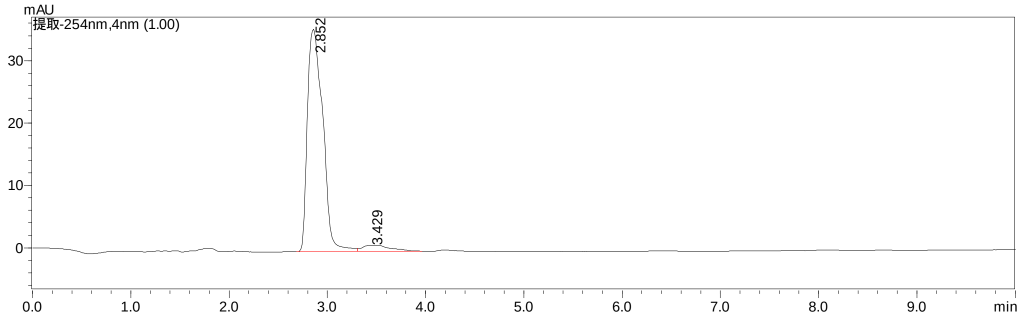

No.	Retention Time	Peak height	Area	Percent
1	2.852	35664	389761	95.3111
**2**	3.429	953	19175	4.6889
Total				100

DATU4

Reverse Phase (Method：0-8min 60:40 CH_3CN : H_2O；8-9min 90:10 CH_3CN : H_2O；9-10min 60:40 CH_3CN : H_2O；Flow rate 1.0 mL / min)

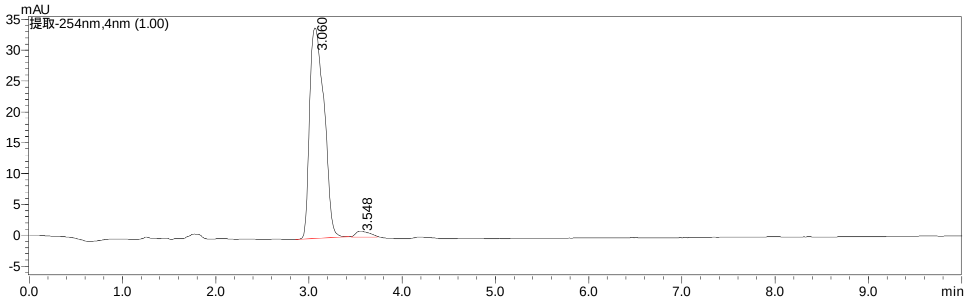

No.	Retention Time	Peak height	Area	Percent
1	3.060	34114	369883	97.2824
**2**	3.548	973	10333	2.7176
Total				100

DATU5

Reverse Phase (Method：0-8min 60:40 CH_3CN : H_2O；8-9min 90:10 CH_3CN : H_2O；9-10min 60:40 CH_3CN : H_2O；Flow rate 1.0 mL / min)

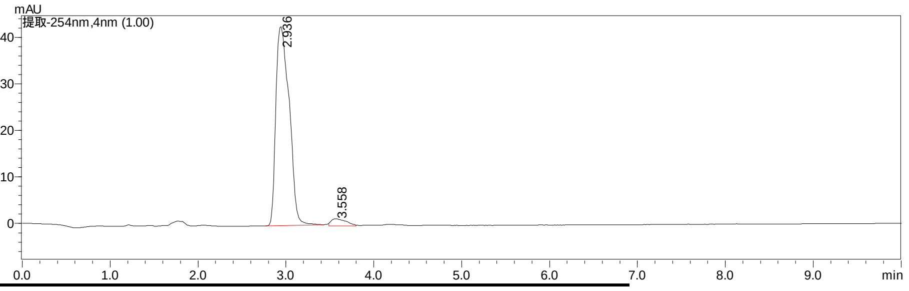

No.	Retention Time	Peak height	Area	Percent
1	2.936	42762	451676	96.1895
**2**	3.558	1506	17893	3.8105
Total				100

DATU6

Reverse Phase (Method：0-8min 60:40 CH_3CN : H_2O；8-9min 90:10 CH_3CN : H_2O；9-10min 60:40 CH_3CN : H_2O；Flow rate 1.0 mL / min)

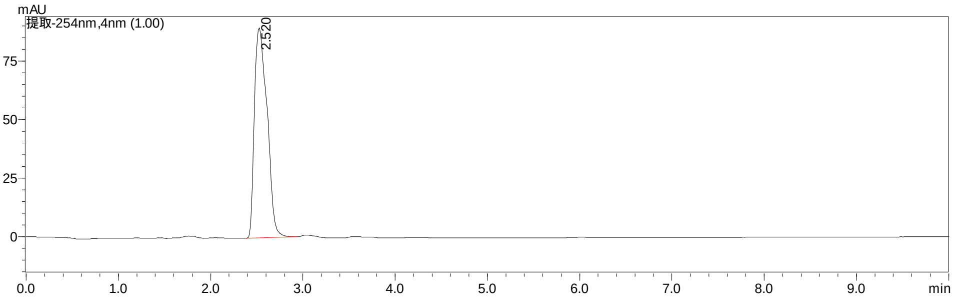

No.	Retention Time	Peak height	Area	Percent
1	2.520	89645	855723	100.0000
Total				100

DATU7

Reverse Phase (Method：0-8min 60:40 CH_3CN : H_2O；8-9min 90:10 CH_3CN : H_2O；9-10min 60:40 CH_3CN : H_2O；Flow rate 1.0 mL / min)

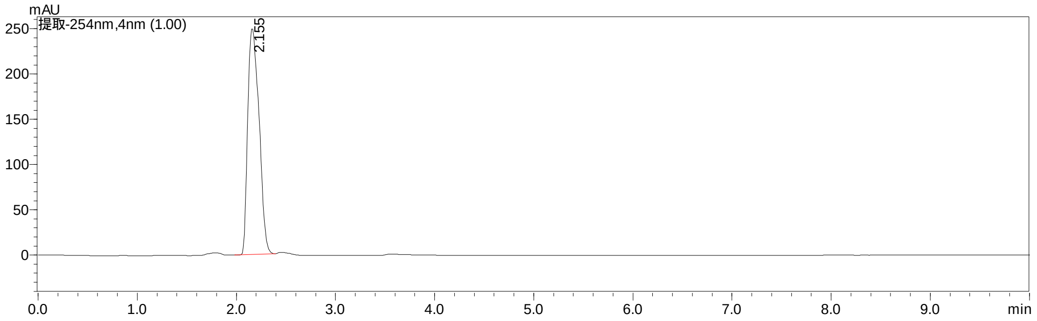

No.	Retention Time	Peak height	Area	Percent
1	2.155	248936	1985944	100.0000
Total				100

DATU8

Reverse Phase (Method：0-8min 60:40 CH_3CN : H_2O；8-9min 90:10 CH_3CN : H_2O；9-10min 60:40 CH_3CN : H_2O；Flow rate 1.0 mL / min)

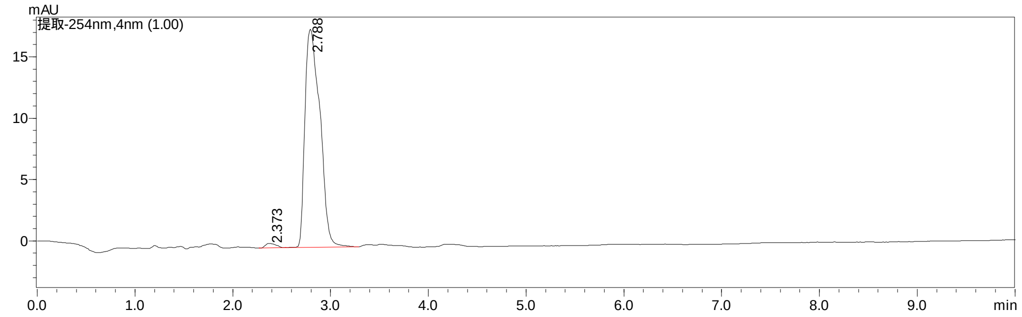

No.	Retention Time	Peak height	Area	Percent
1	2.373	378	2946	1.5238
2	2.788	17790	190373	98.4762
Total				100

DATU9

Reverse Phase (Method：0-8min 60:40 CH_3CN : H_2O；8-9min 90:10 CH_3CN : H_2O；9-10min 60:40 CH_3CN : H_2O；Flow rate 1.0 mL / min)

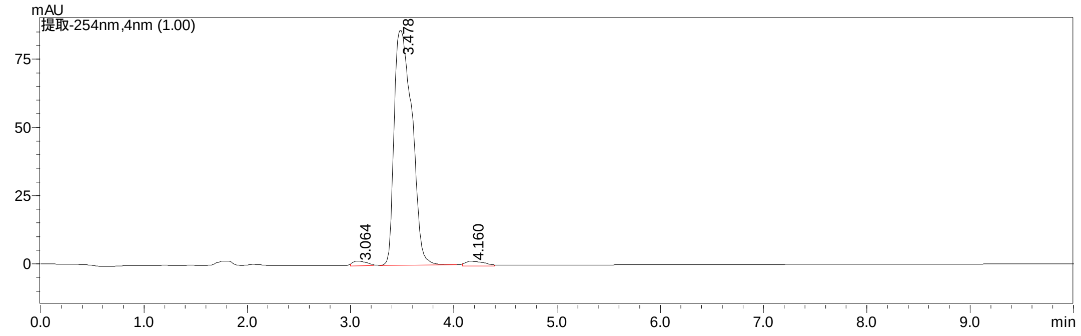

No.	Retention Time	Peak height	Area	Percent
1	3.064	1735	16274	1.5116
2	3.478	86097	1036775	96.3020
3	4.160	1787	23538	2.1864
Total				100

DATU10

Reverse Phase (Method：0-8min 60:40 CH_3CN : H_2O；8-9min 90:10 CH_3CN : H_2O；9-10min 60:40 CH_3CN : H_2O；Flow rate 1.0 mL / min)

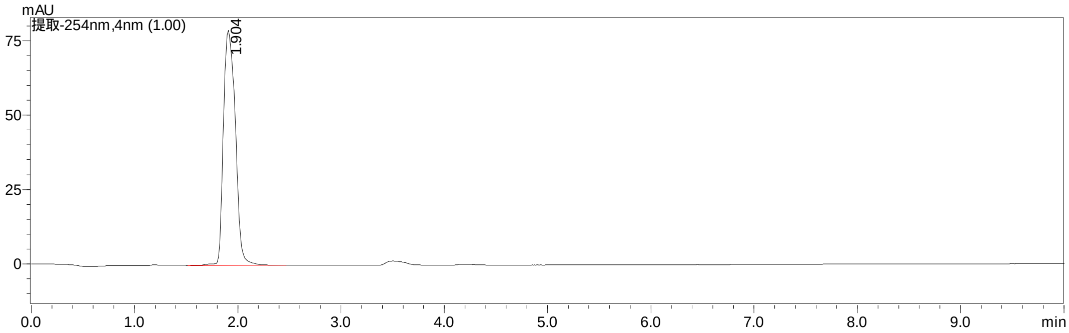

No.	Retention Time	Peak height	Area	Percent
1	1.904	79057	641551	100.0000
Total				100

QDAU-1

Reverse Phase (Method：0-8min 60:40 CH_3CN : H_2O；8-9min 90:10 CH_3CN : H_2O；9-10min 60:40 CH_3CN : H_2O；Flow rate 1.0 mL / min)

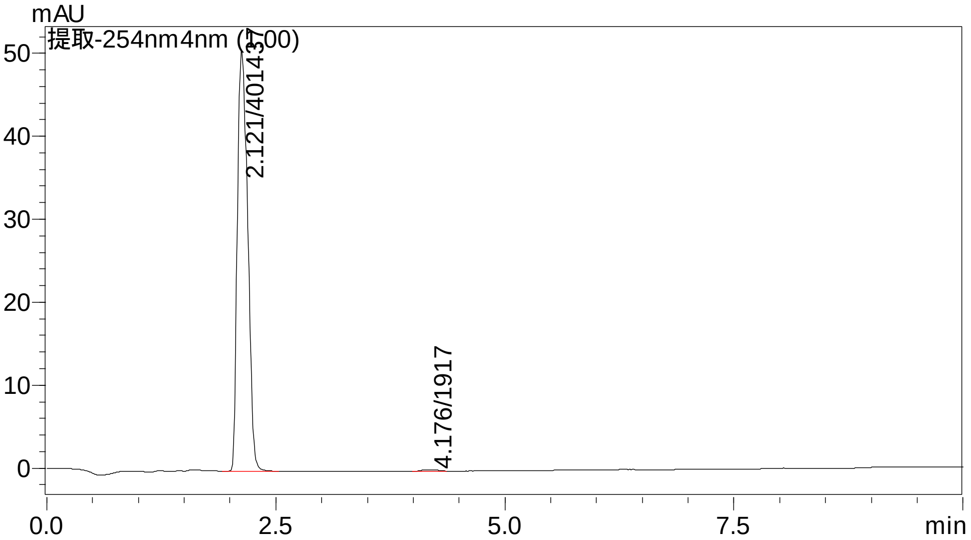

No.	Retention Time	Peak height	Area	Percent
1	2.121	50821	401437	99.5247
2	4.176	160	1917	0.4753
Total				100

QDAU-2

Reverse Phase (Method 90:10 CH_3CN : H_2O, Flow rate 1.0 mL / min)

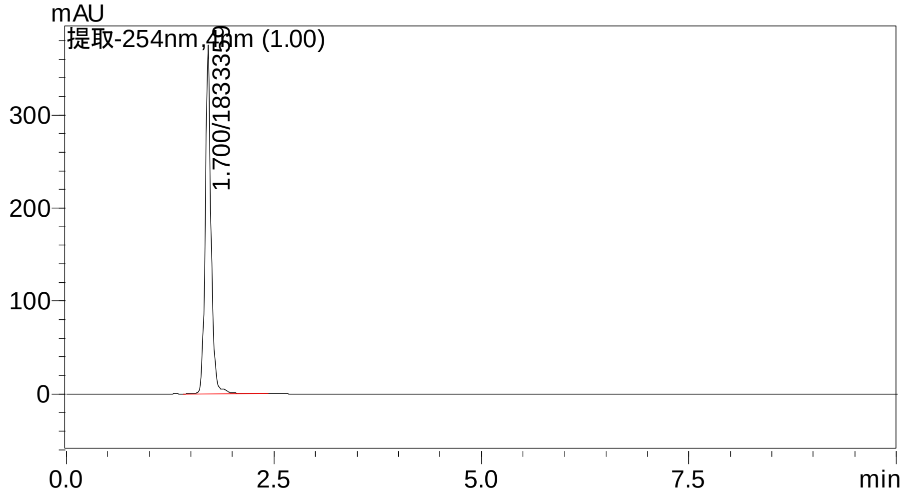

No.	Retention Time	Peak height	Area	Percent
1	1.700	375175	1833359	100.0000
Total				100

QDAU-3

Reverse Phase (Method：0-8min 60:40 CH_3CN : H_2O；8-9min 90:10 CH_3CN : H_2O；9-10min 60:40 CH_3CN : H_2O；Flow rate 1.0 mL / min)

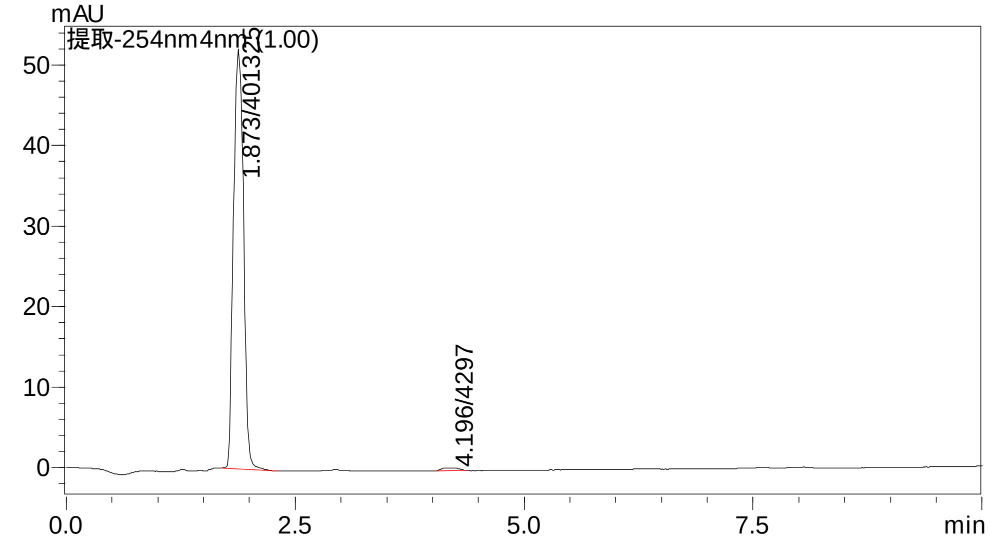

No.	Retention Time	Peak height	Area	Percent
1	1.873	52154	401325	98.9407
2	4.196	348	4297	1.0593
Total				100

QDAU-4

Reverse Phase (Method：0-8min 60:40 CH_3CN : H_2O；8-9min 90:10 CH_3CN : H_2O；9-10min 60:40 CH_3CN : H_2O；Flow rate 1.0 mL / min)

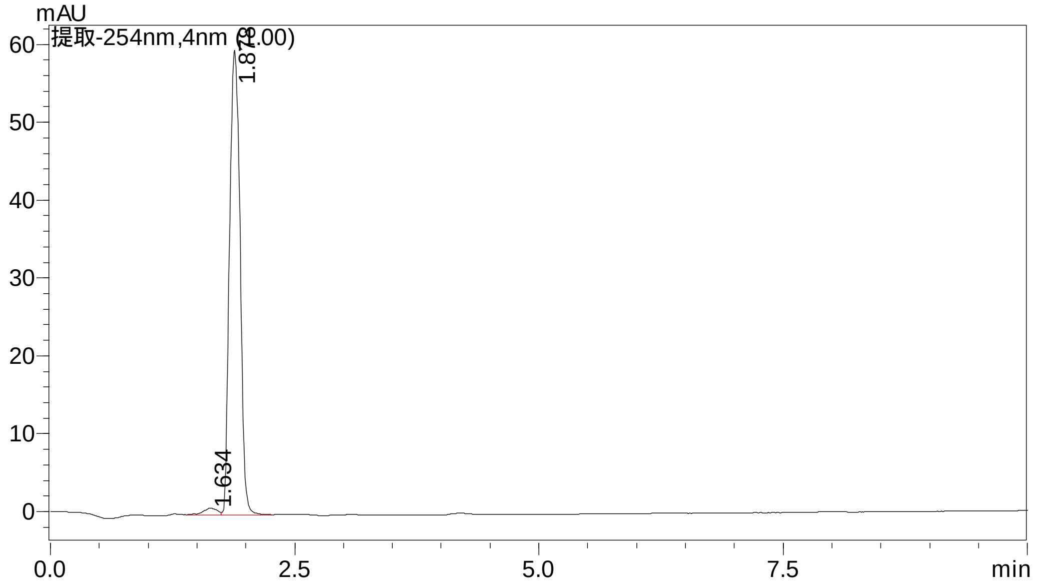

No.	Retention Time	Peak height	Area	Percent
1	1.634	841	8534	1.8554
2	1.878	59645	451457	98.1446
Total				100

QDAU-5

Reverse Phase (Method：0-8min 60:40 CH_3CN : H_2O；8-9min 90:10 CH_3CN : H_2O；9-10min 60:40 CH_3CN : H_2O；Flow rate 1.0 mL / min)

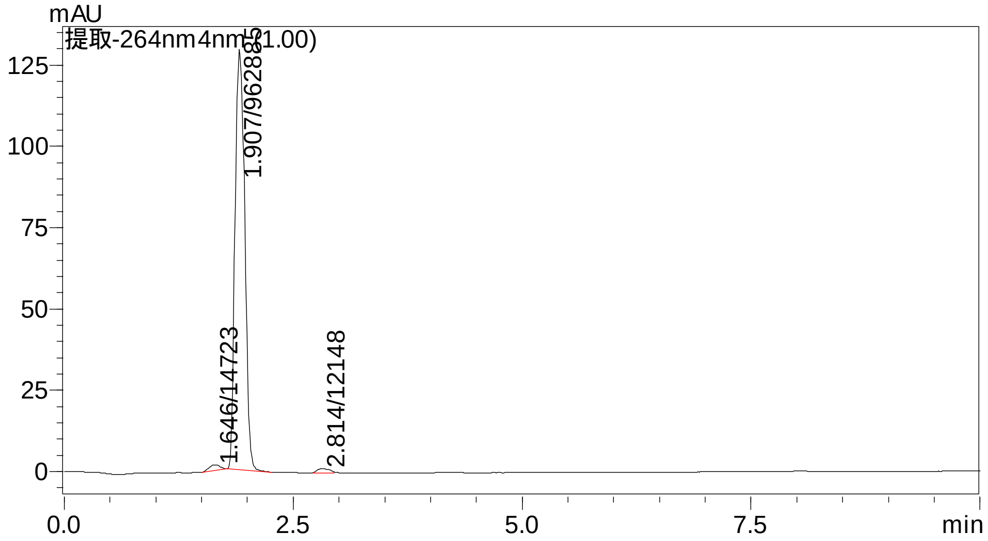

No.	Retention Time	Peak height	Area	Percent
1	1.646	1706	14723	1.4876
2	1.907	129138	962885	97.2850
3	2.814	1319	12148	1.2274
Total				100

QDAU-6

Reverse Phase (Method 90:10 CH_3CN : H_2O, Flow rate 1.0 mL / min)

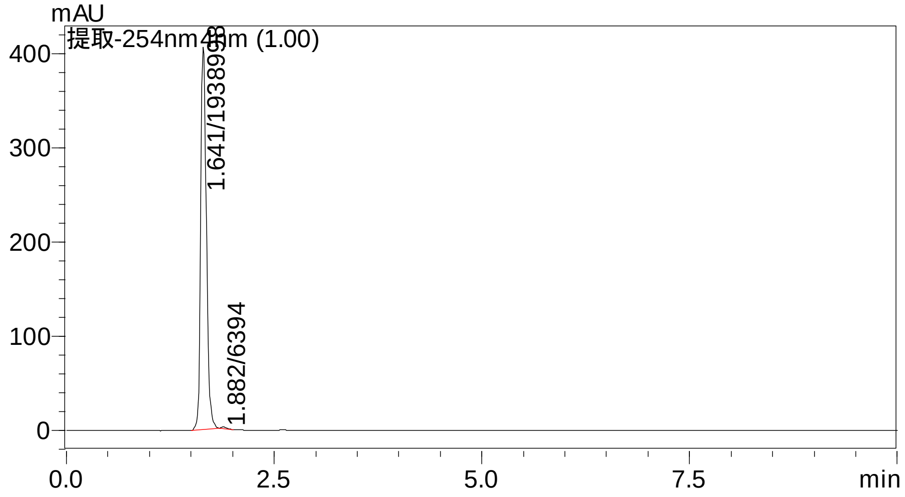

No.	Retention Time	Peak height	Area	Percent
1	1.641	405577	1938998	99.6713
2	1.882	1656	6394	0.3287
Total				100

QDAU-7

Reverse Phase (Method 90:10 CH_3CN : H_2O, Flow rate 1.0 mL / min)

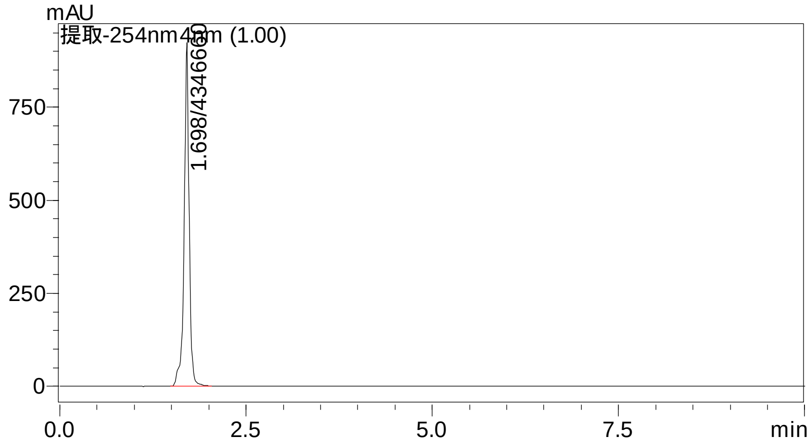

No.	Retention Time	Peak height	Area	Percent
1	1.698	923252	4346660	100.0000
Total				100

QDAU-9

Reverse Phase (Method：0-8min 60:40 CH_3CN : H_2O；8-9min 90:10 CH_3CN : H_2O；9-10min 60:40 CH_3CN : H_2O；Flow rate 1.0 mL / min)

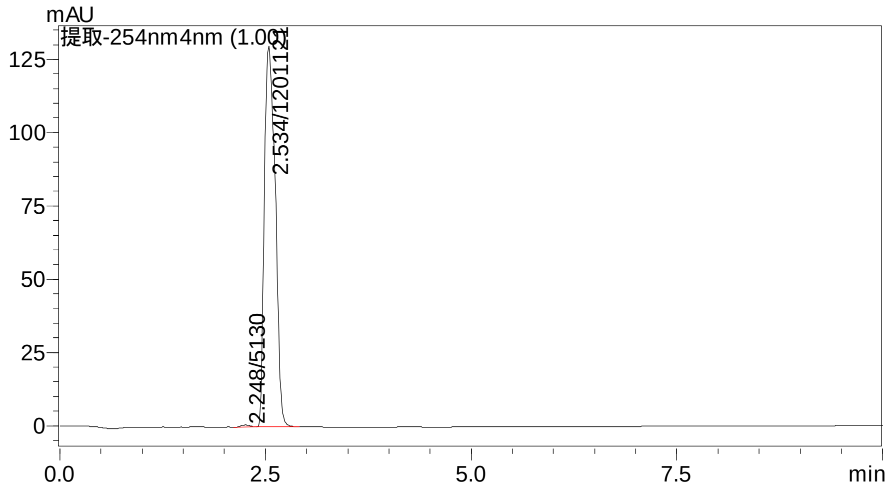

No.	Retention Time	Peak height	Area	Percent
1	2.248	686	5130	0.4253
2	2.534	129786	1201121	99.5747
Total				100

QDAU-10

Reverse Phase (Method：0-8min 60:40 CH_3CN : H_2O；8-9min 90:10 CH_3CN : H_2O；9-10min 60:40 CH_3CN : H_2O；Flow rate 1.0 mL / min)

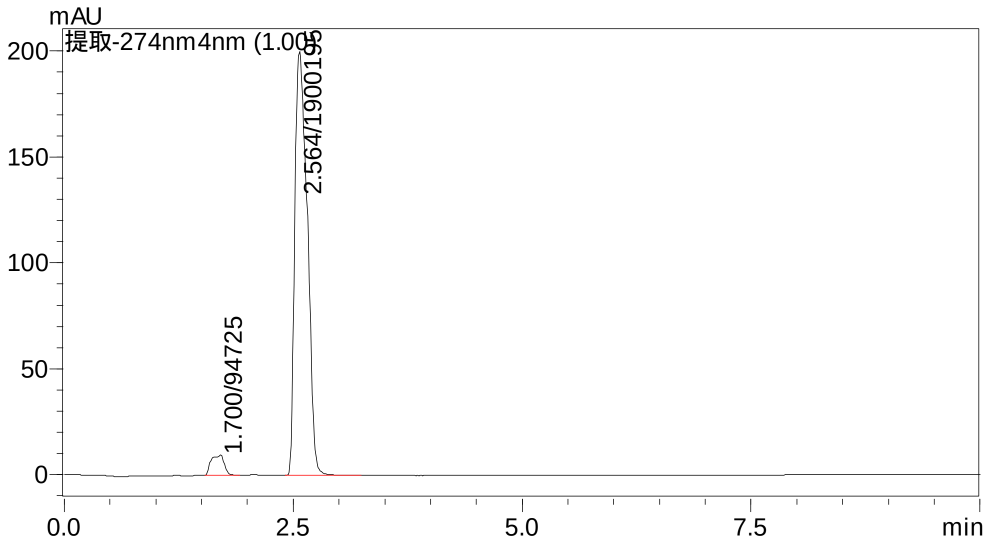

No.	Retention Time	Peak height	Area	Percent
1	1.700	9716	94725	4.7483
2	2.564	200049	1900195	95.2517
Total				100
